# The interferon-inducible p47 (IRG) GTPases in vertebrates: loss of the cell autonomous resistance mechanism in the human lineage

**DOI:** 10.1186/gb-2005-6-11-r92

**Published:** 2005-10-31

**Authors:** Cemalettin Bekpen, Julia P Hunn, Christoph Rohde, Iana Parvanova, Libby Guethlein, Diane M Dunn, Eva Glowalla, Maria Leptin, Jonathan C Howard

**Affiliations:** 1 Institute for Genetics, University of Cologne, Zülpicher Strasse 47, 50674 Cologne, Germany; 2 Eccles Institute of Human Genetics, University of Utah, Salt Lake City, UT 84112-5330, USA; 3 Informatics & Systems Groups, Sanger Centre, The Wellcome Trust Genome Campus, Hinxton, Cambridge, CB10 1SA UK; 4 Department of Structural Biology, Stanford University Medical School, Stanford, CA 94305, USA; 5 Institute for Microbiology and Immunology, University of Cologne Medical School, 50935 Cologne, Germany

## Abstract

**Background:**

Members of the p47 (immunity-related GTPases (IRG) family) GTPases are essential, interferon-inducible resistance factors in mice that are active against a broad spectrum of important intracellular pathogens. Surprisingly, there are no reports of p47 function in humans.

**Results:**

Here we show that the p47 GTPases are represented by 23 genes in the mouse, whereas humans have only a single full-length p47 GTPase and an expressed, truncated presumed pseudo-gene. The human full-length gene is orthologous to an isolated mouse p47 GTPase that carries no interferon-inducible elements in the promoter of either species and is expressed constitutively in the mature testis of both species. Thus, there is no evidence for a p47 GTPase-based resistance system in humans. Dogs have several interferon-inducible p47s, and so the primate lineage that led to humans appears to have lost an ancient function. Multiple p47 GTPases are also present in the zebrafish, but there is only a tandem p47 gene pair in pufferfish.

**Conclusion:**

Mice and humans must deploy their immune resources against vacuolar pathogens in radically different ways. This carries significant implications for the use of the mouse as a model of human infectious disease. The absence of the p47 resistance system in humans suggests that possession of this resistance system carries significant costs that, in the primate lineage that led to humans, are not outweighed by the benefits. The origin of the vertebrate p47 system is obscure.

## Abstract 2

A survey of p47 GTPases in several vertebrate organisms shows that humans lack a p47 GTPase-based resistance system, suggesting that mice and humans deploy their immune resources against vacuolar pathogens in radically different ways.

## Background

It is generally assumed that the immune system of the mouse is a good experimental model for that in humans. However, several studies suggest that immune mechanisms have been evolving rather differently in the human and mouse lineages (for review, see Mestas and Hughes [1]). The p47 (immunity-related GTPases (IRG) family; see Nomenclature, below) GTPases present a uniquely striking example of this divergence.

In mice the interferon-γ-inducible p47 GTPases constitute one of the most powerful resistance systems against several important intracellular pathogens [2-4]. The proteins localize on intracellular membrane systems in interferon-induced cells, some (IGTP, IIGP1) favoring the endoplasmic reticulum [5,6] and others (LRG-47, GTPI) the Golgi membranes [6,7] (for names of individual IRG GTPases see Additional data file 1). Infection or phagocytosis, however, initiates redistribution of the p47 GTPases to the phagocytic vacuole [6-8]. The p47 GTPases probably act specifically against vacuolar pathogens. Thus, Gram-positive and Gram-negative bacteria, mycobacteria, and protozoal pathogens are all resisted by the p47 GTPases, whereas no viral target has yet been confirmed.

The p47 GTPase IIGP1 is a low-affinity nucleotide binding protein with a slow GTP turnover [9]. At high protein concentrations and in the presence of GTP, IIGP1 oligomerizes and increases GTP turnover by up to 20-fold. These properties are distinct from those of the classical signaling GTPases and are reminiscent of the dynamins and p65 (GBP-1) GTPases [10,11]. The crystal structure of IIGP1 exhibits a H-Ras-1-like nucleotide-binding domain flanked by amino-terminal and carboxyl-terminal helical domains that are unknown in other GTPases [12]. This basic structure is probably common to the whole family. However, the divergent sequences of published p47 GTPases [13] and the patterns of susceptibility in knockout strains (for reviews, see Taylor [2] and MacMicking [3,4]) show that the proteins are highly diversified. Thus, a subgroup of three proteins (the GMS GTPases) have a radical substitution (the substitution of Methinine (M) for Lysine (K)) in the conserved P-loop G1 motif of the nucleotide binding site (Walker A motif) and correlated sequence variation elsewhere in the G-domain [13], implying a distinct catalytic mechanism for GTP hydrolysis. In the case of IIGP1 and LRG-47, the cell biology of the two proteins is distinct; IIGP1 associates with the endoplasmic reticulum membrane primarily through an amino-terminal myristoylation sequence, whereas LRG-47 associates with Golgi membrane via an amphipathic helix in the subterminal domain [6]. We recently showed that IIGP1 participates in a novel effector mechanism in *Toxoplasma gondii *infected astrocytes involving vesiculation and ultimately destruction of the parasitophorous vacuole membrane [8]. In contrast, there is evidence that LRG-47 is involved in accelerated acidification of the phagocytic vacuole containing *Mycobacterium tuberculosis *[8].

The p47 GTPases are thus a functionally diverse resistance system with many signs of adaptive divergent evolution. Surprisingly, there are no reports of p47 GTPase function in humans. To address this imbalance, we analyzed the p47 GTPase gene family in depth. We conclude that although the mouse has 23 p47 GTPases, of which up to 20 may be functional in resistance, the resistance system is entirely absent from humans. This finding carries important implications for our understanding of human and mouse immunity to vacuolar pathogens.

## Results

### Genomic organization of the p47 GTPase (*Irg*) genes of the C57BL/6 mouse

**Figure 1:**
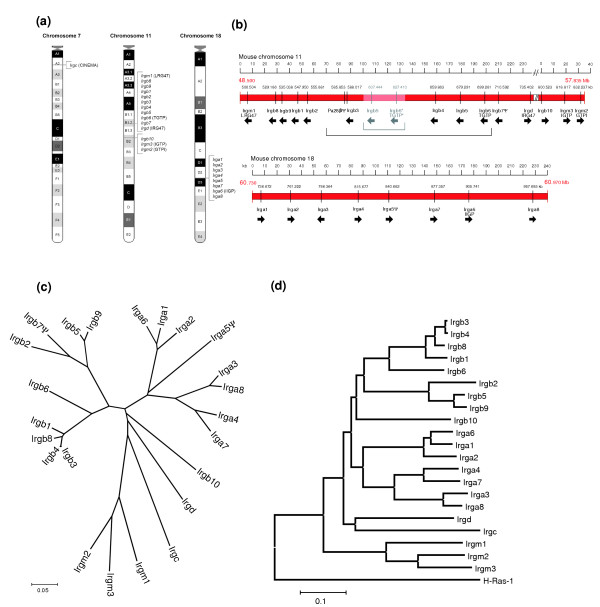
Genomic positioning and phylogenetic relationship of mouse Irg GTPases. **(a) **Disposition of the 23 *Irg *genes on the mouse karyotype. Individual *Irg *genes are listed in correct gene order in each cluster. **(b) **Positioning and orientation of *Irg *genes in the mouse chromosome 11 and 18 clusters. Positions of genes refer to the location in Mouse ENSEMBL release (v28.33d.1, February 2005) [61] of the first G of the glycine codon of the G1 motif (GKS or GMS) of the GTP-binding domain of each gene. The segments of the chromosome 11 cluster indicated with square brackets are regions of uncertain structure. Gene orientation is given by black arrows. The shaded region of the chromosome 11 map is a duplication introduced in Mouse ENSEMBL v28.33d.1 (February 2005) in an attempt to resolve a region of high ambiguity indicated by the longer square bracket. In our view this duplication does not resolve the ambiguities consistently, and we see no justification at present for the duplicated *Irgb5 *and *Irgb6 *genes. The sibling genes *Irgb3 *and *Irgb4 *differ by only nine nucleotides; in this case, however, the independent existence of the two genes is proved by the proximity of the PA28βψ retropositioned pseudo-gene to *Irgb3 *but not to *Irgb4*, in addition to consistent sequence differences. We have left the duplication of the *Irgb5/Irgb6 *region in the map for consistency of the base numbering with this release of ENSEMBL. *Indicates minor sequence differences presumably due to sequencing errors. **(c) **Unrooted tree (p-distance based on neighbour-joining method) of nucleotide sequences of the G-domains of the 23 mouse Irg GTPases, including the two presumed pseudo-genes *Irga5 *and *Irgb7*. The sources of all *Irg *sequences are given in Additional data file 1, and the nucleotide and amino acid sequences themselves are collected in the p47 (IRG) GTPase database from our laboratory website [14]. **(d) **Phylogenetic tree of the amino acid sequences of the G-domains of 21 mouse Irg GTPases rooted on the G-domain of H-Ras-1 (accession number: P01112). The products of the two presumed pseudo-genes *Irga5 *and *Irgb7 *are excluded from the analysis.

There are 23 p47 GTPase (*Irg*) genes in the C57BL/6 mouse, including the six previously known members of the family [13], localized on chromosomes 7, 11 and 18 (Figure 1a,b; also see Figure 7a). (For the nomenclature of the *Irg *genes, see Nomenclature (below) and Additional data file 1). Two of the mouse *Irg *sequences, namely *Irga5 *and *Irgb7*, are clearly pseudo-genes (see legend to Figure 1b). The remaining 21 *Irg *genes are intact across the GTP-binding domain, although *Irga1*, *Irga8*, and *Irgb10 *are carboxyl-terminally truncated relative to the majority, and no transcripts of *Irga7 *and *Irgb8 *have yet been found. Thus, the number of potentially functional *Irg *genes is not six but rather 21 in the C57/BL6 mouse. The nucleotide and protein sequences of these genes can be found on our home page [14].

The complex block of 13 genes on chromosome 11 contains the most divergent sequences (Figure 1c,d; Additional data file 2), including all three GMS (*Irgm*) GTPases [13], suggesting that this cluster is relatively ancient. In contrast, the eight *Irga *genes clustered on chromosome 18 are also clustered phylogenetically, suggesting more recent divergence, probably from a translocated member of the *Irgb *(TGTP) cluster on chromosome 11. The isolated *Irg *gene on chromosome 7, *Irgc*, is an ancient root with no obvious systematic relationship to the other subfamilies. Within the chromosomal clusters, more recent duplication events are apparent. The sibling pair *Irgb3 *and *Irgb4 *differ by only nine nucleotides in the open reading frame. The genes *Irgb1*, *Irgb3*, *Irgb4*, and *Irgb8 *appear to have been duplicated in tandem with *Irgb2*, *Irgb5*, and *Irgb9*, respectively. The pattern of divergence in the mouse p47 tree suggests an old gene family that has undergone a succession of duplication-divergence cycles over time - a pattern of evolution that is still actively continuing in several of the subfamilies.

### The structure of p47 GTPase genes and their splicing patterns

**Figure 2:**
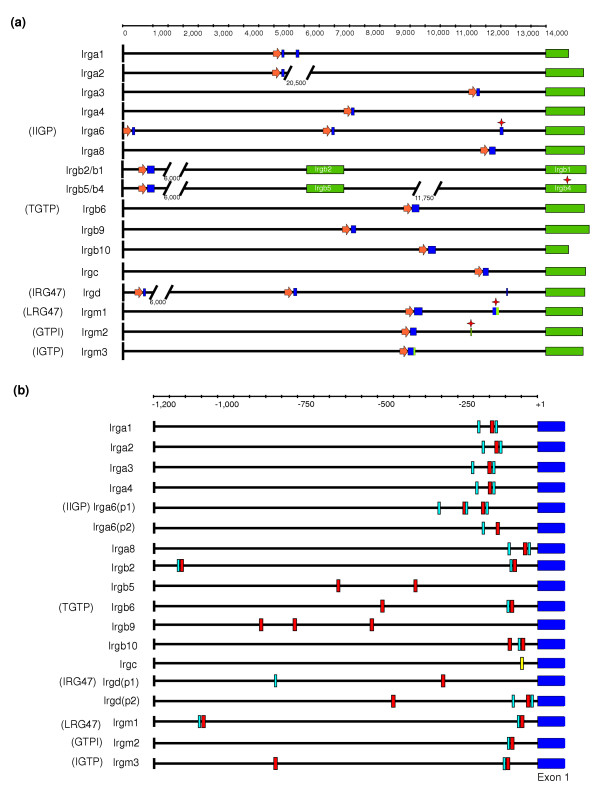
Genomic and promoter structure of mouse Irg GTPases. **(a) **Genomic structure of mouse *Irg *genes. Green blocks indicate coding exons and blue blocks indicate 5'-untranslated exons. Orange arrows identify putative promoter regions. Stars identify exons shown to be excluded in alternative splice forms. The scale bar is measured in base pairs up to the first base of the long coding exon. Note the presence of two promoters for *Irga6 *and *Irgd*. **(b) **Interferon response elements in the promoter regions of mouse *Irg *genes. γ-Activated sequences (GAS; pale blue blocks) and interferon-stimulated response element (ISRE; red blocks) sequences were identified in the promoters shown in panel a (also see Additional data file 7). Dark blue blocks downstream of each promoter represent the most 5' exon. The yellow block identifies a putative Sox1 transcription factor binding site in the proximal promoter region of *Irgc*. The scale bar is measured in base pairs from the first base of the 5' exon.

The open reading frame of *Irg *genes is typically encoded on a single long 3' exon (Figure 2a) behind one or more 5'-untranslated exons. However, in one splice form of *Irgm1 *and one splice form of *Irgm2 *the initial methionine is encoded at the 3' end of the penultimate exon (also see the legend to Figure 2). The closely related *Irgb1 *and *Irgb4 *genes are exceptional in apparently occurring only as tandem transcripts in-frame with their respective closely linked upstream genes *Irgb2 *and *Irgb5*. If translated, such transcripts would generate 94 kDa polypeptides containing two distinct full-length p47 GTPase units. For the sequence phylogenies and alignments (Figure 1c,d; also see Figure 4, below), we provisionally treat these separate p47 units as independent genes. It remains to be seen whether the third tandem gene pair, *Irgb9 *and *Irgb8*, is also expressed as a tandem transcript. That *Irgb1*, *Irgb3*, and possibly *Irgb8 *are normally expressed in tandem with an upstream gene is also consistent with the absence both of autonomous transcripts of these exons and of interferon-inducible promoter elements (see below).

### Identification of interferon-stimulatable elements in putative promoters of *Irg *genes

**Figure 3:**
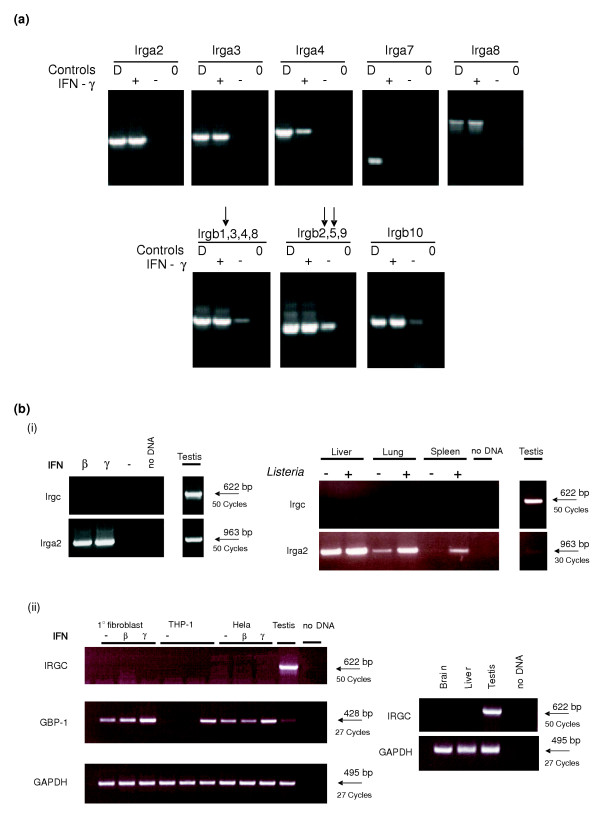
Interferon responsiveness of mouse and human p47 (IRG) GTPase. **(a) **Interferon (IFN)-γ responsiveness of eight new mouse *Irg *genes. Inducibility of eight further *Irg *genes (also see Boehm and coworkers [13]) in L929 fibroblasts induced for 24 hours with IFN-γ, demonstrated by RT-PCR. D refers to a positive control genomic DNA template; O refers to a negative control of the same genomic template after DNAse1 treatment; and + and - refer to RT-PCR on DNAse1-treated RNA templates from IFN-γ-induced and IFN-γ-noninduced cells, respectively. The sibling genes of the *Irgb *series could not be individually amplified because of their close sequence similarity. The identities of the amplified genes responding to interferon induction, indicated by vertical arrows, were subsequently established by sequencing of multiple clones from the PCR product. **(b) ***Irgc *is not induced by interferon or infection but is constitutively expressed in testis. (i, left) Mouse L929 fibroblasts were induced for 24 hours with IFN-β or IFN-γ or left uninduced (-). *Irgc *could not be detected by RT-PCR even after 50 amplification cycles in L929 cells. *Irga2 *after 50 cycles was used as a positive control for the interferon-induced L929 RNA. RNA from mouse testis provided a positive control for *Irgc*. (i, right) RT-PCR for *Irgc *and *Irga2 *(50 and 30 amplification cycles respectively) on RNA from tissues of uninfected mice (-) or mice infected 24 hours previously with *Listeria monocytogenes *(+). *Irga2 *was induced in all tissues and *Irgc *in none. RNA from mouse testis provided a positive control for *Irgc*, which is detected after 50 cycles. Testis expression of *Irga2 *was barely detected after 30 cycles (compare with i, left, showing *Irga2 *in testis after 50 cycles). (Panel ii, left) Human *IRGC *is not induced by 24 hours of stimulation with IFN-β or IFN-γ in human cell lines (induction of GBP-1 [accession number P32455] was assayed as a positive control) and (Panel ii, right) is constitutively expressed only in human testis. GAPDH was used as control.

The basis for interferon-inducible expression of the mouse p47 GTPases has previously been investigated only for *Irgd *(IRG47) [15], in which an active interferon-stimulated response element (ISRE) was found upstream of the putative transcriptional start point. A GAS (γ-activated sequence) site was predicted in the putative promoter region of *Irgm1 *(LRG-47) [8]. Most of the transcribed p47 genes on chromosomes 11 and 18 exhibit multiple perfect interferon-inducible genomic motifs, both ISRE and GAS elements (Figure 2b; Additional data file 3). The sequences and relative positions of the GAS and ISRE elements vary, both classes of site are not present in all promoters, and the orientations of the two components are also variable. Thus, the association of interferon-inducible elements with *Irg *genes is presumably ancient and has been retained against the disruptive forces of spontaneous genome evolution. No further immunity-related inducible elements such as NFκB sites were found to be associated with the ISRE/GAS motifs. *Irgd *and *Irga6 *are both transcribed from alternative 5'-untranslated exons, each furnished with an independent promoter. In both genes the initial methionine is encoded at the beginning of the long 3' exon, so that the two transcripts of each gene generate identical proteins. Both putative promoters of *Irgd *and *Irga6 *have interferon-inducible elements. As noted above, genes *Irgb1*, *Irgb4*, and *Irgb8 *are probably expressed only as the 3' ends of tandem transcripts with *Irgb2*, *Irgb5*, and *Irgb9*, respectively. No dedicated 5'-untranslated exons could be identified for these downstream domains. Using RT-PCR we were able to show clear induction of eight further genes (*Irga2*, *Irga3*, *Irga4*, *Irga8*, *Irgb1*, *Irgb2*, *Irgb5*, and *Irgb10*) in addition to the six (*Irga6 *(IIGP), *Irgb6 *(TGTP), *Irgd *(IRG-47), *Irgm1 *(LRG-47), *Irgm2 *(GTPI) and *Irgm3 *(IGTP)) assayed by Boehm and coworkers [13] in L929 fibroblasts stimulated with interferon-γ *in vitro *(Figure 3a).

The isolated p47 gene, *Irgc*, on chromosome 7 is a clear exception. No clustered or isolated ISRE or GAS elements could be identified up to 10 kilobases (kb) 5' of the putative transcription start of this transcribed gene, and *Irgc *was not induced in interferon-stimulated fibroblasts (Figure 3b, panel i left). A weak Sox-related element was detected in the proximal promoter region. In view of the close homology of *Irgc *to the interferon-inducible *Irg *genes, we considered whether *Irgc *is induced in tissues of mice 24 hours after infection with *Listeria monocytogenes *[13,16]. No induction of *Irgc *was detected in liver, lung, or spleen after 50 cycles of amplification, whereas *Irga2*, used as a positive control, was induced in all three tissues (Figure 3b; panel i right). However, *Irgc*, unlike *Irga2*, was constitutively expressed in the mature mouse testis (Figure 3b; unpublished data). We conclude that mouse *Irgc *is expressed in a tissue-specific manner and is not induced by infection.

### The coding sequences of the p47 GTPases

**Figure 4:**
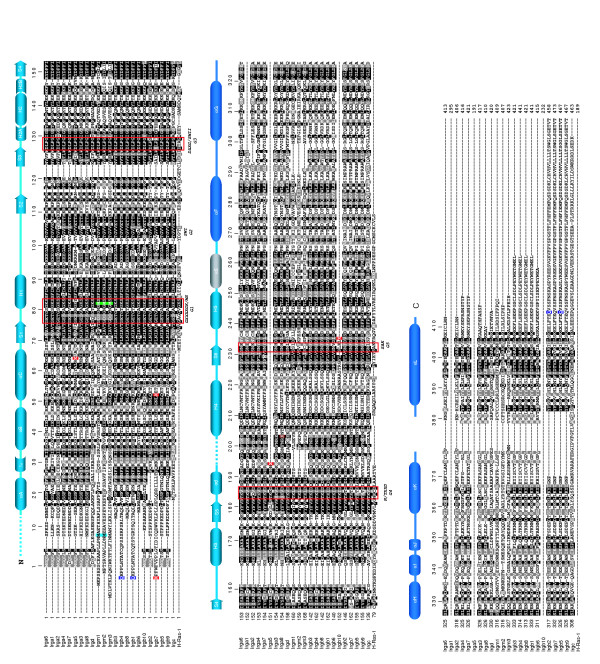
Amino acid alignment of the mouse Irg GTPases. Sequences of all 23 mouse Irg GTPases showing the close homology extending to the carboxyl-terminus, aligned on the known secondary structure of Irga6 (indicated in blue above sequence alignment). The sequences of notional products of the two pseudo-genes *Irga5 *and *Irgb7 *have been partially reconstructed; premature terminations are indicated by red highlighting. In the C57BL/6 mouse the sequence of the *Irga8 *gene is damaged by an adenine insertion, indicated by the red highlighted K at position 204. (The sequence given after this point is that given after correcting the frameshift, and is identical to that of the CZECHII [*Mus musculus musculus*] sequence BC023105 that lacks the extra adenine.) The turquoise-highlighted M in M1 and M2 are initiation codons that are dependent on alternative splicing (also see Figure 2a); the unusual methionine residues in the G1 motif of GMS proteins are highlighted in green. The blue background Q residue of Irgb5 and Irgb2 at positions 405 and 396 indicate the point at which tandem splicing occurs to Irgb4 and Irgb1, respectively. Canonical GTPase motifs are indicated by red boxes.

In Figure 4 we present the predicted translation products of the 21 intact p47 GTPase genes, and reconstructed partial sequences of the two pseudo-genes, Irga5ψ and Irgb7ψ, aligned on the secondary structures of Irga6 [12] and H-Ras-1 [17]. The full alignment confirms a number of major features that are already apparent from the previously published alignment of six family members [13] and consolidates the definition of the p47 GTPases as a distinctive sequence family. Especially noteworthy are novel features of the amino- and carboxyl-termini, which were not apparent before. Eleven of the proteins, including six of chromosome 18 Irga gene products and Irgb2, Irgb5, Irgb9 and Irgb10, carry the amino-terminal myristoylation signal MGxxxS [18]. This sequence in Irga6 (IIGP1) is indeed myristoylated *in vitro *[19] and *in vivo*, and, as expected, favors binding of the protein to membranes [6]. No other membrane attachment sequences or lipid modification motifs are apparent in p47 GTPase sequences, despite the documented attachment of several of these proteins to membranes [5,6,16]. Irgb2, Irgb5, Irgb7, Irgb9 and Irgc have carboxyl-terminal extensions up to 65 residues in length compared with the canonical IIGP1 sequence.

### The p47 GTPase genes of the human genome

**Figure 5:**
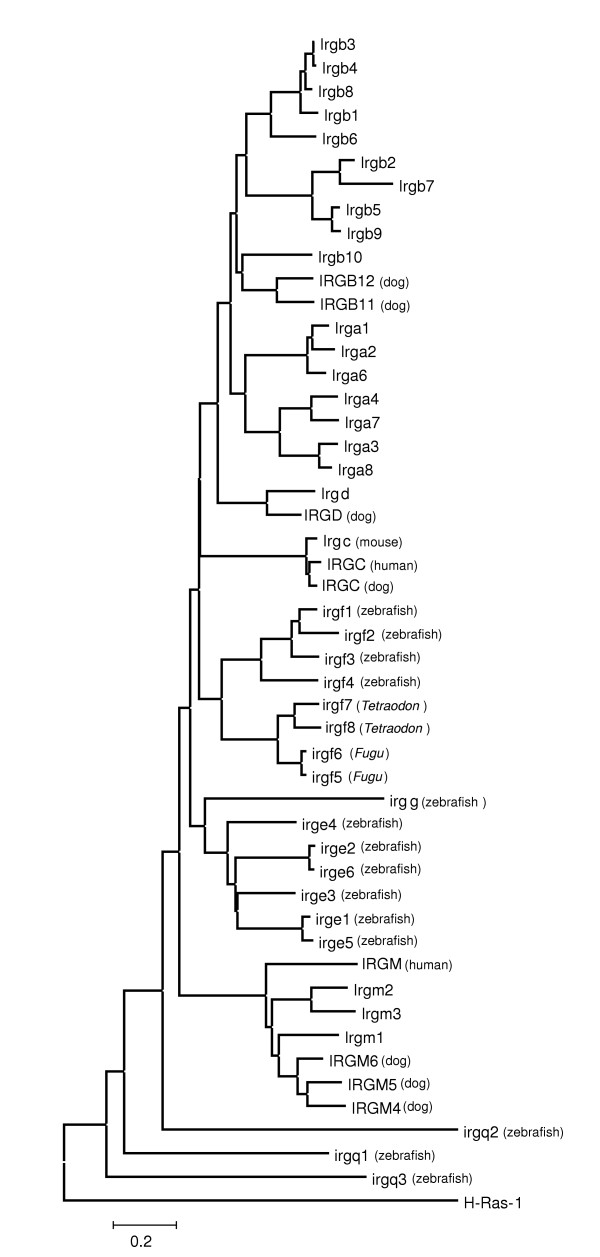
Extended phylogeny of the G domains of IRG and related proteins. The phylogeny relates all of the IRG sequences described in this report and reveals the distinct clades on which the nomenclatural fine structure is based. All except the mouse sequences are labeled with the species of origin. Dog IRG sequences are found in the B, C, D and M clades, and human sequences only in clades C and M. The mouse and human quasi-IRG proteins, IRGQ (FKSG27), could not be included in the phylogeny because they are so deviant in the G-domain (see Figure 6 and Additional data file 6).

**Figure 6:**
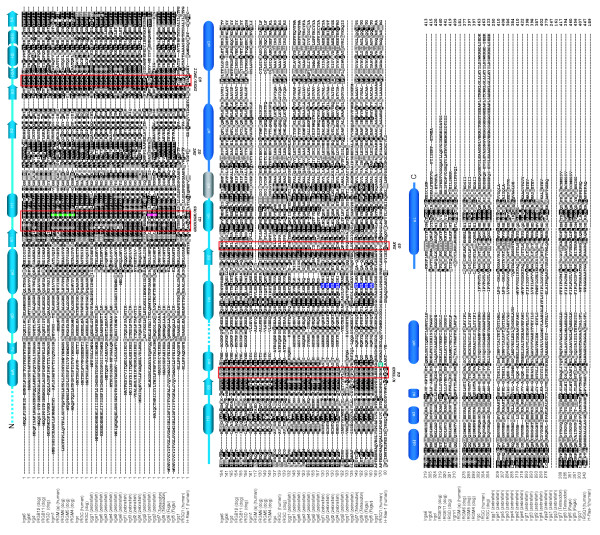
Extended alignment of the vertebrate IRG proteins. Individual sequences are given in full and are labeled as in Figure 5. Unusual residues in the G1 motif are highlighted (M of the GMS proteins in green and two deviant residues in the zebrafish irgq sequences in pink). The essential structural relationship between IRG genes and quasi-IRG genes is apparent in the alignment despite the modified G-domains. For mouse and human IRGQ the long carboxyl-terminal coding exons that contain the p47 homology were used for the alignment. In human IRGQ the sequence ENPKGESLKNAGGGGLENALSKGREKCSAGSQKAGSGEGP was removed from the alignment between positions 210 and 211 (highlighted in turquoise) to prevent extensive gap formation. The position of the intron present in pufferfish and zebrafish *irgf *genes is indicated by two adjacent residues highlighted in blue.

Only two IRG sequences, both transcribed, are present in humans (or chimpanzee), one (*IRGC*) on chromosome 19 (19q13.31) and the other (*IRGM*) on chromosome 5 (5q33.1). Human IRGC is more than 85% identical at the nucleotide level and 90% at the amino acid level to the isolated mouse gene *Irgc*. *IRGM *encodes an amino- and carboxyl-terminally truncated G-domain homologous to the Irgm (GMS) subfamily of mouse p47 GTPases. Predicted protein products of *IRGC *and the *IRGM *gene fragment are included in an extended phylogeny (Figure 5) and alignment (Figure 6) of the vertebrate IRG proteins.

**Figure 7:**
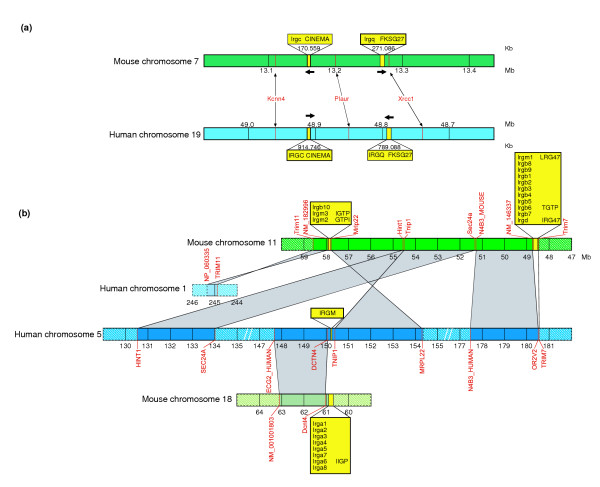
Synteny relationships between the human and mouse *IRG *genes **(a) **Synteny between mouse chromosome 7 and human chromosome 19 in the region of the *IRGC *and *IRGQ *genes. The figures indicate distances from the centromere in megabases. The locations of three further syntenic markers are given. Gene orientation is given by black arrows. **(b) **Complex synteny relationship between human chromosome 5 and mouse chromosomes 11 and 18 in the regions containing the mouse *Irg *genes. Figures indicate distances from the centromere in megabases. The locations of *IRG *genes are shown in the yellow panels. Positions of diagnostic syntenic markers are also indicated. Syntenic blocks are given in full color, and the rest is shaded.

The *IRGC *mouse and human genes sit in chromosomal regions syntenic between chromosomes 7 and 19, respectively (Figure 7a) and are clearly orthologous. The proximal promoter region of human *IRGC *is largely conserved with that of mouse *Irgc*. However, as in the mouse, no interferon response elements are found either in the proximal conserved region or in divergent regions up to 10 kb upstream of the transcriptional start (data not shown). Human IRGC, like mouse Irgc, is not inducible *in vitro *by interferons, is not expressed detectably in brain or liver, but is strongly expressed in adult testis (Figure 3b, panel ii). As in the mouse, a weak Sox element is present in the proximal promoter of human *IRGC*.

**Figure 8:**
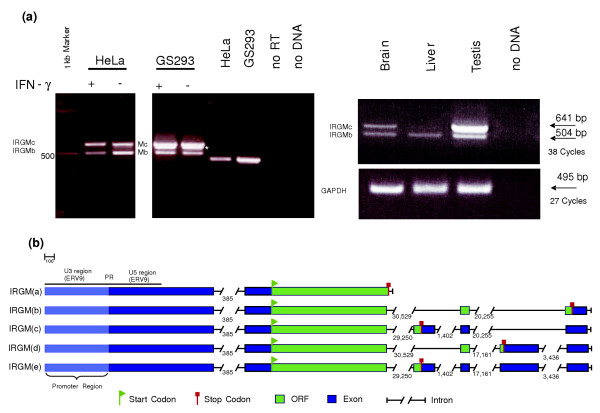
Structure and expression of the human *IRGM *gene. **(a) **(left panels) RT-PCR analysis of expression of *IRGM *in HeLa and GS293 cells. The b and c splice variants were amplified simultaneously by the same primer pair (IRGMs1-rGMS). A different downstream primer (IRGMs1-r1) internal to all the 3' splice forms was used to show differences in the overall expression level of *IRGM *in the two cell lines. No RT' indicates that no reverse transcriptase is included in cDNA preparation. The band immediately below the IRGMc band in GS293 cell material, indicated with an asterisk, is a nonspecific band amplified only in this cell line. The band was sequenced and is unrelated to IRGM. (right panel) Analysis of *IRGM *expression in human brain, liver and testis by RT-PCR. GAPDH was used as a control. **(b) **Five splice forms of the *IRGM *gene have been identified, as indicated: IRGM(a)-IRGM(e). The promoter and 5'-untranslated regions of the gene are associated with an ERV9 retroviral LTR. Scale-bar is given in base pairs.

The human genomic segments syntenic to the mouse chromosome 11 and chromosome 18 *IRG *gene clusters both mapped to human 5q33.1, suggesting that the interferon-inducible IRG proteins were once encoded in a single block ancestral to the human chromosome 5 region (Figure 7b). *IRGM *maps only 80 kb away from the closest syntenic marker *DCTN4*. *IRGM *is transcribed in unstimulated human tissue culture lines HeLa and GS293 (Figure 8a), with no increase after interferon induction. Polyadenylated transcripts of *IRGM *occur with five 3' splicing isoforms extending more than 50 kb 3' of the long coding exon (Figure 8b). The transcripts have a 5'-untranslated region of more than 1,000 nucleotides that corresponds largely to the U5 region of an ERV9 repetitive element [20]. The promoter region corresponds to the ERV9 U3 LTR (long terminal repeat) without interferon response elements, and three of the five splice forms have exon-intron boundaries downstream of the putative termination codon, normally a signal for rapid RNA degradation [21].

At the protein level the shortest isoform of IRGM is shorter than a canonical G-domain, being truncated in the middle of β-strand 6 just before the G5 sequence motif, which interacts with the guanine base of the bound nucleotide (Figures 6 and 8b; also see Ghosh and coworkers [12]). The longer isoforms are terminated by short sequence extensions that are unrelated to known GTPase domains. A rabbit antiserum raised against recombinant human IRGM produced in *Escherichia coli *failed to detect signal by immunofluorescence or Western blot in human cell lines (data not shown).

### *IRG *genes of the dog

Is the mouse (order Rodentia) or the human (order Primata) the exception? We looked for *IRG *genes in a third order of mammals, the Carnivora. We recovered a total of eight *IRG *genes from the public genome database of the dog *Canis familiaris *(Figures 5 and 6) as well as a partial sequence of a 9th gene (not shown). Of these, one (not shown) is a pseudo-gene by a number of criteria, another is clearly dog *IRGC*, whereas the partial sequence is novel but most closely related to *IRGC*. The remainder assort into segments of the phylogeny already established for the interferon-inducible mouse *IRG *genes (Figure 5). Both GMS and GKS genes are represented and are inducible by interferon in dog MDCK epithelial cells (Additional data file 4). The three dog GMS genes appear to have diversified independently from the mouse GMS genes (Figure 5). As in humans and mouse, dog *IRGC *was not induced by interferon-γ (Additional data file 4). Overall, the *IRG *gene status of the dog clearly resembles that of mouse rather than that of humans.

### *IRG *genes in fish genomes

IRG GTPases are at least as old as the vertebrates. We have identified at least two distinct *irg *genes in the freshwater pufferfish *Tetraodon nigriviridis*, a closely linked pair of *irg *genes in the saltwater pufferfish *Fugu rubripes*, and at least 11 partially clustered *irg *genes in the zebrafish *Danio rerio *(Figures 5 and 6, and Additional data file 5). The fish *irg *genes fall into separate clades from the mammalian genes (Figure 5). A specific *IRGC *homolog is not immediately apparent. GMS subfamily *IRGM *genes are absent from fish. The pufferfish and zebrafish *irgf *genes have one intron identically positioned at the end of helix 4 of the G-domain (indicated on Figure 6; also see Additional data file 5). This intron is 81 bp long in both pufferfish species but is substantially longer in the zebrafish genes. The distinct *irge *subfamily of the *Danio irg *genes are intronless in the open reading frame, like mammalian *IRG *genes.

### *IRG *homologs with divergent nucleotide-binding regions: the quasi-GTPases

The mouse, human and zebrafish genomes encode proteins that are homologous to the *IRG *GTPases but are radically modified in the GTP-binding site. The mammalian protein FKSG27 (IRGQ), a protein of unknown function that is 70% conserved between man and mouse, is extended amino-terminally relative to a p47 GTPase by about 100 residues encoded on three short exons. The remaining 420 residues, encoded on a single long exon, are clearly homologous to and colinear with the IRG proteins (Figure 6 and Additional data file 6), especially in the amino- and carboxyl-terminal parts of the exon. The region of lowest similarity is in the G-domain, and conserved GTP-binding motifs are lacking (Figure 6, and Additional data files 6 and 7). Thus, FKSG27 (IRGQ) is not a GTPase despite its phylogenetic relationship to the *IRG *proteins. FKSG27 (IRGQ) is closely linked to *IRGC *in humans and mouse (Figure 7a).

The zebrafish genome contains three *IRG *homologs with more or less modified GTP-binding motifs (*irgq1*-*irgq3*; Figures 5 and 6, and Additional data file 7). Their homology to *IRG *genes is stronger than that of FKSG27 (IRGQ), but as with FKSG27 (IRGQ) their function as GTPases is doubtful. The *irgq1 *gene is clustered on a single BAC clone with four apparently normal *irge *genes and immediately downstream of a truncated p47 gene, *irgg*, with which *irgq1 *is transcribed as the carboxyl-terminal half of a tandem transcript. Thus, the hypothetical protein product would be a carboxyl-terminally truncated p47 GTPase, linked at its carboxyl-terminus to a similarly truncated p47 homolog probably without GTPase function.

We propose to term the modified IRG proteins without GTPase function 'quasi IRG' proteins, hence IRGQ. IRGQ sequences reveal their phylogenetic relationship to the IRG proteins, but they are nevertheless more or less radically modified, primarily in the nucleotide binding site. In view of the substantial divergence between the *IRGQ *genes and functional p47 GTPases, it was unexpected not to find close homologs of the *Danio irgq *sequences in either the *Fugu *or *Tetraodon *genomes. The evolution and diversity of the *Danio irgq *genes is apparently linked to the evolution and diversity of the GTPase-competent IRG sequences.

### IRG homologs outside the vertebrates

No unambiguous IRG homologs have been found outside the vertebrates. However, two possibly related sequence were recovered from the *Caenorhabditis elegans *genome, and several groups of putative GTPases of unknown function exist in the bacteria that have sequence features reminiscent of IRG GTPases. Perhaps the most striking of these are found in the Cyanobacteria (see Additional data file 1 for accession numbers for these sequences). Among other features, all of these sequences have in common with the IRG GTPases the presence of a large hydrophobic residue in place of the familiar catalytic Q61 of H-Ras-1, but this feature is far from diagnostic for the IRG GTPases [22]. Despite several suggestive characteristics of these invertebrate and bacterial GTPase sequences, it is not possible on the basis of sequence criteria alone to establish their phylogenetic relationship with vertebrate IRG proteins.

## Discussion

The p47 GTPases (IRG proteins) are an essential resistance system in the mouse for immunity against pathogens that enter the cell via a vacuole. In this study we reached several unexpected conclusions about the evolution of the system. First, the IRG resistance system, despite its importance for the mouse, is absent from humans because it has been lost during the divergent evolution of the primates. Second, the IRG resistance system is at least as old as the bony fish but missing in the invertebrates. Finally, the IRG proteins appear to be accompanied phylogenetically by homologous proteins, here named IRGQ proteins, that probably lack nucleotide binding or hydrolysis function, and that may form regulatory heterodimers with functional IRG proteins. We consider these points in order.

The argument for the absence of the IRG resistance system in humans relies on several findings. The system is reduced from 23 genes in mouse to one full-length gene and a transcribed G-domain in humans, and the residual genes lack the character of functional resistance genes. Thus, *IRGC *is highly conserved in humans, dog and mouse, is not interferon or infection inducible, and is expressed constitively in mature testis. *IRGM*, although clearly derived from a typical GMS subfamily resistance gene, is transcribed constitutively from an endogenous retroviral LTR, is unresponsive to interferon, and appears to be structurally damaged in several ways.

We argue that the IRG resistance system has been lost from primates (the situation in chimpanzee is identical to that in humans; unpublished data) rather than gained by the murine rodents (including rat; unpublished data) on the following grounds. First, like the mouse, the dog genome has several complete, interferon-inducible *IRG *genes in addition to *IRGC*. Second, humans and chimpanzees possess a degraded member of the GMS subfamily of IRG proteins, confirming that this distinctive subfamily, present and functional as resistance genes in dog and mouse, was widely distributed at the origin of the mammalian radiation. Finally, the IRG system is present in bony fish, representing ancient vertebrates. Rapid expansion and contraction of multigene families associated with pathogen resistance has frequently been documented in both animals and plants [23-28]. In all of these cases, however, the resistance mechanism itself has been retained as its protein mediators have evolved or even, in the natural killer receptor case, been replaced by a different molecular species [29]. The IRG case may be different because here the resistance mechanism itself has apparently been lost during primate evolution.

It will be of interest and of considerable importance to analyze the different strategies by which humans, dog and mouse deploy resistance mechanisms effective against vacuolar pathogens. None of the known mechanisms active in humans against vacuolar pathogens, namely nitric oxide and oxygen radicals [30-32], tryptophan depletion [33,34], accelerated acidification by Rab5a [35], cation depletion [36-38] or autophagy [39,40], is missing from the mouse. Nevertheless, it remains possible that the distinctive resistance actions of the p47 GTPases [7,8] are performed by an unrelated and thus far unidentified molecular machine in primates.

The loss of a highly evolved and complex resistance system that is active against vacuolar pathogens needs an adaptive explanation. The evolution of a successful avoidance strategy by the pathogens is unlikely, because many different pathogens and pathogen classes are controlled by IRG proteins in the mouse [2,4]. Nevertheless very recent evidence suggests that *Chlamydia *spp. divergence between humans and mouse may indeed be partially driven by differences in the deployment of p47 GTPases, in this case Irga6 (IIGP1) [41]. Human *Chlamydia trachomatis *is controlled by IIGP1 in interferon-treated mouse oviduct epithelial cells, whereas control of the extremely closely related mouse *C. muridarum *is independent of interferon. However, a more plausible general model is the evolution in the primate lineage of improvements to the battery of parallel mechanisms, rendering the IRG system redundant. Interestingly, in this context it was noted in the *Chlamydia *study quoted above that interferon-stimulated mouse oviduct epithelial cells did not express the important resistance factor indoleamine deoxygenase, which is responsible for tryptophan depletion, whereas this is well expressed in interferon-stimulated human HeLa cells [41]. In general, pathogen resistance mechanisms also carry costs, for example autoimmunity and allergy, arising from the adaptive immune system and many others arising from innate immunity [42-46]. Indeed, the interferon-inducible dynamin-like GTPases, the Mx proteins, which confer on mice strong resistance to certain RNA viruses and have been considered functionally related to the p47 GTPases [4,47-49], exist in a balanced polymorphism in the wild over null alleles [50,51], and have been lost spontaneously from all except two laboratory mouse strains [52]. It is not yet obvious what specific costs might be associated with possession of the Mx or IRG resistance systems.

The IRG proteins are well represented in the bony fish but, although they are abundant and diverse in the zebrafish, in *Fugu *there are only two very closely linked and similar genes that are, indeed, annotated as a single tandem gene in ENSEMBL (although we judge this not to be the case). Thus, the available annotated fish genomes seem to mirror the IRG situation in the mammals, with *Fugu *and *Tetraodon *reflecting the reduced human case and *Danio *the complex dog and mouse. However, it has not yet been reported whether any fish IRG genes respond to infection [53]. Both *Danio *and the pufferfish are Actinopterygian fish, in which where there is increasing agreement that the genome has been amplified by three rounds of duplication [54,55]. It is plausible that the complex IRG representation in *Danio *may be attributed to preservation of these genes on more than one of the potential eight paralogons, whereas only a single copy carries IRG genes in the pufferfish. Further clarification of this issue awaits the completion of the genomes.

The phylogenetic origin of the IRG proteins is obscure. Because the family is conserved at least down to the bony fishes with little structural modification, the IRG genes are not strictly fast-evolving and their basic conservatism makes them easy to identify. Thus, their apparent absence from most known invertebrate genomes is probably real. Although many components of the adaptive immune system appear to have evolved close to the chordate-vertebrate boundary [56], this is not generally the case for innate immune mechanisms [57,58] There seems to be no reason in principle why the IRG system should not work in invertebrates because it is cell-autonomous. However, the putative GTPase sequences that we have recovered from *C. elegans *and from Cyanobacteria are too distantly related outside the G-domain for a clear phylogenetic relationship to IRG proteins to be established from sequence similarity alone, whereas the similarities within the G-domains, although occasionally striking, are to some extent forced by the maintenance of a highly conserved function, namely regulated GTP hydrolysis. A stronger case for a meaningful phylogenetic relationship between these proteins and the vertebrate IRG proteins would follow from structural evidence that they display the distinctive IRG fold exemplified by mouse Irga6 (IIGP1) and from a detailed analysis of their catalytic mechanism.

The basic unit of IRG protein function may be a dimer because several genes we have identified occur in pairs in a head-to-tail arrangement, are expressed as tandem transcripts, and are presumably expressed as dimeric proteins. This conclusion is consistent with the dimer of IIGP1 (Irga6) observed in the crystal, shown by site-directed mutagenesis of the dimer interface to be essential for GTP-dependent oligomerization and cooperative hydrolysis [12]. However, a second dimer interface is also required for oligomerization (unpublished data), and which of the two dimer structures the constitutive IRG dimers represent is of considerable interest. Unlike the observed homodimer of IIGP1, the products of the two putative tandem genes in the mouse *Irgb2*/*b1 *and *Irgb5*/*b4 *are heterodimers, implying that the two IRG subunits serve distinct functions in the protein. The same may be true for the tandem pair of *irg *genes of *Fugu*, which are annotated as a single tandem gene in ENSEMBL. These latter genes have diverged very recently, because with three exceptions they are identical at the nucleotide level over the first 290 amino acids. However, they have diverged substantially in the carboxyl-terminal region (Figure 6), suggesting a recent selective force. If the two tandem IRG domains are indeed expressed as a tandem protein (as favored by the ENSEMBL annotation), then it will be as a heterodimer with significant sequence variation at the carboxyl-terminus. The extreme case of heterodimer differentiation in IRG tandem genes may occur in *Danio*, in which gene *irgg*, a canonical (although truncated) IRG gene, is apparently expressed in tandem with the adjacent downstream gene *irgq1*, which is a modified (and also truncated) quasi-GTPase gene that is unlikely to function as a GTPase (Additional data file 7). In this case the role of the irgq1 domain may be regulatory for the amino-terminal irgg domain. Other IRGQ proteins may also be regulators of IRG proteins, interacting with the functional IRG proteins with a symmetry resembling one or other of the two dimer structures. Thus IRGQ proteins would coevolve with IRG proteins. This would explain why the three *irgq *genes of the zebrafish have no homologs in the pufferfishes, with their single tandem pair of *irg *proteins, and recalls the recent observation that the GAP protein of the small GTPase, Rap1, is itself probably derived from a GTPase ancestor, retaining the G-domain structure but not the sequence to reveal its origin [59].

Better understanding of the mechanism of action and regulation of the p47 GTPases is needed before their complex evolutionary history can be put in context. From the evidence we present here, however, it is already clear that effective resistance to vacuolar pathogens in humans and mouse must be organized on radically different principles.

### Nomenclature

We introduce here a general nomenclature on phylogenetic principles for the p47 GTPases, based on the stem name IRG (immunity-related GTPases). This stem was favored over other possibilities because the name IRG-47 has priority in the literature as the first description of a p47 GTPase [60]. The phylogenetic basis of the nomenclature is apparent from Figure 5; each deep monophyletic clade is identified by a single-letter suffix as IRGM or IRGC. The nomenclature proposed here in Figure 5 and in Additional data file 1 has been accepted by the gene nomenclature committees of human and mouse, and by the zebrafish sequencing project. We have tried throughout to use the different forms of gene name accepted by the nomenclature committees for mouse (Irg) human (IRG), dog (IRG) and zebrafish (irg). The nomenclature of the *IRGQ *genes departs from the phylogenetic principle. The IRGQ nomenclature simultaneously recognizes the affinity of these sequences to *IRG *genes and stresses their anomalous GTP-binding domain features. It is, however, highly unlikely that the IRGQ sequences of humans, mouse, and fish represent a monophyletic group. It is more likely that the *IRGQ *genes of each taxonomic group derive from *IRG *genes of other clades of that group. This pattern is already apparent by inspection of the irgq protein sequences of the zebrafish in Figure 6, but it cannot be discerned from the G-domain-based phylogeny shown in Figure 5 because of the specific divergence of the G-domains of the irgq proteins.

## Materials and methods

### Use of database resources

All available public databases were extensively screened by BLAST and related searches for sequences belonging to the IRG family. In the case of the mouse, transcript sequences derived from the C57BL/6 strain were given preference over sequences of other and undefined strain origin, and compared in all cases with genomic sequence available via the ENSEMBL (v28.33d.1, February 2005) array of websites [61]. A systematic study of polymorphism has not yet been completed, but it is already clear that nearly all IRG sequences derived from the CZECHII cDNA libraries (*Mus musculus musculus*) differ from C57BL/6 sequences. These differences make allocation of many CZECHII sequences to individual clade members of the C57BL/6 mouse problematic. Identification of certain *Irg *sequences with recognized gene symbols was achieved through the Mouse Genome Initiative web resources [62]. Where ambiguities persist in the mouse genomic map, especially on chromosome 18 in the region of *IrgA6*-*IrgA8 *(Mb60.878-60.958), and on chromosome 11 in the region from *PA28βψ *to *IrgB7ψ *(Mb57.570-57.700), we used primary BAC and cosmid sequences to reach a consensus view.

Human and dog IRG sequences were identified from the available public databases (ENSEMBL, National Center for Biotechnology Information) and confirmed wherever possible by multiple sequence comparisons at transcriptional and genomic levels. *Fugu *material was obtained and analyzed through [63-65]. Tetraodon sequence was initially assembled from the GSS sequence database at National Center for Biotechnology Information and subsequently from the University of California at Santa Cruz compiled genome database [66] via the BLAST server. Zebrafish sequence was obtained from zebrafish genome resources at the Sanger Centre [67] and analyzed in an Acedb database using the Spandit annotation tool.

Chromosomal locations and synteny analysis of mouse and human chromosomes was initiated through ENSEMBL [68]. Further details were obtained through the Sanger Centre [69]. Nucleotide sequences and translated open reading frames of IRG family members used in this paper are given in Additional data files 9 and 10, and can also be accessed at the IRG family database at our laboratory [14].

### Phylogeny and alignment protocols

Routine sequence analysis and local sequence database management was handled using DNA-Strider 1.3f12, Vector-Nti, and MacVector 7.2. The identity and similarity matrix on protein and nucleotide sequences (Additional data file 2) are based on GeneDoc (version number 2.6.002). Phylogenetic analysis was conducted using the neighbor-joining method [70], as implemented in the MEGA2 program [71]. We used p-distances for constructing the phylogenetic trees. Reliability of the neighbor-joining trees was examined using the bootstrap test [72].

Alignments were performed via the BCM multiple alignment programme suite [73] and EBI-ClustalW [74] using the default options and manipulated according to the crystal structure of IIGP1 [12]. Shading of alignments was performed using Boxshade [75] and additional sequences were shaded manually according to the default options of Boxshade.

### Identification of transcription factor binding sites

Promoter regions (2 kb upstream of putative transcription start point) were screened for putative transcription factor binding sites with the Transcription Element Search System [76,77], and the results were further analysed and confirmed manually. Additional promoter analysis of Irgc (mouse Cinema) and IRGC (human CINEMA) was performed with ConSite [78] based on phylogenetic footprinting [79].

### RT-PCR on cells and tissues

C57BL/6J mice were obtained from the animal house at the Institute for Genetics, University of Cologne. *Listeria monocytogenes *infection was performed as described previously [13]. Twenty-four hours after infection, the mice were killed, and liver, lung and spleen were removed and snap frozen in liquid nitrogen. Mouse L929 fibroblasts were stimulated for 24 hours with 200 U/ml interferon-γ or 200 U/ml interferon-β (R&D System GmBH, Weisbaden-Nordenstadt, Germany and Calbiochem-Novabiochem Corparation La Jolla, CA, respectively). Human cell lines (Hela, GS293 (GeneSwitch™ -293, Invitrogen GmbH Karlsruhe, Germany), HepG2, T2, THP1, MCF-7, SW-480, Primary foreskin fibroblast-HS27) were stimulated for 24 hours with 2,000 U/ml interferon-β or 200 U/ml interferon-γ (PBL Biomedical Laboratories, NY, USA and Peprotech/Cell concepts GmbH UmKirsh, Germany, respectively). Total RNA was extracted from tissues and cells using the RNAeasy mini kit (QIAGEN, Hilden, Germany), except for testis, for which the RNAeasy Lipid Tissue Kit (QIAGEN) was used. Poly(A) RNA was isolated from total RNA using the Oligotex mRNA kit (QIAGEN). Total RNA from human tissues was purchased from Biochain (Hayward, CA, USA). cDNA was generated from mRNA and total RNA using the Super Script First-Strand Synthesis System for RT-PCR (Invitrogen, Carlsbad, CA, USA). The generated cDNAs were screened for the presence of p47 (IRG) GTPase transcripts by PCR. A list of the primers used is given in Additional data file 8. The amplified fragments were confirmed by sequencing.

## Additional data files

The following additional data are included with the online version of this paper: A list of all IRG gene family members described in this paper (gives names, synonyms, accession numbers and further information for each IRG gene; Additional data file 1); nucleotide and amino acid identities based on G-domain of mouse Irg family (gives percentage of identity on both protein and nucleotide level within the mouse Irg family; Additional data file 2); ISRE and GAS elements of mouse IRG family genes (contains the positions and exact sequences of all ISRE and GAS elements found in putative promoters of mouse IRG genes; Additional data file 3); inducibility of Dog p47 (IRG) GTPases (shows interferon inducibility of members of the p47 (IRG) GTPases present in the dog; Additional data file 4); genomic organization of *Danio rerio *p47 (irg) GTPases (illustrates the genomic organization of all p47 (irg) GTPases found in zebrafish to date; Additional data file 5); protein similarity matrix of Irgc and Irgq (contains comparison between the mouse p47 GTPase Irgc and the long coding exon of the closely linked quasi-GTPase Irgq (FKSG27); Additional data file 6); divergent nucleotide-binding motifs in quasi-GTPases (compares the nucleotide binding motifs of quasi-GTPases to those of the classical mouse p47 GTPases; Additional data file 7); a list of the primers used (contains the sequences of all primers used in this study; Additional data file 8); nucleotide sequences of all IRG family members (Additional data file 9); protein sequences of all IRG family members (Additional data file 10).

## Supplementary Material

Additional data file 1 A list of all IRG gene family members described in this paper (gives names, synonyms, accession numbers and further information for each IRG gene) Click here for file

Additional data file 2 Nucleotide and amino acid identities based on G-domain of mouse Irg family (gives percentage of identity on both protein and nucleotide level within the mouse IRG family) Click here for file

Additional data file 3 ISRE and GAS elements of mouse IRG family genes (contains the positions and exact sequences of all ISRE and GAS elements found in putative promoters of mouse IRG genes) Click here for file

Additional data file 4 Inducibility of Dog p47 (IRG) GTPases (shows interferon inducibility of members of the p47 (IRG) GTPases present in the dog) Click here for file

Additional data file 5 Genomic organization of *Danio rerio *p47 (IRG) GTPases (illustrates the genomic organization of all p47 (IRG) GTPases found in zebrafish to date) Click here for file

Additional data file 6 Protein similarity matrix of Irgc and Irgq (contains comparison between the mouse p47 GTPase Irgc and the long coding exon of the closely linked quasi-GTPase Irgq (FKSG27) Click here for file

Additional data file 7 Divergent nucleotide-binding motifs in quasi-GTPases (compares the nucleotide binding motifs of quasi-GTPases to those of the classical mouse p47 GTPases) Click here for file

Additional data file 8 A list of the primers used (contains the sequences of all primers used in this study) Click here for file

Additional data file 9 Nucleotide sequences of all IRG family members Click here for file

Additional data file 10 Protein sequences of all IRG family members Click here for file
