# Supplementary material for: The interferon-inducible p47 (IRG) GTPases in vertebrates: loss of the cell autonomous resistance mechanism in the human lineage
Source: Genome Biol. 2005 Oct 31;6(11):R92. doi: 10.1186/gb-2005-6-11-r92 (PMC1297648; doi:10.1186/gb-2005-6-11-r92)
Supplement: Additional data file 1 — A list of all IRG gene family members described in this paper (gives names, synonyms, accession numbers and further information for each IRG gene) [file gb-2005-6-11-r92-S1.pdf]

## Additional Data File 1: List of all IRG gene family members and related genes described in Bekpen *et al*. 2005

| Gene name | Genesymbol/ID | Synonyms | Genomic sequences /Accession no. | cDNA or EST sequence Accession numbers | Notes |
|---|---|---|---|---|---|
| Mouse | | | | | |
| *Irga1* | *Irga1*<br>MGI:1795294<br>MGI:1653512 | | AC132320<br>AC102225 | BI658674 (NMRI, 5'EST, nearly 100%)<br>BG915086 (NMRI, 5'EST; not 100% ) | |
| *Irga2* | *Irga2*<br>MGI:915200<br>MGI:1257137<br>MGI:1257136 | | AC132320<br>AC102225<br>XM_140378 | AA968296 (C57BL/6, 5'EST, 100%, not full length)<br>AA968378 (C57BL/6, 3'EST, 100%, not full length) | Inducible by IFN-γ. |
| *Irga3* | *Irga3*<br>New gene | | AC132320<br>XM_140379 (C57BL/6J) | BY751179 (NOD, EST, not 100%, 610bp) | Inducible by IFN-γ. |
| *Irga4* | *Irga4*<br>New gene | | AC132320)<br>XM_140380 (*Irgb4/Irgb5* tandem) | BY750970 (NOD, EST, nearly 100%, 700 bp)<br>BU696309 (C57BL/6, EST, nearly 100%, 530 bp) | Inducible by IFN-γ. |
| *Irga5 Ψ* | *Irga5*<br>New gene | | AC132320 | None | A transcript is inducible by IFN-γ but the coding sequence of the gene is disrupted repeatedly. |
| *Irga6* | *Irga6*<br>MGI:1926259<br>MGI:2147195<br>MGI:2147350 | IIGP, IIGP1, Iigp1 | AC135638 | AJ007971 (C57BL/6, 100% correct)<br>AF194871 (C57BL/6, also NM_021792, 100%)<br>BC004649 (C57BL/6, cDNA 100%, 2330bp) | [13] (MGI:1889878); [16]<br>Inducible by IFN-γ. |
| *Irga7* | *Irga7*<br>New gene | | NT_039674 (C57BL/6J, Chr.18 genomic contig, 73.9 Mb)<br>XM_487533 (C57BL/6, 100%) | None known | |
| *Irga8* | *Irga8*<br>MGI:953940<br>(C57BL/6)<br><br>MGI:2384767<br>MGI:1489193<br>(CZECHII) | MGC:28198<br>BC023105 | AC135638 | BC023105 (CZECHII cDNA, = NM_145357, not 100%, full length)<br>BB637466 (C57BL/6J, 5'EST, not 100%, not full length)<br>BF163606 (CZECHII, not 100%, not full length)<br>BE198503 (C57BL/6, 3'EST, 100%, not full length)<br>BE198089 (C57BL/6, 3'EST, 100%, not full length)<br>BX520309 (C57BL/6, 3'EST, 100%, not full length | In C57BL/6 a non-canonical guanine after bp 849 in BC023105 (= aa 204) puts the sequence out of frame just before Helix H4; the reading frame is complete in BC023105 (CZECHII, *Mus musculus musculus*).<br>Inducible by IFN-γ. |
| *Irgb1* | *Irgb1*<br>MGI:1519766 | | AL645849 | BC022776 tandem *Irgb2/ Irgb1* (CZECHII, not 100%, protein: Q8R5D8)<br>BF144722 (CZECHII, EST, not 100%, starts with 3' end of *Irgb2*) | The *Irgb2/Irgb1* gene pair is almost certainly transcribed in tandem. The protein has not yet been described.<br>Inducible by IFN-γ. |
| *Irgb2* | *Irgb2* | | AL645849 | BC022776 tandem *Irgb2/ Irgb1* (CZECHII, not | See note above, *Irgb1*. |

| | | | | | |
|---|---|---|---|---|---|
| | MGI:1518599 | | | 100%, protein: Q8R5D8)<br>BF144934 (CZECHII, 5' *Irgb2* cDNA, not 100%)<br>BY735436 (from cell line RCB-0527 Jyg-MC(B), strain unknown, 5' *Irgb2*, not 100%) | |
| *Irgb3* | *Irgb3*<br>MGI:1553791<br>(FVB/N) | | AL627237<br>AL669850 (unordered)<br>AF060196 (129/SvJ, genomic, 1 bp difference, ATG*(Irgb3)*= bp 1353; Stop = bp 2659) | BF539106 (FVB/N, 3'EST, not 100%) | The genomic sequence of *Irgb3* is followed after 950 bp by a retroposon corresponding to the proteasome regulator PA28b [80] (MGI:1331589). The presence or absence of this retroposon unambiguously distinguishes *Irgb3* from *Irgb4.* |
| *Irgb4* | *Irgb4*<br>MGI:1795392<br>MGI:3041173 | 9930111J21Rik | AL627237<br>AL669850 (unordered) | BC066104 (C57BL/6, *Irgb5/Irgb4* tandem, 100%)<br>BI655221 (NMRI, EST, not 100%) | See note above for *Irgb3*.<br>*Irgb4* is probably normally expressed as a distinct 3' exon in a tandem transcript downstream of *Irgb5*. |
| *Irgb5* | *Irgb5*<br>MGI:3041173<br>MGI:2401562 | 9930111J21Rik | AL627237<br>AL645688<br>AL669850 (unordered) | BC066104 (C57BL/6, *Irgb5/Irgb4* tandem; not 100% at 5' end)<br>AK037088 (C57BL/6, cDNA, = NM_173434, 100%, unknown 5' end) (protein = BAC29698= Q8CB10) | *Irgb5* is probably normally expressed as a separate 5' exon in a tandem transcript upstream of *Irgb4.* However AK037088 does not splice into *Irgb4*. Thus *Irgb5* can exist as a single p47 unit or as a tandem with *Irgb4.*<br>The reference number MGI:2401562 refers to several ESTs belonging to *Irgb5* and *Irgb9*.<br>Inducible by IFN-γ. |
| *Irgb6* | *Irgb6*<br>MGI:98734<br>MGD-MRK-15077 | TGTP,<br>Mg21, Gtp2 | AL627237<br>AL645688<br>AL669850 (unordered) | L38444 (C57BL/6, 100%)<br>NM_011579 (NOD, 2 aa difference)<br>U15636 (C.D2-Idh-1/Pep-3, 2 aa difference)<br>BC085259 (NMRI, cDNA, 100%)<br>BC034256 (CECHII, cDNA, not 100%) | [81, 82]<br>Inducible by IFN-γ. |
| *Irgb7Ψ* | *Irgb7*<br>New gene | | AL645688<br>AL669850 (unordered) | None known | Pseudogene: STOP codon before G-domain. Not inducible by IFN-γ, no known transcript. |
| *Irgb8* | *Irgb8*<br>MGI:1672892 | | AL645849 | BG974191 (NMRI, 3' EST, not full length, not 100%,) | So similar to *Irgb1*, *b3* and *b4* that non-identical EST sequences are hard to disentangle. |
| *Irgb9* | *Irgb9*<br>MGI:2401562<br>New gene | | AL645849<br>XM_204704 (C57BL/6, full length, 100%) | BB630182 (EST, short) | The reference number MGI:2401562 refers to several ESTs belonging to *Irgb5* and *Irgb9*. |
| *Irgb10* | *Irgb10*<br>MGI:1282384 | | AL928857 | AI122314 (C57BL/6, short EST, not 100%) | Short, terminates before end of G domain in S6.<br>Inducible by IFN-γ. |
| *Irgc* | *Irgc*<br>New gene | CINEMA | AC073810 (RP23-57J6)<br>GENSCAN00000140134 | BB615720 (C57BL/6 cDNA, 99%, 606 bp)<br>36 ESTs, none full length (e.g. CA464745 5'mRNA, 874bp, 100% except of first two bp) | An *Irgc*-related sequence has recently been named HGTP-47 [4]. This sequence (NP_950178=NM_199013= AK089224, NOD) contains 4 frameshifts relative to the C57BL/6 genomic sequence leading to a largely incorrect protein sequence. The reference numbers MGI:2685948 and MGI:2685320 both relate to this |

| | | | | | |
|---|---|---|---|---|---|
| | | | | | error sequence. |
| *Irgd* | *Irgd*<br>MGI:99448<br>MGD-MRK-16217 | IRG-47, IRG47, Ifi47, 47kDa, Iigp4 | AL645688<br>AL669850 (unordered) | M63630 (B6D2F1, =NM_008330, 100% correct) | [60]. This is the first report of a p47 GTPase and has given its name (IRG-47) to the whole family.<br>Inducible by IFN-γ. |
| *Irgm1* | *Irgm1*<br>MGI:107567<br>MGD-MRK-36139 | LRG-47, LRG47, Ifi1, Iigp3 | AL645849 | U19119 (BALB/c, =NM_008326, 100% correct) | [83]; Two 5' splice variants exist.<br>See notes human IRGM below.<br>Inducible by IFN-γ. |
| *Irgm2* | *Irgm2*<br>MGI:1926262<br>MGI:2144195 | GTPI<br>Iigp2 | AL928857 | AJ007972 (C57BL/6; 100%)<br>NM_019440 (CZECHII, = BC005419, not 100%) | [13], MGI:1889878.<br>Two 5' splice variants exist.<br>Inducible by IFN-γ. |
| *Irgm3* | *Irgm3*<br>MGI:107729<br>MGD-MRK-36305<br>MGI:2144580 | IGTP<br>Igtp | AL928857 | U53219 (C57BL/6, cDNA, 100%)<br>NM_018738 (NOD, cDNA, not 100%) | [84], MGI:82341<br>Inducible by IFN-γ. |
| *Irgq* | *Irgq*<br>MGI:2667176 | FKSG27 | AC073810 | AF322649 (C57BL/6, mRNA, = NM_153134) | |
| Human | | | | | |
| *IRGC* | UniGene<br>Hs.515444<br>R30953_1<br>GeneID: 56269 | CINEMA<br>human IIGP5, cinema1 | AC005622<br>HChr.19 cosmid | BC066939 (cDNA, 100%)<br>NM_019612 (cDNA, 100%) | |
| *IRGM* | UniGene<br>Hs.519680<br>GeneID: 345611<br>MIM: 608212 | human LRG-47-like protein (LRG47, LRG-47), IFI1 | AC010441<br>Chr.5<br>XM_293893 (splice variant a, 100%) | BC038360 (splice variant c, 3'EST)<br>BC038539 (short EST)<br>BI764111 (short EST)<br>Sequences have been confirmed by RT-PCR (unpublished) | 5 different 3' splice variants (a-e) (see main paper Bekpen *et al*, Fig. 8b).<br>The orthology of *Irgm1* with human *IRGM* implied by use of the name *LRG47* or IFI1 for the human gene is incorrect. The use of LRG47 as a synonym or alias for human IRGM is therefore not recommended. |
| *IRGQ* | UniGene<br>Hs.546476<br>GeneID: 126298 | Homo sapiens FKSG27, Irgq1 | AC006276 | AF322648 (=NM_001007561 mRNA, 100%) | |
| Dog | | | | | |
| *IRGB11* | New gene | | AACN010148430<br>AAEX1030324<br>AAEX1030325 | | |
| *IRGB12* | New gene | | AACN01030937<br>AAEX1030324<br>AAEX1030325 | | Confirmed by RT-PCR but not sequenced.<br>Inducible by IFN-γ. |
| *IRGC* | New gene | CINEMA | AACN010031536 | | |

| | | | | | |
|---|---|---|---|---|---|
| | | | AAEX01054272 | | |
| *IRGD* | New gene | | AAEX01030325 | | |
| *IRGM4* | New gene | | AAEX01059458 | | Confirmed by RT-PCR but not sequenced. Inducible by IFN-γ. |
| *IRGM5* | New gene | | AACN010384735<br>AAEX01030325 | | Confirmed by RT-PCR but not sequenced. Inducible by IFN-γ. |
| *IRGM6* | New gene | | AACN010300899<br>AAEX1030325 | | Confirmed by RT-PCR but not sequenced. Inducible by IFN-γ. |
| Fugu | | | | | |
| *irgf5* | | | Fugu_Sc2554 (Ensembl v3) | | *irgf* genes of zebrafish, Fugu and Tetraodon have the long coding exon broken by an intron. |
| *irgf6* | | | Fugu_Sc2554 (Ensembl v3) | CA589084 (GI:25133662: 606 bp mRNA linear EST; hab53f04.y1 Fugu UT7 adult skin Takifugu rubripes cDNA clone)<br>AL837863 (GI:21879801; 491 bp mRNA linear; F000A Takifugu rubripes cDNA clone F000A03aF7, mRNA sequence, skin) | See note above, *irgf5* |
| Tetraodon | | | | | |
| *irgf7* | | | SCAF112 (Ensembl v32, Jul 05) | GSTENT00000024001 | *irgf* genes of zebrafish, Fugu and Tetraodon have the long coding exon broken by an intron. |
| *irgf8* | | | SCAF112 (Ensembl v32, Jul 05) | GSTENT00000023001 | See note above, *irgf7*. |
| Zebrafish | | | | | |
| *irge1* | XP_693404 | | AL935330 (CH211-230C14)<br>CR391937 (CH211-175G6) | BM316215 (3' EST) | Zebrafish *irge* genes have the long coding exon unbroken by an intron, like the mammalian p47 genes<br>XP_693404 (GI:68383735, 502 aa linear VRT 30-JUN-2005 predicted: similar to immunity-related GTPase family, cinema 1 [Danio rerio]. DBSOURCE REFSEQ: accession XM_688312.1 (Short N-terminus) |
| *irge2* | XP_693474 | | AL935330 (CH211-230C14)<br>CR391937 (CH211-175G6) | None | See note above, *Irge1*.<br>XP_693474 (GI:68383738, 352 aa linear VRT 30-JUN-2005 predicted: similar to immunity-related GTPase family, cinema 1 [Danio rerio]. DBSOURCE REFSEQ: accession XM_688382.1 (Short N-terminus) |
| *irge3* | | | AL935330 (CH211-230C14)<br>CR391937 (CH211-175G6) | AW233145 (5' cDNA ) | See note above, *Irge1*.. |
| *irge4* | XP_693622 | | AL935330 (CH211-230C14)<br>CR391937 (CH211-175G6) | CN501017 (5' EST)<br>CK142408 (5' EST) | See note above, *Irge1*..<br>XP_693622 (GI:68383741, 385 aa linear VRT 30- |

| | | | | | |
|---|---|---|---|---|---|
| | | | | | JUN-2005 predicted: similar to immunity-related GTPase family, cinema 1 [Danio rerio]. DBSOURCE REFSEQ: accession XM_688530.1 |
| *Irge5* | XM_681093 | | NW_635044 (GI:67045019; chr. 9 contig; bp 307225 308757) | | See note above, *Irge1*. XM_681093 (GI:68365895, 1533 bp mRNA linear VRT 30-JUN-2005 predicted: Danio rerio similar to immunity-related GTPase family, cinema 1 (LOC557936), mRNA. |
| *Irge6* | XM_695163 | | NW_633868 (gi:67045754; chr. 18 contig; bp 5057602-5058696) | | See note above, *Irge1*. XM_695163 (GI:68390584, 1095 bp mRNA linear VRT 30-JUN-2005 predicted: Danio rerio similar to immunity-related GTPase family, cinema 1 (LOC571560), mRNA. |
| *irgf1* | XP_700498 | | CR384077<br>DKEY-79I2 | CN503005 (5' EST) | *irgf* genes of zebrafish, Fugu and Tetraodon have the long coding exon broken by an intron. XP_700498 (397 aa linear VRT 30-JUN-2005 predicted: similar to immunity-related GTPase family, cinema 1, partial [Danio rerio]. |
| *irgf2* | | | CR384077<br>DKEY-79I2 | None | See note above, *irgf1*. |
| *irgf3* | | | WGS traces<br>zDH64-1061h13.q1k<br>ZDH88-124d21.p1k<br>zfish35935-195b06.p1c | AL924569 | See note above, *irgf1*. |
| *irgf4* | | | ENSDARG00000010545 | None | See note above, *irgf1*. |
| *irgg* | | | AL935330 (CH211-230C14)<br>CR391937 (CH211-175G6) | CA473205 (5' EST) | No intron in long coding exon. Short, terminates in Helix F.<br>Probably the 5' end of a tandem with *irgq1*. |
| *irgq1* | | | AL935330 (CH211-230C14)<br>CR391937 (CH211-175G6) | BQ481364 (5' EST) and BQ481122 (3' EST) from cDNA clone IMAGE:5899497. The 5' end of this clone is in the 3' end of *irgg* and reads into the 5' end of *irgq1*. | Short, terminates in helix F. Probably the 3' end of a tandem with *irgg.* |
| *irgq2* | XP_684591 | | BX072550<br>DKEY-245P1 | BF938149 5' EST and BI880124 3'EST from cDNA clone IMAGE:4200886 | XP_684591 (GI:68381188, 379 aa linear VRT 30-JUN-2005 predicted: similar to RGD1311107_predicted protein [Danio rerio]. DBSOURCE REFSEQ: accession XM_679499.1 |
| *irgq3* | | | BX127973;<br>SP6 end of BAC DKEY-279M7<br>Zv4_scaffold1709.9 | None | |
| *C. elegans* | | | | | |
| C46E1.3 | WP:CE34758<br>GI:3300129; | | AL008867.1 (GI:3217208, cosmid C46E1) | None | Predicted protein, tandem G domains. |

| | | | | | |
|---|---|---|---|---|---|
| | CAE17750 | | | | |
| W09C5.2 | CAB63329.1 GI:6580259 | | Z82077 (GI:3873420, Cosmid W09C5) | None | Predicted protein. |
| Bacteria | | | | | |
| BAA10832 | GI:1001345 | | BA000022.2 (GI:47118304, Synechocystis sp. PCC 6803) | | Synechocystis sp . PCC 6803 Predicted protein. |
| BAA18140 | GI:1653224 | | BA000022.2 (GI:47118304, Synechocystis sp. PCC 6803) | | Synechocystis sp . PCC 6803 Predicted protein. |
| BAA18642 | GI:1653731 | | BA000022.2 (GI:47118304, Synechocystis sp. PCC 6803) | | Synechocystis sp . PCC 6803 Predicted protein. |
| BAC08557 | GI:22294728 | | BA000039.2 (GI:47118315, T. elongatus BP-1) | | Thermosynechococcus elongatus BP-1 Predicted protein. |
| BAC08842 | GI:22295014 | | BA000039.2 (GI:47118315, T. elongatus BP-1) | | Thermosynechococcus elongatus BP-1 Predicted protein. |
