# Supplementary material for: The interferon-inducible p47 (IRG) GTPases in vertebrates: loss of the cell autonomous resistance mechanism in the human lineage
Source: Genome Biol. 2005 Oct 31;6(11):R92. doi: 10.1186/gb-2005-6-11-r92 (PMC1297648; doi:10.1186/gb-2005-6-11-r92)
Supplement: Additional data file 2 — Nucleotide and amino acid identities based on G-domain of mouse Irg family (gives percentage of identity on both protein and nucleotide level within the mouse IRG family) [file gb-2005-6-11-r92-S2.pdf]

Irg	m1	m3	m2	b3	b4	b8	b1	b6	b2	b7	b5	b9	b19	a2	a6	a1	a5	a3	a8	a4	a7	d	c
m1		0.71	0.75	0.53	0.53	0.52	0.51	0.53	0.45	0.46	0.48	0.49	0.42	0.47	0.48	0.48	0.47	0.46	0.45	0.46	0.47	0.50	0.51
m3	0.64		0.80	0.49	0.49	0.48	0.49	0.49	0.44	0.45	0.47	0.47	0.40	0.47	0.48	0.48	0.45	0.46	0.46	0.46	0.46	0.47	0.46
m2	0.65	0.77		0.53	0.52	0.53	0.53	0.53	0.46	0.47	0.49	0.50	0.41	0.50	0.51	0.51	0.48	0.48	0.49	0.49	0.49	0.50	0.49
b3	0.40	0.38	0.40		0.99	0.96	0.94	0.80	0.68	0.69	0.74	0.73	0.53	0.64	0.64	0.65	0.60	0.62	0.63	0.63	0.62	0.61	0.55
b4	0.40	0.37	0.40	0.99		0.96	0.94	0.80	0.68	0.69	0.74	0.73	0.53	0.64	0.64	0.65	0.60	0.62	0.62	0.63	0.62	0.61	0.55
b8	0.40	0.37	0.39	0.95	0.95		0.95	0.81	0.68	0.69	0.73	0.73	0.53	0.65	0.65	0.66	0.61	0.63	0.63	0.64	0.62	0.61	0.55
b1	0.40	0.37	0.41	0.89	0.89	0.91		0.81	0.66	0.68	0.72	0.71	0.52	0.64	0.64	0.65	0.61	0.62	0.63	0.65	0.63	0.61	0.54
b6	0.41	0.34	0.39	0.73	0.73	0.75	0.75		0.65	0.66	0.69	0.69	0.53	0.64	0.64	0.66	0.59	0.62	0.62	0.63	0.63	0.63	0.56
b2	0.34	0.31	0.33	0.55	0.55	0.57	0.52	0.52		0.85	0.82	0.80	0.45	0.57	0.57	0.57	0.54	0.56	0.57	0.56	0.56	0.52	0.46
b7											0.83	0.82	0.47	0.58	0.57	0.58	0.56	0.58	0.58	0.59	0.58	0.54	0.47
b5	0.35	0.33	0.36	0.59	0.59	0.60	0.56	0.54	0.82			0.96	0.50	0.62	0.61	0.62	0.59	0.62	0.62	0.61	0.60	0.57	0.53
b9	0.35	0.33	0.35	0.57	0.57	0.58	0.54	0.54	0.80		0.94		0.51	0.61	0.61	0.62	0.59	0.62	0.62	0.62	0.60	0.57	0.52
b10	0.33	0.31	0.31	0.47	0.47	0.48	0.47	0.49	0.39		0.40	0.40		0.50	0.49	0.50	0.47	0.48	0.48	0.50	0.49	0.49	0.43
a2	0.37	0.34	0.35	0.54	0.54	0.55	0.55	0.57	0.47		0.48	0.48	0.44		0.89	0.90	0.69	0.70	0.71	0.70	0.70	0.61	0.51
a6	0.36	0.34	0.35	0.57	0.56	0.57	0.57	0.59	0.47		0.49	0.50	0.44	0.84		0.92	0.70	0.69	0.70	0.70	0.70	0.61	0.52
a1	0.38	0.37	0.37	0.56	0.57	0.56	0.56	0.60	0.47		0.50	0.50	0.43	0.85	0.90		0.70	0.69	0.70	0.71	0.71	0.62	0.51
a5																		0.68	0.68	0.68	0.67	0.57	0.49
a3	0.34	0.33	0.34	0.51	0.51	0.52	0.50	0.53	0.48		0.51	0.51	0.39	0.56	0.54	0.55			0.95	0.80	0.80	0.59	0.52
a8	0.35	0.33	0.35	0.54	0.54	0.54	0.54	0.55	0.47		0.50	0.50	0.39	0.57	0.56	0.58		0.90		0.81	0.81	0.59	0.51
a4	0.35	0.35	0.35	0.56	0.56	0.56	0.56	0.54	0.46		0.49	0.49	0.43	0.58	0.59	0.60		0.68	0.69		0.90	0.62	0.51
a7	0.36	0.34	0.36	0.55	0.55	0.55	0.55	0.56	0.45		0.49	0.48	0.42	0.59	0.59	0.61		0.69	0.71	0.84		0.60	0.49
d	0.37	0.35	0.38	0.52	0.52	0.53	0.53	0.55	0.40		0.44	0.44	0.44	0.52	0.52	0.53		0.48	0.49	0.50	0.49		0.56
c	0.35	0.36	0.35	0.46	0.46	0.45	0.44	0.45	0.37		0.39	0.39	0.36	0.44	0.45	0.44		0.45	0.45	0.44	0.43	0.50	

Additional Data File 2: Nucleotide and amino acid identities based on G-Domain of mouse Irg family . Identity matrix of pairwise aligned nucleotide (gray background) or amino acid (white background) sequences of mouse Irg family members.
