# Supplementary material for: The interferon-inducible p47 (IRG) GTPases in vertebrates: loss of the cell autonomous resistance mechanism in the human lineage
Source: Genome Biol. 2005 Oct 31;6(11):R92. doi: 10.1186/gb-2005-6-11-r92 (PMC1297648; doi:10.1186/gb-2005-6-11-r92)
Supplement: Additional data file 3 — ISRE and GAS elements of mouse IRG family genes (contains the positions and exact sequences of all ISRE and GAS elements found in putative promoters of mouse IRG genes) [file gb-2005-6-11-r92-S3.pdf]

Gene name	Distance	GAS	Distance	ISRE
Irga1	-133	GTTCTTGGAA	-148	AGTTTCACTTTCCT(+)
	-188	CTTCTTTGAA		
Irga2	-119	GTTCTTGGAA	-134	AGTTTCACTTTCCT(+)
	-174	CTTCTTTGAA		
Irga3	-142	CTTCTTTGAA	-156	TGTTTCACTTTCAT(+)
	-207	TTTCTGCCAA		
Irga4	-141	CTTCTTTGAA	-156	TGTTTCACTTTCAT(+)
	-184	GTTTCTGGAA		
Irga6(p1)*	-162	CTTCTTTGAA	-176	TGTTTCACTTTCAT(+)
	-226	TTTCTTGCAA	-235	CCTTTCTCTTTCTG(+)
	-312	GTTCCATTAA		
Irga6(p2)*	-170	CTTCTTAGAA	-130	AGTTTCACTTTCCT(+)
Irga8	-30	CTTCTTTGAA	-45	GGTTTCACTTTCAT(+)
	-93	TTTCTGCCAA		
Irgb2	-87	TTTCCAGGAA	-77	AGAAAGTGAAACCT
Irgb4			-381	AGAAAGAGAAAGAC
			-627	TCAAAGAGAAAGTT
Irgb6	-96	TTTCCAGGAA	-86	CGAAACCGAAACCT
Irgb9			-223	AGAAAGAGAAAGAA
			-562	TCAAAGAGAAAGTT
			-665	TCAAAGAGAAAGAC
Irgb10	-61	ATTACTGGAA	-47	ACTTTCAGTTTCAC(+)
			-93	GCTTTCAGTTTCT (+)
Irgd(p1)*	-821	TTTCTGTGAA	-301	ACTTTCTCTTTGAA(+)
Irgd(p2)*	-21	TTTCCTGCAA	-35	AGTTTCACTTTTGT(+)
	-80	TTTCCTGGAA		
Irgm1	-64	TTTCAAGAAA	-54	AGAAACCGAAACTG
	-1061	TTTCCGGTAA	-1050	AGAAAGAGAAAGCC
Irgm2	-95	TTTCCAGGAA	-85	TGAAACTGAAAGCT
Irgm3	-108	TTTCTAGGAA	-98	TGAAACTGAAAGCT
			-825	TGAAAATGAAAGAC

Additional Data File 3: ISRE (Interferon stimulated Response Element) and GAS (Gamma activated sequences) elements of mouse Irg family genes.

Values in the Distance column denote the position of ISRE and GAS element relative to the putative transcription start site. Black and gray shading indicates optimal and suboptimal binding site respectively. *(p1) alternative upstream promoter, (p2) alternative downstream promoter. ISRE and GAS elements marked as (+) have the same orientation relative to the putative transcription start site.
