# Supplementary material for: The interferon-inducible p47 (IRG) GTPases in vertebrates: loss of the cell autonomous resistance mechanism in the human lineage
Source: Genome Biol. 2005 Oct 31;6(11):R92. doi: 10.1186/gb-2005-6-11-r92 (PMC1297648; doi:10.1186/gb-2005-6-11-r92)
Supplement: Additional data file 4 — Inducibility of Dog p47 (IRG) GTPases (shows interferon inducibility of members of the p47 (IRG) GTPases present in the dog) [file gb-2005-6-11-r92-S4.pdf]

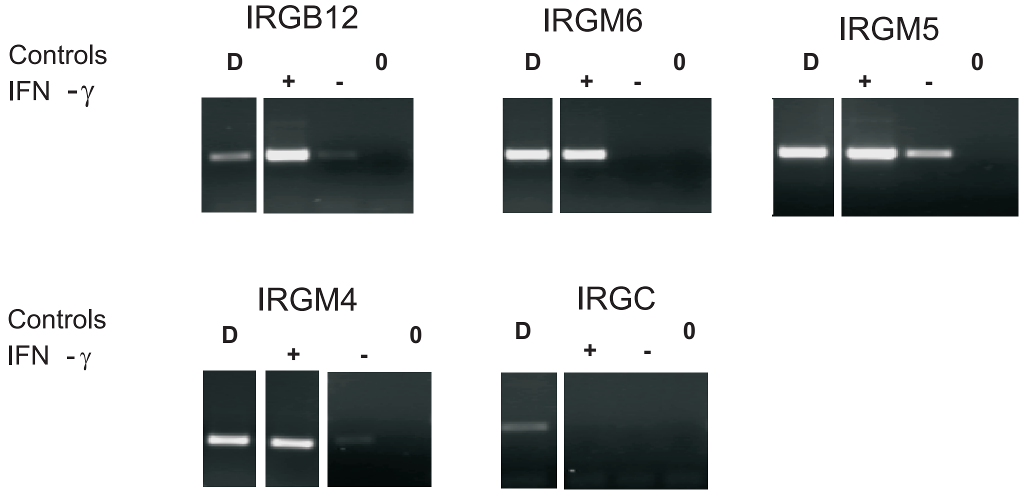


**Additional Data File 4: Inducibility of Dog GTPases**

MDCK II cells were induced (+) or not induced (-) with 10 ng/ml dog interferon-γ for 24 hours, D refers to 30 ng of genomic DNA as positive control, 0 refers to no DNA as negative control.
