# Supplementary material for: The interferon-inducible p47 (IRG) GTPases in vertebrates: loss of the cell autonomous resistance mechanism in the human lineage
Source: Genome Biol. 2005 Oct 31;6(11):R92. doi: 10.1186/gb-2005-6-11-r92 (PMC1297648; doi:10.1186/gb-2005-6-11-r92)
Supplement: Additional data file 5 — Genomic organization of Danio rerio p47 (IRG) GTPases (illustrates the genomic organization of all p47 (IRG) GTPases found in zebrafish to date) [file gb-2005-6-11-r92-S5.pdf]

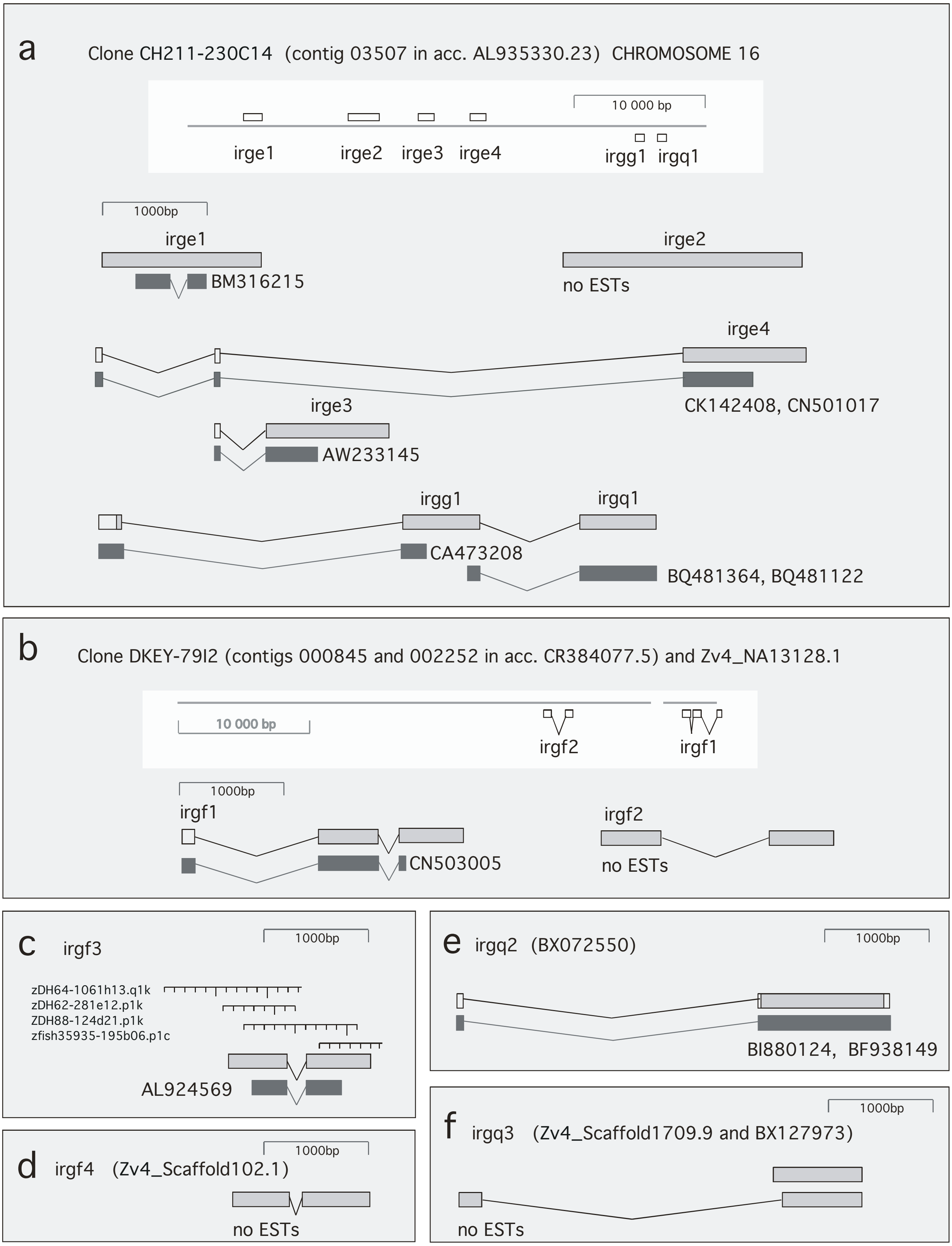

Additional Data File 5: Genomic organisation of the *Danio rerio* p47 GTPases

Predicted exons are shown as boxes in light grey (coding regions) and white (untranslated regions); gene predictions are based on splice consensus sites, coding probability and supporting ESTs and cDNAS, where available. Dark grey boxes show the supporting cDNA and EST sequences (accession numbers next to the diagrammes) that correspond to the predicted exons. All gene structures are shown at the same scale, as indicated; the two contigs with multiple ORFs are shown at lower resolution, as indicated.

a. Six ORFs encoding p47 GTPases in clone CH211-230C14 (top) of which four are supported by cDNAs. BM 316215 is probably derived from a transcript from the opposite strand. There are no splice consensus sites on the coding strand.
b. Two ORFs in clone DKEY-79I2 (top) of which one is supported by cDNAs. The 5' end of irgf2 is missing in CR384077.5 but can be found in scaffold Zv4_NA1312.
c. irgf3 is represented by a partial cDNA sequence and a set of overlapping trace sequences from the zebrafish whole genome sequencing project (including zDH64-1061h13.q1k, ZDH88-124d21.p1k, zfish35935-195b06.p1c)
d. irgf4 lies within Zv4_Scaffold102.1 of the genome sequence and is not represented by any known cDNAs.
e. irgq2 lies within a fully sequenced and annotated part of the genome and is represented by a fully sequenced cDNA clone.
f. irgq3 lies within Zv4_Scaffold1709.9 of the genome sequence and is not represented by any known cDNAs.
