# Supplementary material for: The interferon-inducible p47 (IRG) GTPases in vertebrates: loss of the cell autonomous resistance mechanism in the human lineage
Source: Genome Biol. 2005 Oct 31;6(11):R92. doi: 10.1186/gb-2005-6-11-r92 (PMC1297648; doi:10.1186/gb-2005-6-11-r92)
Supplement: Additional data file 6 — Protein similarity matrix of Irgc and Irgq (contains comparison between the mouse p47 GTPase Irgc and the long coding exon of the closely linked quasi-GTPase Irgq (FKSG27) [file gb-2005-6-11-r92-S6.pdf]

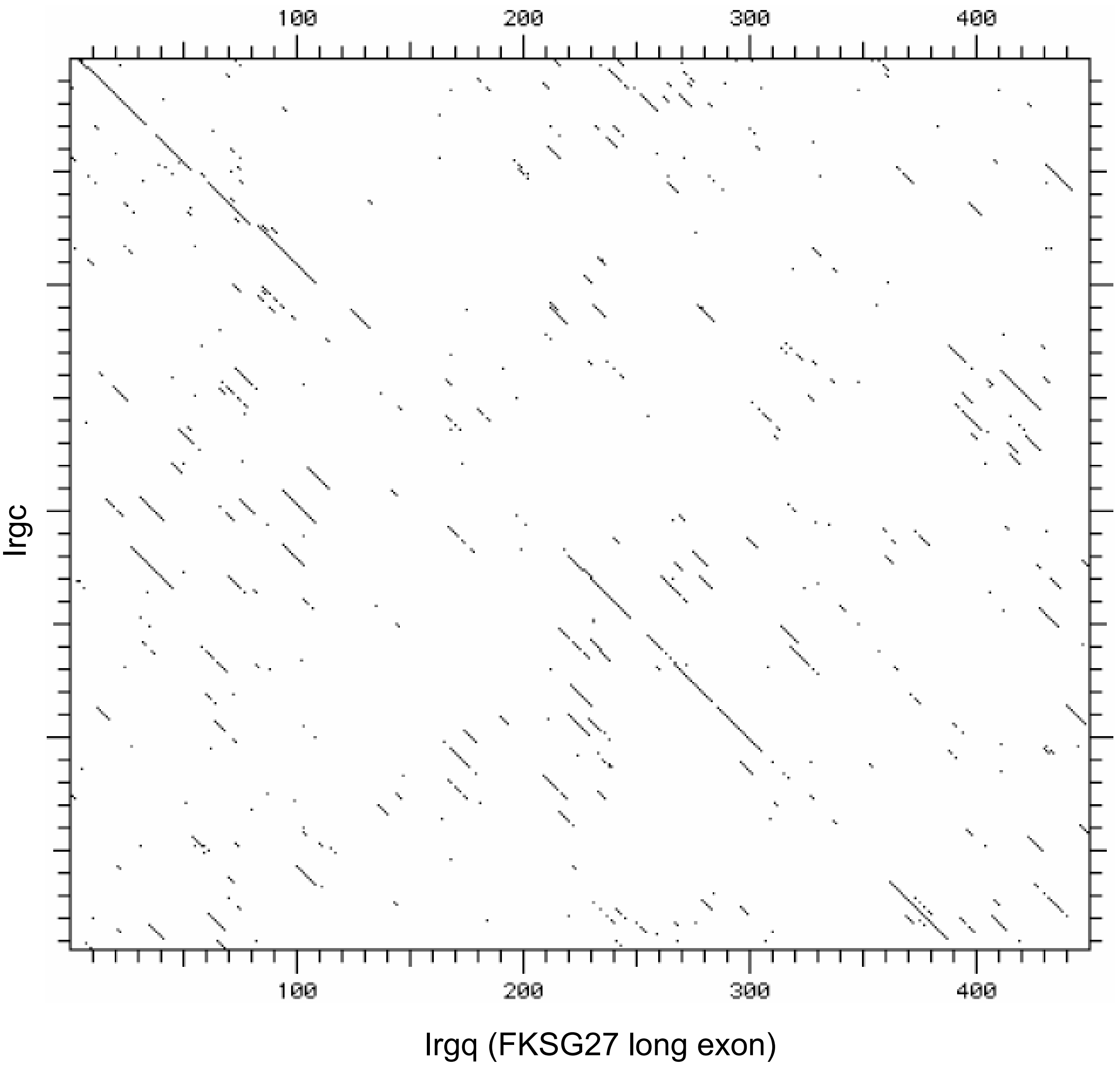

Additional Data File 6: Protein similarity matrix comparison between the mouse p47 GTPase Irgc and the long coding exon of the closely linked quasi-GTPase, Irgq (FKSG27). The two protein coding exons are co-linear and homologous both 5' and 3' of the G domain. Window Size=15, Stringency=3, Hash Value=1, Scoring Matrix=Pam250 matrix.
