# Supplementary material for: The interferon-inducible p47 (IRG) GTPases in vertebrates: loss of the cell autonomous resistance mechanism in the human lineage
Source: Genome Biol. 2005 Oct 31;6(11):R92. doi: 10.1186/gb-2005-6-11-r92 (PMC1297648; doi:10.1186/gb-2005-6-11-r92)
Supplement: Additional data file 7 — Divergent nucleotide-binding motifs in quasi-GTPases (compares the nucleotide binding motifs of quasi-GTPases to those of the classical mouse p47 GTPases) [file gb-2005-6-11-r92-S7.pdf]

	G1	G3	G4
**IRG GTPases**			
Irgd (m)	I**G**QSGT**GKS**	KVIFW**DLPG**T	FVR**TKVD**
Irga6 (m)	T**G**ETGS**GKS**	NVVFW**DLPG**I	FVR**TKVD**
Irgc (m)	T**G**ESGT**GKS**	DVTLW**DLPG**A	FVR**TKVD**
Irgm1 (m)	T**G**DSGN**GMS**	NVVLW**DLPG**L	IVW**TKLD**
irgg1 (z)	T**G**DSGA**GKS**	HIRLW**DLPG**M	FIR**TKID**
irge1 (z)	T**G**MTGA**GKS**	NVKIW**DLPG**I	FIR**TKID**
irgf1 (z)	T**G**ESGS**GKS**	NVKVW**DLPG**I	FVR**SKID**
**IRG Quasi-GTPases**			
Irgq (m)	A**G**TTNV**GLV**	NVVLW**TVPL**G	PEV**LEEK**
IRGQ (h)	A**G**KADV**GLV**	NVVLW**TVPL**G	ECL**GEGK**
irgq1 (z)	L**G**ETGS**GVS**	DVRFW**DISG**I	LVQ**TKVD**
irgq2 (z)	A**G**ERNA**EKA**	DFRLW**DLPP**I	VYF**VLLA**
irgq3 (z)	L**G**ETGC**GSS**	NIRFW**DLPG**L	ILV**SPLR**

Additional Data File 7: Divergent nucleotide-binding motifs in quasi-GTPases
Nucleotide binding motifs (highlighted in bold) of mouse (m) and zebrafish (z) and human (h) IRGQ proteins compared with classical IRG motifs. The alignments are taken from Fig. 6. Except in the case of zirgq1 the IRGQ alignments are conjectural for G4 because the homology is so low in this region
