# Supplementary material for: The interferon-inducible p47 (IRG) GTPases in vertebrates: loss of the cell autonomous resistance mechanism in the human lineage
Source: Genome Biol. 2005 Oct 31;6(11):R92. doi: 10.1186/gb-2005-6-11-r92 (PMC1297648; doi:10.1186/gb-2005-6-11-r92)
Supplement: Additional data file 8 — A list of the primers used (contains the sequences of all primers used in this study) [file gb-2005-6-11-r92-S8.pdf]

Additional Data File 8: List of Primers

Gene	Primers 5' to 3'
Irgc	GCCTCTAGCTGCTGGGACCTGTCTCAGGTCACATCTGAG
	GCGGGTGGCCGCCAGATCCTCGTCCACC
*IRGC (*human*)*	GGAGATCCTATCAGTGGGGAGAGTGTGAGGG
	CCTCTCTGAAGCCCGACGGCCG
Irga2	TAAGAAGAAGCTCAGTAGCC
	ACCGAGGGCTATTCTTCTCT
*GBP-1 (*human*)*	CTGTATCCGGAAATTCTTCCCAAAG
	CTTCAATGGCCTCTCTCTCACTGTC
*GAPDH (*human*)*	ATGACAACTTTGGTATCGTGGAAGG
	GAAATGAGCTTGACAAAGTGGTCGT
*IRGMs1-r1 (*human*)*	CAGGACACCAGTTAACATCACTATG
	GATTTTCCAGGACATTTTCTCTGAT
IRGMs1- rGMS *(*human*)*	CAGGACACCAGTTAACATCACTATG
	ATATTTCTGGGCCTTGTGGAATTCAC
Irgb1-3-8	AAAGTTCTACTTTGTCCGAACCAAGATAGATCAAGAT
	CTCTTCCTTATTAAGGAGAGACTTGGCATCACTTG
Irgb2-5-9	GGTACATACAACCACTGAGAGAACACCATACACTTACA
	ATGGTATGGTAGCCCATGCTCTTGGCA
Irgb6	TCTACTTTGTCAGAACCAAGATAGACAGCGACTTAGA
	GCCATGCGATAGTAAGTGACTGCAGCG
Irgb7	TCATTATTGTCTCTGCTGGACGCATTAAACAT
	TTAGAGACTAAGAAGACTGGAGGCTCCTGGTG
Irgb8	CGCTTATCTAGACCAAGTGGGATTTGCCA
	CAACCACTATGTTAAGGAACTGTGTGCGCC
Irgm1	CGGAATCAAGGAGACTGTGGCAACATTG
	TCCTGGGCAACTAAGAAAAGCATGCGTT
Irgm2	GATCTCGGATCCGGGTAACGCGAT
	TAACAGAACTTCCTTGGCTTTGGCAGCAG
Irgm3	CTGGAGGCAGCTGTCAGCTCCGAG
	GTCCTTTAGAGCTTTCCTCAGGGAGGTCTTG
Irgb10	TGCTGCCTTGACAGACATTGAGAAAGCC
	GCTGCGTTAGCATTCTGCAGATTCTTTACAC
Irga1	TTCCCTTGTCAATGTGGCTGTCACTGG
	AGAAAGGTCAGTGAAGGTATGATAGTAGCACACCAG
Irga2	CACAGGTGGACTCTGACTTAAGAAATGAAGAGGATT
	ACTTTCTAAGAAGAAGCTCAGTAGCCCATCTGC
Irga3	AGCTATGCTTGAAAAGGGGGGACTTTCAG
	TATGGGCAAAATGAGTTTGAGCCGCTT
Irga4	GCTGAAGTTGGAGTAATAGAGACAACTATGAAGAGAACTTCT
	TGTGTCAAGGATATGAAGTTGTAAATAATAGATTGCAGG
Irga5	CTGACACTAGGAGATGTTCAGCAAGCAAATAATG
	AAGGAAAAGAAGTGGTAAGTAAAAGGCTTTCTCCATATA
Irga6	ATCAGTGATGCATTAAAAGAAATCGATAGTAGTGTGC
	GTCAGAGAAGGGATGATATTCACTAGGTCAGCAG
Irga7	CACAATTTTATGCTTTCTCTGCCTGGCATT
	TCAGCAAATGAGGGGACTTCATTATTTCTTTTACTT
Irga8	GAGTTATGCCTGAAGAAGGGGGACATTCA
	TGAGTTTGAGCTATTTTTTTGAATATGCCTCTTTAAGG
IRGC	GCTGGCAAGTCCTCCCTCATCAAC
	GAGAGGTTGGACACGAGGAAGATGC
IRGB2	TCCTTTCTCAGGAGGCCATCACTTC
	GCCAGTTGTGCATCATTGATTGTGA
IRGM5	GAAAAGGCATTGGGAGATGGGAAGT
	AACCTTTTCCCCTGTCTTTGGATGG
IRGM6	GAGAGAGCATCCAGTGTCCCATTGA
	GATGGGTTCGAAAACCCTCTCCTTC
IRGM4	ACCCAGTCCCTTCACACTCCATCAC
	TAGCAAGTGGGAATCTGGGTGGTTC
