# Supplementary material for: The interferon-inducible p47 (IRG) GTPases in vertebrates: loss of the cell autonomous resistance mechanism in the human lineage
Source: Genome Biol. 2005 Oct 31;6(11):R92. doi: 10.1186/gb-2005-6-11-r92 (PMC1297648; doi:10.1186/gb-2005-6-11-r92)
Supplement: Additional data file 9 — Nucleotide sequences of all IRG family members [file gb-2005-6-11-r92-S9.doc]

**NUCLEOTIDE SEQUENCES FOR HUMAN IRG GENES**

>IRGC 1392 bp

atggctacttcaaagttgcccgtggtgcctggggaggaggaaaacaccatccttatggccaaggaaaggctggaggccctgcgcacagcctttgagtcgggtgacctcccccaggccgcctctcacctccaggagctgctggcctccacggaaagcatccgcctggaggtgggcgtcacgggcgagtcgggcgcgggcaagtcctccctcatcaatgccctgcgtggcctggaggccgaggaccctggcgcggctctcacgggcgtcatggagaccacgatgcaaccgtcgccctatccacacccacagttccctgacgtgaccctctgggacctgccaggagccggctctccaggctgcccggctgacaagtacctaaagcaggtagacttcagccgctatgacttcttcctgttggtctccccccgccgctgcggggccgtcgagacccgcctggccgctgagatcctgtgccagggcaagaagttctactttgtgcgcaccaaggtggacgaggacctggcggccacgcgcacccagcggccgtcgggcttcagagaggccgctgtcctgcaggagatccgagaccactgtgccgagcggctgcgggaggccggcgtggctgaccctcgcatcttcctggtgtccaacctctcgccggcccgctacgactttcccacgctggtgtccacctgggagcacgacctgccctcccaccggcgccacgctggcctgctgtcgctccccgacatctcgctggaggccttgcagaagaagaaggccatgcttcaagagcaagtcctcaagaccgccctggtgttgggcgtcatccaggccctgccggtcccagggctggcggccgcctacgatgatgcgttgctcatccactcactgcgtggctaccaccgcagctttggtctggacgacgactcgctggccaagctggccgagcaggtgggcaaacaggcaggtgacctgcgctcggtcatccgctccccactggccaacgaggtctcgcctgagactgtcctgcggctctattcccagtcgtccgacggcgccatgcgggtggcccgcgcctttgagaggggcatccctgtgtttgggacgctggtggctggcggcatcagctttggcgctgtctacaccatgctccagggctgcctcaacgagatggctgaggacgcccagcgtgtccgcatcaaggccctggaggatgacgagccgcagccggaggtcagcttggaagtggccagtgacaatggcgtggaaaaggggggctccggggagggaggtggggaggaagccccactctcaacctgcaggaagctcggcctccttcttaagtacattctggacagctggaagaaacacgactcagaagagaaataa

>IRGM (a) 537 bp

atggaagccatgaatgttgagaaagcctcagcagatgggaacttgccagaggtgatctctaacatcaaggagactctgaagatagtgtccaggacaccagttaacatcactatggcaggggactctggcaatgggatgtccaccttcatcagtgcccttcgaaacacaggacatgagggtaaggcctcacctcctactgagctggtaaaagctacccaaagatgtgcctcctatttctcttcccacttttcaaatgtggtgttgtgggacctgcctggcacagggtctgccaccacaaccctggagaactacctgatggaaatgcagttcaaccggtatgacttcatcatggttgcatctgcacaattcagcatgaatcatgtgatgcttgccaaaaccgctgaggacatgggaaagaagttctacattgtctggaccaagctagacatggacctcagcacaggtgccctcccagaagtgcagctactgcagatcagagaaaatgtcctggaaaatctccagaaggagcgggtatgtgaatactaa

>IRGM (b) 744 bp

atggaagccatgaatgttgagaaagcctcagcagatgggaacttgccagaggtgatctctaacatcaaggagactctgaagatagtgtccaggacaccagttaacatcactatggcaggggactctggcaatgggatgtccaccttcatcagtgcccttcgaaacacaggacatgagggtaaggcctcacctcctactgagctggtaaaagctacccaaagatgtgcctcctatttctcttcccacttttcaaatgtggtgttgtgggacctgcctggcacagggtctgccaccacaaccctggagaactacctgatggaaatgcagttcaaccggtatgacttcatcatggttgcatctgcacaattcagcatgaatcatgtgatgcttgccaaaaccgctgaggacatgggaaagaagttctacattgtctggaccaagctagacatggacctcagcacaggtgccctcccagaagtgcagctactgcagatcagagaaaatgtcctggaaaatctccagaaggagcggctggcctgccatgagaaatacctcaagagtactccagagaattccacaaggcccagaaatattccttccagaagaaaactatacgtaaatctgctcaggatattcaacagctagccagtgcatatgacatatactgggtacttaagaaacgtttgttaaatgaatcatgattataaagaaaaaataaattaaaacttatgtttagttaaaaaaaaaaaaaaa

>IRGM (c) 882 bp

atggaagccatgaatgttgagaaagcctcagcagatgggaacttgccagaggtgatctctaacatcaaggagactctgaagatagtgtccaggacaccagttaacatcactatggcaggggactctggcaatgggatgtccaccttcatcagtgcccttcgaaacacaggacatgagggtaaggcctcacctcctactgagctggtaaaagctacccaaagatgtgcctcctatttctcttcccacttttcaaatgtggtgttgtgggacctgcctggcacagggtctgccaccacaaccctggagaactacctgatggaaatgcagttcaaccggtatgacttcatcatggttgcatctgcacaattcagcatgaatcatgtgatgcttgccaaaaccgctgaggacatgggaaagaagttctacattgtctggaccaagctagacatggacctcagcacaggtgccctcccagaagtgcagctactgcagatcagagaaaatgtcctggaaaatctccagaaggagcggagctctagaaaccaacaagtgtacccaaaccacagccacgtacctcttgcttgctgaaaggatcctgagagaaaggcaagacaaaaaaatacagatcagctccgcaagccctagaaatagcaaggcccagctcagctggcctgccatgagaaatacctcaagagtactccagagaattccacaaggcccagaaatattccttccagaagaaaactatacgtaaatctgctcaggatattcaacagctagccagtgcatatgacatatactgggtacttaagaaacgtttgttaaatgaatcatgattataaagaaaaaataaattaaaacttatgtttagttaaaaaaaaaaaaaaaaaa

>IRGM (d) 1050 bp

atggaagccatgaatgttgagaaagcctcagcagatgggaacttgccagaggtgatctctaacatcaaggagactctgaagatagtgtccaggacaccagttaacatcactatggcaggggactctggcaatgggatgtccaccttcatcagtgcccttcgaaacacaggacatgagggtaaggcctcacctcctactgagctggtaaaagctacccaaagatgtgcctcctatttctcttcccacttttcaaatgtggtgttgtgggacctgcctggcacagggtctgccaccacaaccctggagaactacctgatggaaatgcagttcaaccggtatgacttcatcatggttgcatctgcacaattcagcatgaatcatgtgatgcttgccaaaaccgctgaggacatgggaaagaagttctacattgtctggaccaagctagacatggacctcagcacaggtgccctcccagaagtgcagctactgcagatcagagaaaatgtcctggaaaatctccagaaggagcggctggcctgccatgagaaatacctcaagagtactccagagaattccacaaggcccagaaatataaatctctgcagctgaccatcaccttgacagacttttaaaatggagcacaatgaaccatcccttgtgactcctttcagtatcttatgatggaaggaaactgtccccaaaatacatgactgggagttgtgaaggttactcttcctgtctaaaagaagaaaagatacaatttaagagtcatcacaagagtcaattaagagggtaaagaagtagaacaaatggatgatccaaccatagtagatgaactcacaaaaaaaggatggaaacaggtcatctccagaagaccagtaccagccatatagtgttatccgtccttccagaagaaaactatacgtaaatctgctcaggatattcaacagctagccagtgcatatgacatatactgggtacttaagaaacgtttgttaaatgaatcatgattataaagaaaaaataaattaaaacttatgtttagttaaaaaaaaaaaa

>IRGM (e) 1181 bp

atggaagccatgaatgttgagaaagcctcagcagatgggaacttgccagaggtgatctctaacatcaaggagactctgaagatagtgtccaggacaccagttaacatcactatggcaggggactctggcaatgggatgtccaccttcatcagtgcccttcgaaacacaggacatgagggtaaggcctcacctcctactgagctggtaaaagctacccaaagatgtgcctcctatttctcttcccacttttcaaatgtggtgttgtgggacctgcctggcacagggtctgccaccacaaccctggagaactacctgatggaaatgcagttcaaccggtatgacttcatcatggttgcatctgcacaattcagcatgaatcatgtgatgcttgccaaaaccgctgaggacatgggaaagaagttctacattgtctggaccaagctagacatggacctcagcacaggtgccctcccagaagtgcagctactgcagatcagagaaaatgtcctggaaaatctccagaaggagcggagctctagaaaccaacaagtgtacccaaaccacagccacgtacctcttgcttgctgaaaggatcctgagagaaaagcaagacaaagaaatacagatcagctccgcaagccctagaaatagcaaggcccagctcagctggcctgccatgagaaatacctcaagagtactccagagaattccacaaggcccagaaatataaatctctgcagctgaccatcaccttgacagacttttaaaatggagcacaatgaaccatcccttgtgactcctttcagtatcttatgatggaaggaaactgtccccaaaatacatgactgggagttgtgagggttactcttcctgtctaaaagaagaaaagatacaatttaagagtcatcacaagagtcaattaagagggtaaagaagtagaacaaatggatgatccaacatagtagatgaactcacaaaaaaaggatggaaacaggtcatctcagaagaccagtacagcatatagtgttatccgtccttccagaagaaaactatacgtaaatctgctcaggatattcaacagctagccagtgcatatgacatatactgggtacttaagaaacgtttgttaaatgaatcatgattataaagaaaaaataaattaaaacttatgtttagttaaaaaaaaaaaa

>IRGQ 1344 bp

aggctcctgccaccggcgcaggatggcttcgaggtgttgggtgcagcagagctagaggctgtgcgtgaggcctttgagaccggcggccttgaggctgcgctgtcgtgggtgcgctcaggcctggagcgcctgggcagcgcacggctagacctggccgtggctggcaaggctgacgtgggccttgtggtggacatgctgcttggattggatcctggcgacccaggcgctgcgcctgcttcggtccccacagcacccactcccttcccagccccagagcgcccgaatgtggtgctctggaccgtgcctctgggccacacgggcactgccaccaccgcggccgccgcctctcacccaacgcactacgacgccctcatcctcgtcacccctggggcccccactgagaaggactgggcccaggtccaggccttgctgctaccagatgcgcctcttgtctgcgtgcgcacagacggcgagggcgaggatccggagtgtctgggagaaggcaagatggagaatcccaagggcgagagcttaaagaacgcaggtggagggggattggagaatgcactcagtaagggaagggagaaatgtagcgctggatcgcagaaagcaggcagcggggaaggtcctgggaaagctggcagcgagggtttgcagcaggttgtcggcatgaagaaatcaggtggtggcgactcagagcgggccgctgcgttaagcccggaggacgagacgtgggaggtgctggaggaggcgccgccgccagtgttccccctacggcctggcggactcccagggctatgcgaatggctgcggcgagcgctccccccagcccaggcaggggcactgctgctggcgttgccaccagcatctcccagcgctgcccgaaccaaggctgcggcgttgcgagccggggcgtggaggccagctctgctggctagtctggcggcggcggcggcaccactcccagggctgggctgggcatgcgacgtggcacttctgcggggtcagctggcggagtggcgacggggcctggggctggaacccacggcactggctcgacgtgagcgtgccctgggcctggcttctggagagctggcagcgcgcgctcatttcccaggcccggtgacgcgcgccgaggtggaagcaagactgggcgcctgggcgggcgagggcactgctgggggcgcagcactgggggctctctccttcctgtggcctgcgggtggtgcagcggcgacaggtggcctgggctaccgagcggctcacggcgtcctgctgcaggctctcgatgagatgcgggctgatgctgaggctgtgctggcaccccctgagcctgcccagtga

**NUCLEOTIDE SEQUENCES FOR DOG IRG GENES**

>IRGC 1395 bp

atggctacttcgaagttgcgagcagtgcccggggaggaggagaccaccatcctcatggccaaggaagagctggaggccctgcgcagcgcctttgagtcgggcgacatcccccaggcggcctcccgcctccgggagctgctggcctcctcccagagcatccgcctggaggtgggcgtcacaggcgagtcgggcgctggcaagtcctccctcatcaacgccctccggggtgtgggggccgaggaccccggcgcggccctcaccggcgtcgtggagaccacaatgcagccctcgccctacccgcacccacagtttccagacgtgaccctgtgggacctgccgggggctggctctccgggctgcccagctgacaagtacctgaagcaggtggacttcggccgctatgacttcttcctgctggtctctccccgccgctgcggggctgtggagacccgcctggcctcggagatcctgcgccagggcaagaagttctactttgtgcgcaccaaggtggacgaggacctggcggccacccgtacccagcggccctcgggcttcagtgaggcggccgtcctgcaggagatccgtgaccactgcgccgagcggctgcgggtggccggcatgaccgacccgcgcatcttcctcgtgtccaacctctcgccggcccgctacgactttccgctgctcatgtccacctgggagcacgacctgcctgcccaccggcgccacgctggcctgctttcgctgcccgatatctcgctggaggccctgcagaagaagaaggacatgctccaggagcaagtgctcaagacagccctggtgtcaggcgtcatccaggccctgcccgtgccagggctggcggccgcctacgacgacgctctgctcatccgctcgctgcgtggctaccaccgcagcttcggcctggatgacgactcgctggccaagctggctgagcaggtgggcaagcaggcaggtgacctgcgctccgtcatccgatccccgctggccaacgaggtctcgccggaaaccgtcctgcggctctactcacagtcatctgacggtgccatgcgggtggcccgtgcctttgagaaggggatccctgtgtttggcacgctggtggccgggggcatcagctttggcaccgtctataccatgctccagggctgcctcaatgaaatggccgaagacgcccagcgggtccgcatcaaggccctggaggaggacgagccccagtctgaggtcagcctggaggcagccggtgacaatggtgtggaaaagcggggatctggggagggaggctgtgaggaagcaccgctctcggctcgccggaagcttggcctcctcctcaagtatattctcgacagctggaagaagcgtgacttgtcagaagagaagtga

>IRGB12 1323 bp

atgggtcagtcctcttccacaccctctcataaaacaggtggtgatttggcctccagttttggcaagttttttaaggactttaaactggaaagcaaaatcctttctcaggaggccatcacttcgattgaaaaatccttgaaggaggggaaccttcagaaagcagtgtctgatattaataaggcactgaaagacattgacaatgcccctctgagcattgctgtgactggggagtctgggacagggaagtccagtttcatcaatgccctgcggggagtagggcatgatgaagaaggggctgcccccattggggcagtggagacaacttttgacagaaccgaatacaaacacagaaagtttcccaatgtgacattatgggacctgccaggtgtagggaccactacatttcacccacaagagtatttggagaaaatgaaatttcgtgagtatgatttctttattatcatctcttccacacgcttcacaatcaatgatgcacaactggctacagcaattagaaaaatgaagaagaatttctactttgtccgatctaaagtggacagtgatttatataatctaaaaagaactaaacccagtgatttcaataaggatgaaatcctgctaaagatccgtaatgactgtataactcagttgcagaatgtcaaagtgtgtgaccctcaggtcttcttagtctccaaccttgatttgtctagctatgatttccaaagcctagagaccacccttctgaaggagctcccagcccacaaacgccacatcttcatgcaatatctaccgaatattactgagtctgccattgaccggaagagggattccctgagacaaaaggtctggctagaggctgtaaaggctggagcatcggccactatccctttcatgggcttgatcaatgataacgaggtggagaagctggaggagactttacacctctacaggtcttactttgggttggatgatgcatccctggaaaccatagccaaggacttgaatgtgtcagtggagaaactcaaggcaaaccttacgtctccccatttgctatcagttgagaaggaggatgagtcgttaggggaaaaactactgagatatgtggaaaaattctgttctgttagtggaggactaattgccactggtgtttactttaggaagattttctacttgcaaaattatttccttgaggctgtggtgagtgatgcaaaagttctccttaacaaagaagagatttttaaggaaactgtagggtctgggcaagcttatctgcttcaggatgttgggattgaaaataggaaaagtgatgcaaccagttcctga

>IRGB11 1326 bp

atgggtcagtcccctccttccacaccctcgaatagaaatggtggtgatttggcctccagctttgacaagttttttaaggaattcaagctggatagcaaaattatctctcaggagaccatcagcacaattcaatcacatctggagaaaggggatcttcagagtgcattttctgcaatcaatgatgcattgcgagatattgacaatgccccactgaacattgctgtgaccggggaatctgggacaggaaagtccagcttcatcaatgccctgcggggcatggggcatgatgaagaaggggctgcccccactgggccagtggagacaacttttctcagaaaggcctataaacacccaaagtttcccaatgtgacattttgggatctgcctggcatagggaccactagctttcagcctcaggactatctggagaaaatggtatttagggagtatgacttctttattatcatctgtgctacacgcttcaaaatcaatgatgtgcaactggctactgcaattaaaaaaatgaagaagaatttctactttgtccgatctaaagtggatagtgatttatataatctaaaaagaattaaacccagagaattcaataaggatgaaatcctgcaaaagatccgcaatgactgtgtgaaacatctgatggaggccaatatgagtgatgctcaggtcttcttagtctccagctttgagttatctgactatgatttccaaagcctggagaccacccttctgagggagctcccttcccacaagcgccacatcttcatgcaatacctgccgattgttactgaggccaccattgaccggaagagggattgcctgagacaaaaggtctggctggaggccattaaagctggagcatcagcctccatccctttggtgggctacatcagtgataacgatgtggaaactttaaaggatactttaactctctacaggtcttactttggattggatgatatatccctgaaaaccatagccaaggacttgaatgtgtccgtggagaaactcaaggcaaaccttatgtttccccatttgctatcagttgaaaagtacgatgaaccattaggggaaaaactgttgaaatatgtggaaaaattctgctctgttagtggaggacccattgctgctggtatttactttaggaagatttactatttgaaaaattatttccttgacaccgtggtgagtgatgcaaaagttctccttaaaaaggaagagatttttaaggaccctgtggactctgagcaaacctatctacatacgaatgttggaaatgaaaatgggaaaagtgacacatccagctcctga

>IRGM6 1239 bp

ctccactgctttttcccacttctgcaggtcactccactgctcagcgatgtgacacagcccacccactctcttcacacaccattacttacatcctccaactatgatatgccatacaatatgggctggagtagcttatctaaggagactgccataaacattgaaaaggccttgggaggaaggaagttgctggaggtggtccctatggtcagggagaccctggagagagcatccagtgtcccattgagaattgctgtgactggggactctggcaatggcatgtcttctttcatcaatgcactgcggggaattgggcatgatgaggaggattcagctcccacaggggtggtaaaaaccacccagattcccacttgctactcttacccccactttcccaatgtggaactgtgggacctacctggaacaggggcaggcacccaaagtctggagaactacctggaggagatgaaatttagctggtatgacctcttcatcattattgcatctgaacagttcagcatgaatcttgtgaagcttgccaaggccatccaggtcctgggaaagagattctacattgtctggaccaagctggacagggacctcagcacaagtgctctcttgaaagaacgtctcctgcagaatattcaggagaatattcaggaaaatctccagaaggagagggttttcgaacccatcatattcctggtctccagctttgagcccttattgcatgacttcccagagcttaggaacaccttgaacagggacatttctgatatcaggtactgtggtcccctaaagaatctgtcccacacttatgagaaggtcattagtgacaaagtgaccatgttcagggggaaaatagcctcaaagtcttttgacacccttggcatttggaatgcagatgatcttggggagtgtctgatagcctaccacttgttctttggtgtggatgatgagtctctccaacagatagctcagagtatggggaaacccatggaggagtacagggccattatgaagtctcgggatctgcacactatcattagaggggactgggcagtatcttgcatgaattgtaatacatcctcttgcttatatacaattctgaggtacatcccactgttaggcgactttattatcaactttctgagaaagtggaaacacagacgcctcctggaaatagtggctgaggacaccagaaccatcttgaagaaaatcctgaaagactccatcatctga

>IRGM5 1194 bp

atgacacagcccaaccattcccttcacatcccgttatctacatccttcacatctattgttccatacaatatgggatggacagtcttacctaaggcaactgccacaaacattgaaaaggcattgggagatgggaagttgctagaggtggtctctatgattagggagaccctagagacagtatccagtgccccagtaagcattgcagtgactggggattctggcaatggcatgtcctccttcatcaatgcactgcgggaaattgggcatgatgagaaggactcagctcccactggggtggtaaggaccacccaggttcccacttgctactcttcctcccattttccctacatggaactatgggatctacctggaacagggacaggcacccaaagcttggagaactatctggagaagatacattttagccagtatgaccttttcatcatcattgcatctgaacagttcagcatgaatcttgtgaagcttgtcaaagccatccaaagacaggggaaaaggttctacattgtctggaccaagctggacagggacctcagcacacgtgtccttccagaggaacaagtcctgcagaatatatgggagaatatccaggaaactctccagaaagtaggagtgtgtgaacccatcatattcctggtctccagctttgagcccttattgcatgacttcccagagcttagggacgccttgaacagggacatttctgatatcagatactgtggtcccctagagaacctgtctgacacctgtgaaaagatcattaatgacaaagtgacttcttttcaggagcaaataggctcaaagactttccaggacatcctgggcatccaggatgaagatgatctggggcagtgtctgatagcctaccacttgttctttggtgtggatgataagtctctccagcagatggcccagagtatggggaaacccatggaggagtacagggccattatgaagtctcaggatgtgcacactgtcctcaccggggactgggcattatcttgcatgaattgtaaaacagcatcttacttatattcgattctgagctatatcccgttcttaggtgatactgttatcaactacctgagagtgtggaaacacagacacttccttgaaatagttgccaaggacaccagaagcattgtgaagaaaatcctgacagactccatcatctga

>IRGM4 1194 bp

atggcacagcccacccagtcccttcacactccatcacctacatccttcacttctactgtgccataccataagggtgggagcatcttatctgaatcgggtgccatgaatattgaaaaagccttgggagaagggaagttgctggacatggtctctgtggtcagggagaccctggagacagcatccagtgtcccagtgagcattgcagtgactggggactctggcaatggcatgtccacattcatcaatgcattgcggaaaattgggcacaatgaagaggactcagctcccacaggggtggtgagaaccacccagattcccacttgctactctttctctgacatccccaatgtggagctgtgggacctgcctggcacaggcgctgccacccaaaacctagagacatatctggaggaaatgcagtttagcaagtatgacctcttcatcatcattgcatctgaacagttcagcatgaatctcgtgaagcttgtcaaaagcatccagggacaaggaaagaggttttacatcgtctggaccaagttggacagagacctcagtacatgtgtcctttcagaagaacaactcctgcggaatatccgggagaatatcagggaaactctccacaaggagggagtgtgtgaacccatcatattcctggtctccagcttcaaccccttcttgcatgacttcccagagctcaggaaaagcttgcacagagacatatctaacattgggtaccgtggtcacctagagaacctaactcacacctgtgaaaaggtcattaatggcaaagtgaccactttgcaggggcaaataggctccaagtctttccaggacatccttggcatccagaatgcaaatgatcttggggagtttttgaatgcctaccacaggctttttggtgtggatgatgactctctccaggaggtggcccaaagtatgggaaaacccaaggaggagtacaaggccatcatgaagtctcaggatctgcacactgccctagcctgggactgggcattatcatggatgaattgtaatgcagcctcttacttatattcagttctgagctacatcccaatcttaggtaccactggtatccactaccttaaatggtggagtcagggacacctccttgaaatagttgctgaggacaccaagaccatcctgaagaaaattctggaagatgccatcatctaa

>IRGD 1281 bp

atggataaattcatgtgtgatttcctggtaggaaagaattttcagcaactggccatcaactttatccctcactataccacattagtcaataaggcagggggtatcatcgcttcagaaaaccttgatagaattcaagcagcccttaaggaggccaagctaaaagatgtggctgacataattgaggaatcacttgtggcagcagagaatgctcctctggatgtggctgtgattggggaatctggcactgggaagtccagtttcatcaatgccctgcgaggacttagttatgaagaggagggttctgcaagtgttggagtcgtggagactaccatgaagaaaacgccctatcaacatccaaaatatcccaaagtgaccttctgggacctgcctggaactggaacccccaatttccatccacatgaatatctagaaatggtggaatttgctacatatgacttcttcatcattatttcttcctcccggtttagtctcaatgatgctttgctggcccaaaatatcaaggagataggcaagaaattctactttgttagaaccaaggtggacaatgatttatataatgaagagaaaagcaaacccatgtctttcaaaagggagagagtgcttcagcagatacgagacaactgcctggctaatcttagcaacattggagtgcctgagccatgcatcttcttggtctccaactttgacctggacgactttgatttcccaagactggaggaaaccctgctgaaggagctccctgttcataagcgccacatctttgcactcctgttgcccaatttatcttacacttccattgaaatgaagagagctttcttcaaggaaaagatctggctggatgccctgaaatcatcagctttgagcttcatccccttcatggcctgctttaatggctttgattttccccagcaggaaaagtgcttgaacctttaccagagccattttggtttggatgagaagtcggtcaaagggattgcagagaagctggacatgtctgtggaggagatcaagagtttcaccaagtccttggatttctggttacttgtgaaggatgacagcatagcagaaaaagccatgaagtgtgttgaatgctattgctcagtaaatggaggcctgccatccactatcttccagttctttaaaatctactttctacatttgaaattcatcaatacagtggcagatgatgctaaaattcttttgcataagactttagagatcttaagtcacagaagatga

**NUCLEOTIDE SEQUENCES FOR MOUSE IRG GENES**

>Irga1 888 bp

atgggtcagctgttctctttacttaagaataagtgccaatttttggtctccagtgttgctgaatattttaagaaatttaagaaaatagtaataatcattcttcaggaagtcaccacttcgattgaattagacatgaaaaaagagaactttcaggaggcaaactctgcaatctgtgatgcattaaaagaaatcgatagttcccttgtcaatgtggctgtcactggggagactggatcagggaagtccagttttatcaacaccctgagaggcattgggcatgaagaggaaggtgcagctaaaactggggtggtggaggcaaccatggagagacatccatacaaacaccccaatatgcccaatgtggttttttgggacctgcctgggattggaagcacaaagttcccaccaaaaacttacctggagaaaatgaagttctatgagtacgatttcttcattattatttcggccacatgcttcaagaaaaatgatatagacctcgccaaagcaatcagcatgatgaagaaggaattctactttgtgagaacaaaggtagacactgacttaagaaatgaagaggatttcaaacctcaaacctttgacaaagaaaaggtcctgcaggacattcgtcttaactgtgtgaacacctttaaggagaatggcattgccgagccacctatcttcctgatctctaacgaaaatgtatgtcactatgacttccctgtcctgatggacaagctgataagtgacctccctgactacaagagacacaattttatgctctccttacccaatatcacagattcagtcattgaaacgaagcggcaatctctgaagcagaggcactggctgcaaggctttgctggtgtgctactatcataccttcactga

>Irga2 1221 bp

atgggtcaactgttctcttcacgtaggagtgaggaccaagatttgtcctccagctttattgaatacttaaaggaatgtgagaaaggaatcaatatcattcctcatgagatcatcacttcgattgaaataaacatgaaaaaagggaacattcaggaggtaaactctacagtcagagacatgttaagagaaattgataataccccgctcaatgttgctctcaccggggagactggatcagggaagtccagcttcatcaacaccctgagaggcattgggcatgaagagggaggtgcagctcacactggggtgacggacaaaaccaaggagagacatccatacgaacaccccaaaatgcccaatgtggttttttgggacctgcctgggactggaagtgaagatttccaaccaaaaacttatctcgagaaaatgaagttctatgagtatgacttcttcattattatttcggccacacgcttcaagaaaaatgatatagacctcgcaaaagcaatcggaataatgaaaaaggaattctacttcgtgagaacacaggtggactctgacttaagaaatgaagaggatttcaaacctcaaacctttgacagagaaaaagtcctgcaggacattcgtcttaactgtgtgaacacctttagggagaatggcattgctgagccacctatctttctgatctctaacaaaaatgtttgtcactatgacttccccgtcctgatggacaagctgataagtgacctccctgtcttcaagagacaaaattttatgttctccttacccaatattacagattcggtcattgaaaagaagcgaaattttctgaggtggaagacttggctggaaggctttgcagatgggctactgagcttcttcttagaaagtgatttggagactctggagaaaagcatgaagttctaccgcactgtgtttggagtggatgatgcatctttgcagagattggctagggcctgggaaatagatcaggtagatcaggtgagggccatgataaaatctcctgccgtgttcacacctacagatgaagaaacaatacaagaaaggctttcaagatataatcaggagttctgtctggctaatgggtacttacttcctaaaaatcattgtagagaaatactttacctgaagttgtatttccttgacatggtgactgaggatgctaaaactcttcttaaagagatatgtttaagaaactag

>Irga3 1254 bp

atgggtcagctcttctctcatatacctaaggatgaagacaaaggaaatttggagtccagcttcactgaatattttaggaattataagcaagaaaccaaaatcatttctgaggaaaccacccgttcaatagagctatgcttgaaaaggggggactttcagagggcaaactctgtaatcagtgatgcattaaaaaatattgataataccccaataaatattgctgtgacaggagagtctggagcagggaagtccagcctcatcaatgccttgagagaggttaaagctgaagaggaaagtgcagctgaagtgggggtgacagagacaaccatgaaggtgtcttcatacaaacaccccaaagttaaaaatttgacattatgggacctgcctggtattggaactatgaaatttcaaccaaaagattatctagaaaaagtagagttcaaaaagtatgacttcttcattattgtttcttcctcacgatttacaaagttagaactagaccttgccaaagcaaccagaatcatgaaaaagaattactacttcgtgagaagcaaggtggactgtgatctagacaatgaaaagaaatccaaaccacgtaactttaacagggaaaacaccctgaatcaggtccgaaattcctatctggatacatttagggagagtaaaattgatgaacctcaggttttcttgatatctaaccacgatttatctgactatgattttccagtcctgatggacaccctgcttaaggaccttcctgctgaaaagcgccaaaactttctgctttccttgcctaatataactgaggcagccattcaaaaaaagtacaactctactaagcagattatctggttgcaggccacgaaggatggacttttggcgactgttcctgtagtgggcattttaaaggacttggataaggagaggctgaagaagaggttagactactatcgtgacctctttggagtcgatgatgaatccttaatgttcatggctaaggatgcccaagtgcctgttgaactgttgataaaaaaccttaaatctcctaatttgttgaaatgtaaggaggaaacattagaagaactgcttttgaactgtgtggagaaatttgcctcagctaatggtgggctcctggcagcaggtctttacttcaggaaaacctattacctacaatttcatttcctggatacagtggctgaagatgccaaagttctccttaaagcggctcaaactcattttgcccatagcttctga

>Irga4 1253 bp

atggtcagctcctctctgacacatctaagactgaagacaatgaagatttggtatccagcttcaatgaatattttaaaaacattaagacagaaaacaaaatcatttctcaggaaaccatcgatttaataaaattatacctgaacaaaggaaacattcatggggcaaactctttaatcagtgatgcattaagaaatattgataatgctccaataaatattgctgtgacaggagagtctggagctggaaaatccagcctcatcaatgccctgatagggattggacctgaagaggaaggggcagctgaagttggagtaatagagacaactatgaagagaacttcttacaaacatcccaaaattgaaacattgaccttatgggacttgcctggtattggaactcagaaattcccaccaaaaacttatctggaggaagtaaaattcaaagaatatgatttcttcattattgtttctgccacacgttttacaaaacttgaactagacctcgccaaagcaatcacaaatatgaaaaagaattactactttgtgagaaccaaggtggacattgatgtagaaaatgaaaggaaatccaaaccacgtacttttgaaagagaaaaagccctgaagcagatccaaagctattctgtgaaaatctttaatgataataacatggccgtaccaccaattttcttgatctctaactatgatttatctgactatgattttccattcctggtggataccctgattaaggaactccatgttcaaaagcgccacaattttatgctttccctgcctaactttacagaccaagccattgacagaaagtacaaggctacacagcagtttatctggctagaagccttcaagattggagtagtggcaatttttcctgtactgggcaacctcaggaataaagatatgaagaagataaagaacactttaaactactatcaaaaaatctttggagtggatgatgaatccctggagttggtggctaaggatttccaagtgcctgttgaacaggtaaagaaaacaatgaagactcctcatttgttgaagaagtatagagaggaaacatttaggaatgatttcaagaaattggtatcaacttttggtaggctccttgctgtaggtctttacttccctgcaatctattatttacaacttcatatccttgacacagtgactgaagatgccaaagttctccttcgatggaaatattcaaaacctagatcaaactcaacttatccatag

>Irga5 582 bp

atgggtcagctgttctctggcacatctaagagtgaagccctgtgctccagctttactgaatattttcagaagtttaaggtagaaaacaaaatcatttctcaggaaatcagcacattaattgagttatacctgacactaggagatgttcagcaagcaaataatgcaattacatatgcattgaggtaacttgccaggaccccacaaaatgtcgccttgataggagagtctggaagggggaagtacagtttcatcaatgtcttcagggggttggacatgaagaggaaaatggctacagttggggtggtggagacgaccatgaacagaactccatacagaaaccccaacattccaaatgtgattatttgggacctacctggaattggaaccacaaatttcccaccaaaacattatttaaagaaaatgcaattctatgttatgtacgatttcttcattattgtttcagccacatgctttaggaaaaatgatatagacctctccaaagcagtcgtcatgataaagaaggattttctgcttcgaacgagaaccaaagaggacatagatatagaaaatgaaaatgaatga

>Irga6 1242 bp

atgggtcagctgttctcttcacctaagagtgatgagaataatgatttgccctccagctttactggttattttaagaaatttaatacgggaagaaaaatcatttctcaagagatcctcaatttgattgaattaaggatgagaaaagggaatattcagttgacaaactctgcaatcagtgatgcattaaaagaaatcgatagtagtgtgctcaatgttgctgtcaccggggagacgggatcagggaagtccagcttcatcaataccctgagaggcattgggaatgaagaagaaggtgcagctaaaactggggtggtggaggtaaccatggaaagacatccatacaaacaccccaatatacccaatgtggttttttgggacctgcctgggattggaagcacaaatttcccaccaaacacttacctggagaaaatgaagttctatgagtacgatttcttcattattatttcggccacacgcttcaagaaaaatgatatagacattgccaaagcaatcagcatgatgaagaaggaattctacttcgtgagaaccaaggtggactctgacataacaaatgaagcagatggcaaacctcaaacctttgacaaagaaaaggtcctgcaggacatccgccttaactgtgtgaacacctttagggagaatggcattgctgagccaccaatcttcctgctctctaacaaaaatgtttgtcactatgacttccccgtcctgatggacaagctgataagtgacctccctatctacaagagacacaattttatggtctccttacccaatatcacagattcagtcattgaaaagaagcggcaatttctgaagcagaggatttggctggaaggatttgctgctgacctagtgaatatcatcccttctctgacctttctcttggacagtgatttggagactctgaagaaaagcatgaaattctaccgcactgtgtttggagtggatgaaacatctttgcagagattagctagggactgggaaatagaggtggatcaggtggaggccatgataaaatctcctgctgtgttcaaacctacagatgaagaaacaatacaagaaaggctttcaagatatattcaggagttctgtttggctaatgggtacttacttcctaaaaatagttttcttaaagaaatattttacctgaaatattatttccttgacatggtgactgaggatgctaaaactcttcttaaagagatatgtttaagaaactag

>Irga7 1266 bp

atggatcagctcctctctgacacatctaagaatgaagacaatgatgatttggtgtccagcttcaatgcatattttaaaaacattaagacagaaaacaaaatcatttctcaggaaactatcgatttaatagagttacacctgaacaaaggaaacattcatggggcaaactctttaatcagagaagcattaaaaaatattgataatgccccaataaatattgctgtgacaggagagtctggagtagggaaatccagcttcatcaatgccctgatagggactggacctgaagaggaaggggcagctgaagtgggggtaatagagacaactatgaagagaaatttttacaaacatcccaaaattgaaacgctgaccttatgggatttgcctggtattggaactcagaaattcccaccaaaaacttatctggaagaagtgaaattcaaagaatatgatttcttcattattgtttcttccacacgttttacaaaacatgaactagaccttgccaaagcaattggaatcatgaaaaagaattactactttgtgagaaccaaggtggacattgatttagaaaatgaaaggaaatccaaaccacgtacttttgacagagaaaagaccctgaagcagatccaaagctatgctatgaatacctttagcgacaataacatggctataccaccgattttcatggtctctaattatgatttatctaagtatgattttccagtcatgatggacaccctgattaaggatctccatgcagaaaagcgccacaattttatgctttctctgcctggcattacagaggcagccattgacagaaaacacaaggcaacacagcagattgtctggctagaagccttcaatgttggacttttggcaaattttcctgtaacaggcatcctcggtgataatgatgtgaagaagttggagaagagtttaaactactatcgaaaaatctttggagtggatgatgaatccctggagttagtggctaaggatttccaagtgcctgttgaacaagtaaaagaaataatgaagtcccctcatttgctgaagactaatggaaaggaaacgttaggagaaaagcttttgaaatatttagagaaatttgaaacagctactggtgggctccttgctgtaggtctttatttcaggaaaacctattatttacaacttcatttccttgacacagtgactgaagatgccaaagttctccttcgatggaaatattcaaaacctagatcaaactcaacttatccatag

>Irga8 1233 bp

atgggtcagctcttctctaatatgcctaaggatgaagacaaaggaaatttggagtccagcttcactgaatattttaggaattataagcaagaaaccaaaatcatttctgaggaaaccacccgttcaatagagttatgcctgaagaagggggacattcaaagggcaaactctataatcagtgatgcattaaaaaatattgataatgccccaataaatattgcagtgacaggagagtctggagcagggaagtccagcctcatcaatgccttgagagagattaaagccgaagaggaaagtgcagctgaagtcggggtgacagagacaaccatgaaggtgtattcatacaaacaccccaaagttaaaaatttgacattatgggacctgcctggtattggaactaagaaatttccaccgaaaacttatctagaaacagtagaattcaaaaagtatgacttcttcattattgtttctgccatacgatttacaaaccatgaaatagaacttgccaaagcaatcagaattatgaaaaagaattactacttcgtgagaagcaaggtggactttgatctatacaatgaagagaaatccaaaccacgtaactttaacagagaaaacactctgaatcaggtccgaaattactatctggatacatttagggagagtaaaattgatgaacctcaggttttcttgatctctaaccacgatttatctgactatgattttccagtcctgatggacaccctgcttaaggaccttcctgctgaaaagcgccacaactttctgctttccttgcctaatataactgaggcagccattcaaaaaaagtacaactctcctaagcagtatatctggttgcaggccatggaggatggacttttggcgactgttcctgcagtgggcattttaaaggacttggataaggagaggctgaagaggagtttagactactatcgtgacctctttggagtcgatgatgaatccctaatgttcatggctaaggatgcccaagtgccttttgaactgctgaaaataaaacttaaatctccttatttgttggaacttgaggaggaaacattaggaggattgattttgaactgtgtggagaaatttgcctcagctaatggtgggctcctggcaacagggctttacttcaggaaaacctattacctacaatttcatttccttgatacagtggctgaagatgccaaagttctccttaaagaggcatattaa

>Irgb1 1326 bp

cagcatcctccactgaacacagctacatgccagacttccactggcaggacttctcagataacggctcaacttttggaattcaactttaaaaatttttttaaaaatttcaagaaggaaagcaaaatcctctctgaagaaaccatcactttgattgaatcccatctggagaataagaacctcaaagaggcactgactgtgattagtcatgctctgcgaaatattgacaaagccccactgaacattgctgtgactggagaaacaggaacagggaaatccagctttatcaatgccttgagaggaataagtagtgaagaaaaagatgcagcccccactggggtaatagagacaaccatgaagagaactccatacccacacccaaagcttcccaatgtgacaatatgggacctgcctggcattgggtcaaccaacttcccaccacaaaactatctgacagaaatgaagtttggtgagtatgacttcttcattatcatctcagctacacgcttcaaagaaattgatgcacatctggccaaagccattgcaaagatgaacataaagttctactttgtccgaaccaagatagatcaagatatcagtaatgaacagaggagtaaacctaagtctttcaatagagacagtgtcttaaagaaaataaaagatgaatgcttgggtcttctccagaaggttctctccagtcagcctccaatcttcctagtctctaactttgatgtgtctgactttgacttcccaaagctggagaccaccctactgaaggaactcccagcccacaagcgccacctcttcatgatgtccttgcacagtgttactgagactaccattgcccggaagagggatttcctcagacagaagatctggctggaggccctgaaggctggactatgggccaccattccacttgggggcttagtcagagataaaatgcagaagttggaagagaccttgactctctacaggtcttactttgggctggatgaagcctcactagaaaatattgccaaggatttcaatgtgtctgtgaatgaaattaaggcacaccttaggtctctccagttgttaacaaagaacaatgacatgtccttcaaagaaaaactgttgaaatatattgaatatatttcctgtgttactgggggaccacttgcctcaggcctttactttagtaagacttactactggcaaagtctcttcattgatactgtggcaagtgatgccaagtctctccttaataaggaagagtttttgtcagagaagccaggatcgtgcctgtctgacctccctgaatactgggaaacaggaatggagctgtga

>Irgb2 1377 bp

atgggtcagacttcctcttctacatcccctccaaaggaggatcctcctttgacatttcaggtgaaaaccaaagtcctgtctcaggaattaatcgcctccattgaatcatccctagaggatggaaaccttcaggaaacagtttctgcaatcagcagtgctctgggtgacatagagaaagtcccactgaacattgcagtgatgggggagactggggctggaaagtccagtctcatcaatgctctgcagggagtgggggatgatgaagaaggtgcagctgcttccacaggggtggtacatacaaccactgagagaacaccatacacttacacaaagtttcccagtgtgacactgtgggacctgcctagcattggatctactgccttccaaccacatgattatctgaagaaaatcgagtttgaggagtatgacttcttcattattgtctctgctatacgtattaaacagagtgatatagaacttgccaaagctattgtgcaaatgaatagaggtttgtactttgttcgaacaaagacagattctgatctagagaatgaaaaactgtgtaaccctatgagattcaatagagagaacatattgaagagtattcgaatttgcctttcaagtaatctcaaagaacgtttccagcaggagcctccagtcttcttagtctctaactttgatgtgtctgactttgacttcccaaagctggaatccacccttctcagccagctcccagcctacaaacaccaaatattcatgagtactttgcaagttgttataaatgccatagtagaccggaagagggatatgctgaaacaaaagatctggaaggaatccataatgccaagagcatgggctaccataccatcccggggactgacccaaaaggacatggagatgttgcaacagactttgaatgactatagatcttcctttggcctgaatgaggcatcactggaaaacatcgctgaggatttgaacgtgacactggaggagctcaaggcaaacattaagtctccacatttgttctcagatgagccagatacatccttaacagagaaactattgaagtacattggaaatccttacttttcaaaggttttccacttgcaaaattatttcatagacacggttgcgagtgatgctaaaattatcctcagtaaagaagagcttttcacagagcaggtgagctcattcaactctaaggcctctccatatcgggaggagtccgtgggcaaagtgtttccagttagtccaggaagcacctttctatttcacttctttgagatgtttcaaagtgactctgacaaactttgtcatgtccatgttctgctcctgttgacatcctggggcctctctggtgagactgttacatga

>Irgb3 1266 bp

atggctcaacttttggtgttcagctttgaaaacttttttaagaatttcaagaaggaaagcaaaatcctctctgaggaaaccatcaccttgattgaatcccatctggaggataagaaccttcagggggcattgtctgaaattagtcatgctctgagcaacattgacaaagccccactgaacattgctgtgactggagaaacagggacaggaaaatccagctttatcaatgccttgaggggagtgagggatgaagaagaaggtgcagcacccactggggtggtagagacaaccatgaagagaactccatacccacacccaaagcttcccaatgtgacaatatgggacttgcctggcattgggtctactaccttcccaccacaaaactatctgacagaaatgaagtttggtgagtatgacttcttcattatcatctcggctacatgcttcaaagaaattgatgcacatctggccaaaactattgaaaagatgaacacaaagttctactttgtccgaaccaagatagatcaagatgtcagtaatgaacagaggagtaaacctaggtctttcaatagagacagtgtgctaaagaaaataagagatgactgctcaggtcatctccagaaggctctctccagtcagcctccagtcttcctagtctctaactttgatgtgtctgactttgacttcccaaagctggagaccaccctactgagggaactcccagcccacaagcgccacctcttcatgatgtccttgcacagtgttactgagactgccattgcccggaagagggatttcctcagacagaagatctggctggaggccctgaaggctggagtatgggccaccattccacttgggggcttagtcagaaataaaatgcagaagttggaagagaccttgactctctacaggtcttactttgggctggatgaagcttcactggaaaacattgccaaggatttcaatgtgtctgtgaatgaaattaaggcacaccttaggtctctccagttgttaacaaagaacaatgacatgtctttcaaagaaaaactgttgaaatatattgaatatatttcctgtgttactgggggaccacttgcctcaggcctttactttagaaagacttactactggcaaagtctcttcattgatactgtggcaagtgatgccaagtctctccttaataaggaagagtttttgtcagagaagccaggatcatgcctctctgacctccctgaatactgggaaacaggaatggagctgtga

>Irgb4 1326 bp

cagcatcctccactgcacacagctacatgccagccttcctctagcagaccctctcgtttaactgctcaacttttggtgttcagctttgaaaacttttttaagaatttcaagaaggaaagcaaaatcctctctgaggaaaccatcaccttgattgaatcccatctggaggataagaaccttcagggggcattgtctgaaattagtcatgctctgagcaacattgacaaagccccactgaacattgctgtgactggagaaacagggacaggaaaatccagctttatcaatgccttgaggggagtgagggatgaagaagaaggtgcagcacccactggggtggtagagacaaccatgaagagaactccatacccacacccaaagcttcccaatgtgacaatatgggacttgcctggcattgggtctactaccttcccaccacaaaactatctgacagaaatgaagtttggtgagtatgacttcttcattatcatctcggctacatgcttcaaagaaattgatgcacatctggccaaaactattgaaaagatgaacacaaagttctactttgtccgaaccaagatagatcaagatgtcagtaatgaacagaggagtaaacctaggtctttcaatagagacagtgtgctaaagaaaataagagatgactgctcaggtcatctccagaaggctctctccagtcagcctccagtcttcctagtctctaactttgatgtgtctgactttgacttcccaaagctggagaccaccctactgagggaactcccagcccacaagcgccacctcttcatgatgtccttgcacagtgttactgagactgccattgcccggaagagggatttcctcagacagaagatctggctggaggccctgaaggctggagtatgggccaccattccacttgggggcttagtcagaaataaaatgcagaagttggaagagaccttgactctctacaggtcttactttgggctggatgaagcttcactggaaaacattgccaaggatttcaatgtgtctgtgaatgaaattaaggcacaccttaggtctctccagttgttaacaaagaacaatgacatgtctttcaaagaaaaactgttgaaatatattgaatatatttcctgtgttactgggggaccacttgcctcaggcctttactttagaaagacttactactggcaaagtctcttcattgatactgtggcaagtgatgccaagtctctccttaataaggaagagtttttgtcagagaagccaggatcatgcctctctgacctccctgaatactgggaaacaggaatggagctgtga

>Irgb5 1404 bp

atgggtcagacttcctcttctacaccccctccaaaagaggatcctgatttgacctccagctttggcacaaatcttcagaatttcaagatgaaaaccaaaatcctgtctcaggaattaattgccttcattgaatcatccctagaggatggaaaccttcaggaaacagtttctgcaatcagcagtgctctgggtggcatagagaaagccccactgaacattgcagtgatgggggagactggggctggaaagtccagtctcatcaatgctctccagggagtgggggatgatgaagaaggtgcagctgcttccacaggggtggtacatacaaccactgagagaacaccatacacttacacaaagtttcccagtgtgacactgtgggacctgcctggcattggatctactgccttccaaccacatgattatctgaagaaaatcgagtttgaggagtatgacttcttcattattgtctcttccggacgctttaaacataatgacgcagaacttgccaaagccattgtgcaaatgaataggagtttctattttgttcgaacccacacagatcttgatttaatggttgtaaaacggagtaatcctaggagattcaacagagagaacacattaaagcagattcgacataccatttcaagtatgcttaaggaagttacccaccaggagcctccagtcttcttagtctctaactttgatgtgtctgactttgacttcccaaagctggaatccacccttctgagccagctcccagcctacaaacaccacatgttcatgctcactttgcctattgttacagattccaccatagaccggaagagggatatgctgaaacaaaaggtctggaaggaatccacaatgccaagagcatgggctaccataccatccctgggactgacccaaaaggacatggagatgttgcaacagactttgaatgactatagatcttcctttggcctggatgaggcatcactggaaaacatcgctgaggatttgaacgtgacactggaggagctcaaggcaaacattaagtctccacatttgctctcagatgagccagatacatccttaacggagaaactattgaagtacattggaaatccttacttttcaaaggttttccatttgcaaaattatttcattgacacggttgcgagcgatgttaaaattatcctcagtaaagaagagcttttcacagagcaggtgagctcattcaactctaaggcctctctatatcgggaggagtccgtgggcaaagtgtttccagttggtccaggaagcacctttctatttcatttcattgaaatgtttcaaagtgactctgacgaactttgtcatgtccatgttctgctcctgttgacatccgggggcctctctagtgagactgttacatga

>Irgb6 1248 bp

atggcttgggcctccagctttgatgcattctttaagaattttaaaagggaaagcaaaatcatctctgaatatgacatcaccttgattatgacttacatagaggaaaataagctacagaaagctgtttctgtaattgaaaaggtactgagagacatcgagagtgctcctctgcacatagctgtgacaggggaaacaggcgcagggaagtccactttcatcaataccctgaggggggtggggcatgaagaaaaaggtgcagcccccactggcgcaatagagacaaccatgaagagaactccatacccacacccaaagcttcccaacgtgacaatatgggacctgcctggcattgggaccactaacttcacaccacaaaactatctgacagaaatgaagtttggtgagtatgacttcttcattatcatctcagctacacgtttcaaagaaaatgatgcacaactggccaaagccattgcacagatggggatgaatttctactttgtcagaaccaagatagacagcgacttagataatgaacagaagtttaagcctaagagtttcaataaggaggaagtcctcaagaatattaaggattactgctctaatcatcttcaggagtctctcgacagtgagcctccagtcttcctagtctctaacgttgatatatcgaagtatgacttcccaaagctggaaactaaactcctacaggatctcccagcccacaagcgtcacgtcttctcactgtctttgcaaagtcttactgaggccaccattaactacaagagagattccctgaagcaaaaagtcttcctagaagccatgaaggctggagcattagccaccattccacttggtggcatgatcagtgatatcttagagaatctggatgaaacattcaatctctacaggtcttactttgggctggatgatgcttcactggaaaacattgcccaggatttgaacatgtctgtggatgacttcaaggtacaccttcgatttccccatttgtttgcagaacacaatgatgagtccttagaagacaagctatttaaatatatcaaacacatttcttcagttactggtgggccagtcgctgcagtcacttactatcgcatggcttattatttgcagaatctctttcttgatactgcagccaatgatgccatagctcttctgaatagtaaagcactttttgagaagaaggtgggaccatatatatctgagccccccgagtactgggaagcttga

>Irgb7 1419 bp

cctttttggtttgttcctcccttaggtaccattgacatttgccaagattgggtcaaacttcctcttctacatcccctccaaaggaggatcctgcttttgaccttccagatgaaaaccaaaatcctgtctcaggaattaatcaccttcattgaattgtacctagaagatggaaacctttaggaaacagtttctgcaatcagcagtgctctgggtgacatagagaaagtcccactgaacattgcagtgatgggggagactggggctggaaagtccagtctcatcaatgctctccagggaacaggagctgatgaagacggtgtgactgctcctgttggggtggtatatacaaccattgagaaaaaatcatacccatacgcaaagtttcccagtgcaatactatgggaactgcctgccattgggtttcatcacttccaaccacatgattatctgaagaaaatcaagtttgaggagtatgacttcattattgtctctgctggacgcattaaacatagtgatgtagaacttgccaaagctattgtgcaaatgaatagaggtttgtactttaatcgaacaaagacagatattgatctaaagaatgaaaaactgtataaccctatgagattcaatagagagaacacattgaagagtcttcaaatttgcatttcaagtaatctcaaagaatgtttccaccaggagcctccagtcttcttagtctctaactttgatgtgtctgactttgacttcccaaagctggaatccacccttctcagccagctcccagcctacaaacaccaaatattcatgagaactttgcaagttgttataaatgccatagtagactggaagagggatatgctgaaacaaaaggtctggaaggaatccacaactccaagagcatgggctaccataccatccctgggactgacccaaaaggacatggagatgttgcaacagactttgaatgactatagatcctcctttggcctggatgaggcatcactgaaaaacatcgctgaggatttgaacgtgacactggaggagctcaaggcaaacattaagtctccacatttgctctcagatgagccagatacatccttaacagagaaactattgaagtacattggaaatccctacttttcaaaggttttccacttgcaaaattatttcattgacacggttgcaagcgatgttaaaattatcctcagtaaagaagagcttttcacagagcaggtgagctcattcaactctaaggcctctccatatcgggaggagtccgtgggggaagtgtttccagttggtccaggaagcacctttctatttcacttctttgagatgtttcaaagtgactctgacaaactttgtcatgtccatgttctgctcctgttgacatcctggggtctctctggtgagactgttaca

>Irgb8 1263 bp

atggctcaacttttggtgatcagctttgaaaatttttttaagaatttcaagaaggaaagcaaaatcctctctgaggaaaccatcaccttgattgaatcccatctggaggataagaaccttcagggggcattgtctgaaattagtcatgctctgagcaacattgacaaagccccactgaacattgctgtgactggagaaacagggacagggaaatccagcttcatcaatgccttgaggggagtaaggggtgaagaagaaggtgcagcgcccactggggtggtagagacaaccatgaagagaactccatacccacacccaaagcttcccaatgtgacaatatgggacctacctggcattgggtcaaccaacttccaaccacaaaactatctgacagaaatgaagttcggtgagtatgacttcttcattatcatctcggctacacgcttcaaagaaattgatgcacatctggccaaagccattgcaaagatgaacacaaagttctactttgtccgaaccaagatagatcaagatgtcagtaatgaacagaggagtaaacctaagtctttcaatagagacagtgtcttaaagaaaataagagatgactgctcaggtcatctccagaaggttctctccagtcagcctccagtgttcctagtctctaactttgatgtgtctgactttgacttcccaaagctggagaacaccctactgagggaactcccagcccacaagcgccacctcttcatgatgtccttgcacagtgttactgagactgccattgaccggaagagggatttcctcagacagaggatctggctggaggccctgaaggctggagtatggaccaccattccacttgggggcttagtcagagataaaatgcagaagttggaagagaccttgactctctacaggtcttactttgggctggatgaagcctcactagaaaatattgccaaggatttcaatgtgtctgtgaatgaaattaaggcacaccttaggtctctccagttgttaacaaagaacaatgacatgtccttcaaagaaaaactgttgaaatatattgaatatatttcctgtgttactgggggaccacttgcctcaggcctttactttagaaagacttactactggcaaagtctcttcattgatactgtggcaagtgatgccaagtctctccttaataaggaagagtttttgtcagagaaaccaggatcgtgcctctctgacctccctgaatactgggaaacaggaatggagttg

>Irgb9 1404 bp

atgggtcagacttcctcttctacactccctccaaaggacgatcctgatttcatcgccagctttggcacaaatcttcagaatttcaagatgaaaaccaaaatcctgtctcaggaattaattgccttcattgaatcatccctagaggatggaaaccttcgggaaacagtttctgcaatcagcagtgctctgggtggcatagagaaagccccactgaacattgcagtgatgggggagactggggctggaaagtccagtctcatcaatgctctccagggagtgggggatgatgaagaaggtgcagctgcttccacaggggtggtacatacaaccactgagagaacaccatacacttacacaaagtttcccagtgtgacactgtgggacctgcctggcattggatctactgccttccaaccacatgattatctgaagaaaatcgagtttgaggagtatgacttcttcattattgtctcttccggacgctttaaacataatgacgcagagcttgccaaagccattgtgcaaatgaataggagtttctattttgttcgaacccacacagatcttgatttaatggttgtaaaactgagtgatcctaggaaattcaataaagagaacatcttagagcagattcgaaattctatttcaaatatacttaaggaggttacccaccaagaacctccagtcttcttagtttctaactttgatgtatctgactttgacttcccaaatctggaatccacccttctgagccagctcccagcctacaaacaccacatgttcatgctcacgttgcctattgttacagattccaccatagaccggaagagggatatgctgaaacaaaagatctggaaggaatccataatgccaagagcatgggctaccataccatcccggggactgacccaaaaggacatggagatgttgcaacagactttgaatgactatagatcttcctttggcctggatgaggcatcactggaaaacatcgctgaggatttgaacgtgacactggaggagctcaaggcaaacattaagtctccacatttgctctcagatgagccagatacatccttaacagagaaactattgaagtacattggaaatccttacttttcaaaggttttccacttgcaaaattatttcattgacacggttgcaagcgatgttaaaattatcctcagtaaagaagagcttttcacagagcaggtgagctcattcaactctaaggcctctccatattgggaggagtccgtgggcaaagtgtttccagttggtccaggaagcacctttctatttcacttctttgagatgtttcaaagtgactctgacaaactttgtcatgtccatgttctgctcctgttgacatcctggggcctctctggtgagactgttacatga

>Irgb10 699 bp

atgggtcagtcttcttctaaacccgatgcaaaggctcacaatatggcctccagccttactgaattcttcaagaatttcaagatggaaagcaaaatcatttctaaggagaccatcgattcaattcagtcatgtatccaagaaggagacatccagaaggtgatttctataatcaatgctgccttgacagacattgagaaagcccccctgaacatcgctgtgacaggggagacaggggcagggaagtccacgttcattaatgcccttcggggaataggacatgaggaaagtgagtcagctgagagtggagcggtggagacaaccaaggacaggaagaaatacacccaccctaagttccctaatgtgaccatctgggatctccctggggttgggacaactaacttcaaaccagaagaatatctgaagaaaatgaagttccaggagtatgattttttcctgatcatctcatctgctcgctttagagataatgaagcccagctggctgaagcaatcaaaaaaatgaaaaagaagttctactttgttcggaccaaaattgacagtgatttgtggaatgagaagaaagctaaacccagttcctacaatagggagaaaatcctggaggtgatccgaagtgactgtgtaaagaatctgcagaatgctaacgcagcctctactcgtggctttcttagtctcaagctttga

>Irgc 1392 bp

atggcaacttccaggttgcccgccgtgcctgaggagaccaccatcctcatggccaaggaagagctggaggccctgcgcactgcttttgagtctggcgacatccctcaagccgcctctcgccttcgggagctgctggccaactcagagaccacccggctggaagtgggcgtcacgggtgagtcgggagccggcaagtcctccctcatcaatgccctacgcggcctgggggccgaggatcctggcgcagctctcactggggtcgtggagaccaccatgcagccttcgccctacccgcacccgcagtttcccgacgtgaccctgtgggacctgccgggggccggttctccaggctgctcagcagacaagtatctgaagcaggtggatttcggccgctatgacttcttcttgctcgtctccccccgtcgctgtggcgccgtggagtcccgcctcgcttctgagatcctgcgccaggggaagaagttttactttgtgcgcaccaaggtggacgaggatctggcggccacccgcagccagaggccctcgggtttcagcgaggctgcagtcctccaggagatccgagatcactgcacggagcggctgcgggtagcgggtgtgaatgatccccgcatcttcctggtgtccaacctgtcgccaacccgctatgacttcccgatgctcgtgaccacctgggagcacgacctgcccgcccaccgtcgccacgccggtctgctgtccctgcctgacatctcgctggaggctctgcagaagaagaaggacatgctacaagagcaggtgcttaagactgccttggtatctggtgtcatccaggccctgccggtccccggactggcagccgcctacgacgacgccttgcttatccgctcactgcggggctaccaccgcagcttcggcctagacgacgactcgctggccaagctggccgagcaggtgggcaaacaggcaggggacctgcgctccgtcatccgctcccccctggccaacgaggtctcaccagagactgtcctgagactctactcgcagtcctcagatggtgccatgcgggtggcccgtgcctttgagaggggcatccctgttttcggcacgctggtggccgggggtatcagcttcggcacggtctacaccatgctccagggctgtctcaatgagatggctgaggacgcccaacgcgtccgcatcaaagccctggaggaagatgagccccaggggggtgaggtgagcttggaggcggctggtgacaatttagtggaaaagcggagcactggggaaggaaccagcgaggaagccccgctgtccacccgcaggaagctcggcctcctcctcaagtatattcttgacagctggaagaggcgcgacttgtcagaagacaaataa

>Irgd 1263 bp

atggatcagttcatctcagccttcctgaagggtgcttcagaaaatagtttccaacaattagctaaggagtttctgcctcagtactctgcattaatcagtaaggcaggaggcatgctctctccagaaaccctcactggtattcacaaagccctccaggagggaaatctctctgatgtcatgatccagattcagaaagcaattagtgctgcagagaatgctatcctggaggtggctgtgatcgggcagtctgggactggcaagtccagttttataaatgcccttcgagggctgggccacgaagcagatgaatccgctgatgttgggactgtggagaccactatgtgtaaaaccccctatcaacatccaaaatatcccaaagtgatcttctgggacctgcctgggactgggacacccaatttccacgcagacgcttatctagaccaagtgggatttgccaactatgacttcttcatcatcatttcttcttcccgcttcagcctcaatgatgctctcctggctcagaaaataaaggatgctgggaagaagttctactttgttagaaccaaggtggatagtgacttatataatgaacagaaagccaaacccatagctttcaagaaggagaaagtccttcagcagattcgagactactgtgtgactaatctcatcaaaactggggtgactgaaccatgcatcttcctgatctccaacttggatctgggtgcgtttgatttccccaagctggaggagactctgctaaaggagctccctgggcacaagcgtcatatgtttgccctgctcttgcccaatatctcggatgcttccattgagctaaagaaacattttcttcgggagaagatctggctggaggccttgaagtcagcagctgtgagcttcatccccttcatgactttctttaagggctttgatttgcctgaacaagaacagtgcttgaaggattaccgaagctattttggcttggatgatcaatcgattaaagagattgctgaaaaattgggcgcacccttggcagacatcaagggggaacttaagtgcttggatttctggtcccttgtaaaggataacagcataatagcgcaagctacgagtgctgctgaagctttttgtgctgtgaaaggaggccctgaatcttctgccttccaggctttaaaagtctattataggcgcacacagttccttaacatagtggttgacgatgctaaacatctcttgagaaagatagaaacagtaaatgttgcctag

>Irgm1 1230 bp

atgaaaccatcacacagttcctgcgaggctgctccactactccccaacatggcagagacccattatgctcccctgagctcagccttcccctttgtcacgtcataccaaacaggctccagcaggttacctgaggtcagtaggagcaccgaaagagctttaagagaaggaaaactactggaactggtctacggaatcaaggagactgtggcaacattgtcccagattccagtgagcatctttgtgactggggactctggcaatggcatgtcatctttcatcaatgcacttcgagtcatcggccatgatgaagatgcctcggctcccactggggtggtgaggaccacgaagacgcggactgagtactcttcatcccactttcccaatgtggtgctgtgggacttacctggattgggggccacagcccaaaccgtagaggactatgtggaagagatgaaatttagcacatgtgacttattcatcatcattgcctctgagcagttcagctcgaatcatgtgaagctgtccaaaattatccagagcatgggaaagaggttctatattgtctggaccaagctggacagggacctcagcaccagtgtcctatcagaggtccggctcctacagaatatccaggagaatatccgagagaatctgcagaaggagaaagtgaagtacccccccgtgttcctggtatccagtctagaccctttactatatgacttcccgaagcttagggacacacttcataaagatctctccaacatcaggtgctgtgaacccttaaagaccctttatggcacttatgagaagatcgttggtgataaagtagcagtctggaagcagagaatagccaacgagtccttgaagaattctctcggtgtcagagatgatgacaacatgggcgagtgtctgaaagtgtaccgactgatatttggtgtagatgacgaatcagttcagcaggtagcccagagtatggggacagtagtcatggagtacaaggacaacatgaagtcccaaaacttttatactctccgcagagaggactggaaactgaggctgatgacatgtgcaattgtgaatgcattcttccgtttgttgagatttctcccatgcgtatgctgctgtttaagacgcttgagacataaacgcatgcttttcttagttgcccaggacaccaagaacatcctagagaaaatcctgagggactccatcttccctccgcagatctag

>Irgm2 1224 bp

atgcctacatccagggttgccccgttgctcgacaacatggaagaggcagttgagtcacctgaggttaaagaatttgaatacttctccgacgctgtattcattcccaaagacggcaatactttatctgtaggcgtcattaagagaattgagactgccgtgaaagaaggggaggtagtgaaagtggtttctatagttaaagagatcatacagaacgtttccagaaacaaaataaagatcgctgtgactggggactctggcaatggcatgtcatctttcatcaatgcccttaggctcatcggacacgaagagaaagattcagctcccactggggtggtgagaaccacccagaaaccaacctgttacttttcctcccactttccctatgtggagctgtgggacctgcctggcttaggggccacagcccagagtgtggagagctacctggaagagatgcagatcagcatatacgaccttatcatcatcgtagcttctgagcagttcagcttaaatcatgtgaagctggccataaccatgcagaggatgagaaagaggttctatgtcgtctggaccaagctggacagggacctcagcacaagtaccttccctgaaccccagctactgcagagtatccaaaggaatatccgggatagtcttcagaaggagaaagtgaaggagcaccccatgttcctggtatctgtctttaagcctgaatcacatgacttcccaaagcttagggagacactacaaaaagacctccctgtcatcaagtaccacggcctcgtcgaaaccctttaccaagtctgtgagaaaactgttaatgagagagtagagtccattaaaaagagtatagatgaagataacctacacacagagtttgggatctcggatccgggtaacgcgatagagattcggaaagccttccaaaaaacctttggtttggatgacatatctctccacctggttgctctggaaatgaaaaataaacatttcaacactagtatggagtcccaggagacccagaggtaccaacaagatgactgggtgctggctcggttgtatcgcactggaacccgggttggctctataggttttgactacatgaagtgctgctttacctctcatcacagtcgatgcaaacaacagaaagatatacttgatgaaactgctgccaaagccaaggaagttctgttaaaaatcctgagactctccattcctcatccttag

>Irgm3 1345 bp

atggatttagtcacaaagttgccacaaaatatctggaagacttttacacttttcataaacatggcaaattatctcaagcgcctcatcagcccgtggtctaaatctatgactgctggtgagtcactttattccagccagaactcatcctctccagaagtcattgaagatattggtaaggcggtgacagagggaaatttacagaaagtgataggtatagtcaaagatgaaattcagagtaagtcaagatacagagtaaagattgctgtgactggggactctggcaatggcatgtcatctttcatcaatgcccttaggttcattggacatgaggaggaggattcagctcccactggggtggtgaggaccactaagaaaccagcctgctactcctctgactcccactttccctatgtggagctgtgggacctgcctggcttaggggccacagcccagagtgtggagagctacctggaggagatgcagatcagcacatttgaccttatcatcatcgtagcttctgagcagttcagctcaaatcatgtgaagctggccataaccatgcagaggatgagaaagaggttctatgtcgtctggaccaagctggacagggacctcagcacaagtaccttccctgaaccccagctactgcagagtatccaaaggaatatccgggagaatctccagcaggctcaggtgagggacccccccctattcctgatatcctgttttagcccgtcttttcacgacttcccagagcttagaaacacactgcaaaaagacatcttcagcatcaggtacagagatcccttagagatcatttctcaagtctgcgacaagtgcatcagcaataaggcgttctctctgaaggaagatcagatgctcatgaaagacctggaggcagctgtcagctccgaggatgacactgccaacctggagaggggtctccagacctaccagaagctctttggtgtggatgatgggtcacttcagcaggtagctcggagtacagggagactggagatgggctccagggctctgcagttccaggacttgatcaagatggacaggagactggagctgatgatgtgttttgccgtgaacaagttcctcaggcttctcgaaagctcatggtggtatggcttgtggaacgtcgtcacccgctacttcagacaccagaggcacaagctcgtcattgaaatagtggctgagaacaccaagacctccctgaggaaagctctaaaggactctgtcctccctcccgaaattcactga

>irgq 1224 bp

aggctcctgcctcctgcccaggatggcttcgaggtgttgggcgcagccgagctggaagctgttcgtgaggccttcgagaccggtggcctggaggcggcgctgtcgtgggtgcgcgctggtctggagcgcctgggcagcgcacgactggacttggcggtggccggcaccaccaatgtaggccttgtgctagatatgctgctggggttggatcctggagacccaggtgctgcgcctgcctcagcacccactgggcccaccccttatcctgctccagagcgccccaacgtagtgctctggaccgttcccctgggccccacggccacatctcctgccgtcacccctcacccaacccactacgatgccctgatccttgtcacccccggggctcccacagaggagaactgggcccaggtccgctcattggtgtccccagatgctccactcgtcggtgtgcgcacggacggccagggcgaagatccacccgaggttctggaagaagaaaaggcccagaatgcaagtgatgggaactcaggggatgcacgcagcgaaggaaagaaagctggcatcggggactcggggtgcactgccgctcggagcccggaggatgagctatgggaggtgctggaggaggcgcctccgcccgtgttcccgatgcggcccggcggtctcccaggcctcggaacctggctccagcacgcactgcccacagctcaggccggagctctgctgctggcgctgccacctgcaagtccccgggcggcgcggaggaaggcggctgcgctacgggcaggggcgtggaggccagccctgttggctagcctggcggcggccgccgccccagtaccagggctgggctgggcttgcgatgtggcgcttctccggggacagctggccgagtggaggcgcgcgctaggcctcgaacccgcggccgtagcacgacgtgagcgcgccttgggcctggctcccggagtactggcaacgcgcacgcgcttcccgggcccggtgacgcgagccgaggtagaggccaggctggggtcctgggcaggtgagggcacggcgggaggtgcagcgttgagcgcgctctccttcctatggcccacgggtggtgctgcggcaacaggtgggctgggttaccgtgctgcgcacggtgtacttctacaagccttagatgagatgctggctgatgcggaggcggtgctgggacccccagagcccaaccaatga

**NUCLEOTIDE SEQUENCES FOR ZEBRAFISH IRG GENES**

>irge1 1233 bp

atgcctgagaaggaggaggataaaaatgaaaatctttatataatttctagtgagtttctagacataatgtcgaatgccacagatgatccagactcaatcagtgaagatatgaaggaagtaattgacgctaagcctaaagaaaaaacaaggaaactaaaagataaactgacagagttggagaatgttactcttaacatggcaataacaggcatgacaggagcagggaaatcttcatttgtcaatgccttgcgaggccttcgtgatgatgatgaaggagcagcttccactggaacaaccgagaccaccatgaagccaaacatgtatgagcatccctttatgcccaatgtgaagatctgggacctgcctggaatcggaagtccaaaattcagagcaaagaaatacctaaaagacgtcaattttcacatgtatgacttttttctcatagtgacttcagaaaggtttagagagaatgacattgagctggccaaagccatcaataagagcaacaagctattttatttcatacgtaccaaaattgacaatgacgttcgtgctgagtccaacaaaagaaacttcgatgagcgtgtgctgcttgataaaatccgagaggactgtaaggtgaaccttctcaaattgaatatttccaaaatattcctaatatcttcatttcatttggagagatatgactttcagaagctggtcaacacccttgaggaggaacttcccaaaaacaagagatttgctctcatacagtctttgcctgtttattcacttgagaccctcacaaagaagataacctacttcaagaaactcatttggctgaatgctgttggggccggagttggagcgtttcctccaatcccaggagtgtcactggccgttgattacggcataatgaagaagttctttaagcaagtcttcatggcattcggcctgtcaaatcaggcactgcaggtgctgtcagaaagggtgaataaaccagtggaggttctgaatgccgccaagacttcacgctttaaagatggagtcactgaccgtattctgatagacatgatgtctaatccagtcattgctatcactaagactctgggaaccataatggctctgctgcctggaggagctctgcccgcagggggtgcggctgttgcctctgtgcactacttgctgaatgtaggactcaaggagatggcagatgacaccaggaaagttcttgtcgtgtcacaacttgcctaa

>irge2 1233 bp

atgaagatacagaagcagaagcaggaattgtccaattccagtaaaccagatacacattctcacagtactgctaaagaaaatgtgagtttaaaatcagcaaacactgtacaagttgaacatatatatgaaatgcctgatgttcatctcaatagttctgctgaatacattaatgaaatggaatgtgtgattgagcaaaacaagcaactgggcaacgtcacgcttcatgttgcagtaacaggttcaactggagcaggaaaatccagcttcatcaatgcaatacgtggtcttacatccgatgatgaaaatgcagctccaacaggtgtgactgagacgacgttggtgccaacgatgtacagacatcctacaatgccaaatattgaattgtgggatctgccagggactggaagtcctaaatttaaagccaagaaatacctaaaagatgtgaaacttgagaccttcgatttctttatcatcatttcctcagaaagattcaaggaaaatgacataatgctggcaaatgcgattaaagagaggaagaagctgttttatttcctccgctcaaagatcgacaatgacattcatgctgaatctcataggaaagactttgatgagcagaaagtgctctcacacattcgtgagaactgtcacagaaacctgaaggatatagatgacccgcatgctttcctaatatgttcctttgaactgcacaaatatgattttcagacatttgtcgacacattggagaagcagctgcctgatcacaagagagatgctttgattttgtccttgccaatatactcctcaaagattttggaagagaaaattgaaatatttatgaagcaaacctggtcggcagctgttgcttctggctctgtggctgtggttccagtgcctggtctttccatggcttgtgatgctgccattttgctgggtttctttaccaagtgttactatgcatttggtctggacgaaaaatcaatagataagctgtctgtgagggtcaacaacctatctctgaaagctataagaaggtcacctctagtggtcgctattgggcaaaagaaactcaccaacaaggagctgtcggcactaactagcaaagaggcagctgttaagtttgcatggagcatggttcctgttgtagggagtataaaaacagcacaaatgtcttactctacaacactgaaccttctgcgaacaggagttcaggatcttgctgaaacagccagttga

>irge3 1161 bp

atggaaactcaggaccctgctattgctgaggcagtgcaggcctctggtgaatcaaccttggaaaaggccacagcaaaagccaaagaaagttttgaccagtttatgaatgtctcgcttaacatcgctgtgactggaaagacaggatcagggaaatcctcctttataaatgcactaagaggtctaaaggatgatgatgaaggagcagcacctactggggtcacagaaactacaatggagcccaacatgtatgagcatcctgcaatgccaaatgtgaagatctgggacctgcctggaataggaagtccaaacttcaaagcagataaataccttaaagatgtcaaattaaaaaattatgacttcttcattattttgaactccgagaggttcatgcagaatgatgtcatgttggctaaagaaataagaaagcagaagaaaaacttttactttgttcgctcaaagattgacaacgatatttcagcagagcaaagaaaaaaaacatttgatgaacagagggtcctttgcacaataagggaggactgtctgaaaaacttaaagcaactgggagaccccaaagtgttcctgatttcatcttttgacttggagaagtatgattttgaagaacttcaaaacacactagcagaagagcttccagtccataagagaaatgctctcctacaagcctggccggtgtgctctgctgcatctttggagatgaagatcaagatgtttgaaggtgtgatctgggctgcatctcttgcatctgctggtatagctgtggtccctctgcctggtctatcagcagcatgtgatacagggatggtggctcttttccttacaaggtgttactttgcatttggcttggatgatggatcactggcgaggctttcagaaaaaataaacaagcccttggtgggacatctggctaaatcaaagattgcgtctgccatccaagaaaaagcactcaccagattacaagtatctggtacactggttgtccttttctctgctgaatatgtagcaagtctcgttccaggtgttggcagtgtcgctgctgctggattgtcctttggtactacttattacctcttgagaagtggattaaaggaactggccaatgttgctcgagaaatcagaaaagaggtgctggacagtgttcgatag

>irge4 1155 bp

atgacagatgattcttctgctgacatgaatttctctggagctttgcagagattgggtgaaagtgacccaaatgcagctgctgtgaaggctaaagaagaactggaccgtcttgacagtgtgactctgaatatcgctgtgactggggaagcaggagcaggaaagtcttccttcattaatgcgcttcgggatctgagtgatgaagatgaaaactccgcaccaactggactgactgaaaccacaaagaaagctaccatgtacacccatcccacaaagccaaatgttagactgtgggacctgccaggcattggcacgccaaatttcaaggcaaaccaatacctcaaagatgtcaaatttgaaacgtatgatttttttatcatcatttcctcagagaggttcaaggagaatgacgtgtatctggccaaggagattcaaaaaaagcagaagaggttttattttgttcgaaacaagattgataatgacatttgctcagtagcgaatggaaaaatcaacgagcagcagctgctttgcgcaatcagggaagactgctacagaaatctgaaggaagtaggaaatcccaaagtgttcttgatttcctcttttgacttgaggaagtatgactttaatctggttggtactctagagtcagagctttcagatcaaaagggatttgctctggttcagtccgtgcctgtgtactctctggctatgctagagaaaaagaaagcattgttagagaagttcatttggctcgcagcattggcctcaagtgcatgtaccttagttcctaaccaattcatatctttgatcacagacaaggcgatactgatagtgtatctcataggctgtcattacgctttgggtttgaatgagaaatcgctcaaacagctgtcagagagaacaaacaagcctgtctccctgctgaaattggctataaagtcacctgtgtcactggcagtgctagacagaatgaggattagtcctatggcaaaaccagtgaaaagtttagaggatctgcttgatagtaaaaatcttgcagtcaatgttcagaacacagctgatgccttcaggaatagtcacaccaacctgactagagctctaaatgaaatgatcaaagacatgagacaagttcttcaagtggcaggtttggacgaataa

>irge5 1227 bp

aaggaggaggaggatgaaaatgaaaatctttatatagtttctagtgaattcataaacataatgtcgaatgccacagatgatccagactcaatcagtgtagatatgaaggaagtaattgacgctaagcctaatgaaaaaacaacaaaactaaaagataaactgacagagttggagaatgttactcttaacatggctataactggcatgacaggagtaggaaagtcttcatttgtcaatgccctgcgaggccttcgtgatgatgataaagatgcagctttcacaggaacaactgagaccaccatgaagccaaacatgtatgagcatccctttatgcccaacgtgaagatctgggacctgcctggaatcggaagtccaaaattcagagcaaagaaatacctaaaagatgtcaatttccacatgtatgacttttttttcatagtgacctcagaaaggtttagagagaacgacattgagttggccaaagccatcaagaagagcaacaagctgttttatttcatacgcactaaaattgacaatgacgttcgtgctgagtcctacaaaagaaacttcgatgagcctatgctgcttgataaaatccgagaagactgtaaggtgaacctactgaaagtgcgtatttccaaaatattcctaatatcttcatttcatttggagagatatgactttcagaagctggtcaacacccttgaggaggaacttcccaaaaacaagagatttgctctcatacagtctttgcctgtttattccctcgaggccctcacaaagaagataacctacttcaagaaactcatttggctgaatgctgttggggccggagtaggtgcaattgctccaatcccaggagtgtcactggccgttgagtatgtcatcatgaagaagttctttaagcaagtcttcatggcatttggactatcaaatcaggcactggaggtactctcaggacgagtgaacaaacctgtgaaggttctgaaagccgccaagacatcacgcttcaaagatggaatcactgagcatattctgatggacatgatatctaatccagtcattgctattgccgtaactctgggaaccataatggctctgctgcccggaggagctctgcccgcaggaggcacggctgtcgcaactgtgcactacttgctgaatgtaggactcagggagatggcagatgacacaaggaaagttcttgccatctcacaacttgcctaa

>irge6 1095 bp

atggaatgtgtgattgagcaaaacaagcaactgggcaacgtcacgcttcatgttgctgtaacaggttcaactggagcaggaaaatccagcttcatcaatgcagtaagaggtcttacatctgatgatgaaaatgcagctcctacaggtgtgactgagacgacgttggtgccaatgatgtacaaacatcctacaatgccaaatgttgaattgtgggatctgccagggactggaagtcctaaatttaaagccaagaaatacctaaaagaggtgaaacttgagaccttcgacttctttatcatcatttcctcagaaagattcaaggaaaacgacatcatgctggctaatgcgattaaagagaggaagaagctgttttatttcctccgctcaaagatcgacaatgacattcatgctgaatctcataggaaagactttgatgagcagaaagtgctctcacacattcgtgaggactgtcacaggaatctgaaggacatggatgacccacatgttttcctaatatgttcctttgaactgcacaaatatgattttcagacatttgtcgacacattggagaagcagctgcctgatcacaagagagatgctttgattttgtccttgccaatatactcctcaaagattttggaagagaaaattgaaatatttatgaagcaaacctggtcggcagctgttgcttctggctctgtggctgtggttccagtgcctggtctttccatggcctgtgatgctgccattttgctgggtttcttcatcaagtgttactatgcatttggtctggacgaaaaatcaatagataagctgtctgtgagggtcaacaacccatctctgaaagctataagaaggtcacctctagtggtcgctattgggcaaaagaaactcaccaataaggagctgtcggcactgactagcaaagaggcagctgttaagtttgcatggagcatggttcctgttgtagggagtaaaaaaacagcacaaatgtcttactctacaacactgaaacttctgcgtacaggagttcaggatcttgctgaaacagccagagaagtgttgaaagctgcaggagtgactggtgtctattaa

>irgg1 753 bp

atgtttttttctagattatgcatgccagcaaaagtccaagaagatcatttaggcacaatcagagatgtatttgctggtgaaagccccgagacaataccccatcggcttatttctcttttggaggtgtttgaccgttttaagatagatattgctgtgactggagactctggtgcaggaaagtcttctttaatcaatgcgattcttggactgaaacctgatgacaaaggagctgctcaaactggagctatagaaaccacaaaacaagcaactatgtatcagcaatcaaatctgccccacatcaggttgtgggatcttccaggaatgggaaccccttcctttgcgtccaagagttatgtaaagatgatgaactttgacctttacgacatgtttatggtcgtgatctcagagagggttcgagaaaacaacatgctcttggttgatgaaattgataaacgtaagaagccattttattttattagaaccaaaatcgataacgatgtgaagtcccagcgaaggaaaagcaaattttctgaaacacaagcgttggaacaaatgagacaagactgtgagaaatatctgaaggagaagaaactggaccctcatatatttttagtttcaacccatgacacacataattatgagtttcagaagttcatcagcacctttaaagatgaggttttcaaaattagagctgaagaattttcaggttttctggacaagatgcttcatggtggatggctaaaagcaaggtga

>irgq1 744 bp

atgcttcatggtggatggctaaaagcaagatatgctacacagcacgtccagcagactgagaaacttgaaacggaagacatcacaaaattgcagaatatgtacaagagcacaggctttggagctgcaaaagtcagtgctgtgctggaggctttgagtcactttcagcttgatgttgcagttttgggtgagacgggatctggagtgtccactttggtaaatgcattagttggtctggagaatgaagagagcagtggagcaggagcatccatcagcaacccagcactgagtcctgtatatccagatgtccggttttgggatatttctggcatagaggctgtcatggattactcagtgtttgagatgaaacaagccatgaaatgttacgacttttatatcatcattgtttcagactgggagaaagtacgacatgtaaaactcgccaaggaagttgaaaaattaaggaagcattacctcttggtccagactaaagtcgactcttgtttacagactcagggagatttatgctgtgaagagactgagatactggatggtcttcgggcacaatttacacaagagcttcagagggaaaaactgtcagaacagcagatgtttctcatcaacagccaggacagaagtgcatttgattttgtaagtttggagagtgcactgtccagtgacctcaacactatcaggacaagtgcttttgcatattatatcgcaagaacagtaaaggaaaatttgtaa

>irgq2 1140 bp

atggctgacgtgataaagggtctcaacctgctggaaacactgaaagagtccatcgagaagaacaacatctccgatattcgagatgcactggaggacatgctaatcagcagaatcaacatcgccatcgctggggaaagaaatgcagaaaaagccactttcatcaactctctccgaggcctgagccaagaagatgaaggtgcagcccaaaatccaccatctgccgccccagaagaactagccgtattcaccaatcccaaacacccagatttccgcctctgggatcttccaccaatctctagcgatgctaatttcaaaccagaggattacattgagcgttttaaagctacacgctacaatgctatcattctcacatccaccgacagacctagcgccaacagcgttgcggtatggaaggaagttagatcacttcagaaagagactgtttactttgtattattagcatctgtgaaagacaccgagaaatcactggaggcgaaaaaggcggcaagtttagatgtccttaaagcagaaggtgttcctctgccaaaggtgtttctagtgcaaccgtccgccctagagaagctggactttctcacatttttagaggtcatgagaggagatcttccggagattcgagcccatgctcttcttctggcgcttcctacgttttcaagctcattggtcactcagaagaaagacgcctttaaggcattggtgtgggccgctgcttcattatctggtggtgtttctgctattccagtaccactggtgtcctcaatggtggatgctacagtgggcgtccggattttggttaaagctcaaatctcgctttgtttagatgacgaatctttacagcgactggcaagacagcggggtctagatccagccaagctgaaggccctgcgcacatgcgctctttctgttgaggtcagcaagagcgaggttaaaagacggctggcggaggctgagaaagacaccagcactgcgaccaccagactcgtggagctggccatcccgagacaagccagatccgtcagccgatccttcacggtgatgctccaagcgctcaataatgcaatcgatgatatgggtgctgatgctgagaaggtggttgctatggtgactggagagcgacagtag

>irgq3 837 bp

atggcaattcaatgcactcacagaatctgctcttatcttacaaattcgttgtttttcaggttcgttgtctccactgctttaaggtccatgaagataaaccaagatgaccttgaccagatctccaagctatcccagacgagagatttcacggacaatccctccaaactccaggcgatccttggagctctggatcacttccgcttggatgtcggtgtgctgggtgagacgggctgtggcagctccagcctgatcaatgcactcctgggcttgaagaacagtaatgaaaccgctgctttaactggtgtcactgaaaccactaaagaggctgtggagtatgccctccctgattcccacaacatccgtttttgggaccttcctggacttggaaaaataggtgatcttaacagcctgtcagccaatgcattcagttcttctgagggccaacaggtggcatctgtgcttgctttgtgtgatggatacatacacatactggtgtctcctttaagggttagattgagaaccatacaacttctgcagcaagcgtcctccatgggcaaagagtgctacctagtgatttccatggtggacttgattgaagataaggctgtcgaggaggtgagacaatggactgagaaagtcttgagtaaattggatattcagcagagcttgtttctggtatctgcgaactatccagaaaccctagatttagccaagctaaaggggatgttaaaagcagccatcccaagccataagaaagttgctctagccagatatgtttcaaagcaactggatgaagatgttttctggaaaagatcagattcttgcaaatttatgtaa

>irgf1 1197 bp

atggctacatttgaagactattgtgtaataacccaggaggacctggatgatattaaagattcaatatctacacaggatctcccatcagctgtaaacactataaaagagtatctcaaacagcaagatctcgttgaacttaacattggtgtgactggagagtctggttctggaaagtccacatttgtcaatgcattcaggggtttaggagatgaagatgagggctctgctgaaactggccctgtagaaaccactatggagcctgaagtttatattcacccgaaataccacaatgtgaaagtgtgggaccttcctggcattggaacaccaaattttaaagccgatgagtatctcgagttggttgagtttgagcgctatgattttttcattatcatcgcttcagatagatttagagaatgccacactcagctggccaaagagatcatgaggatggggaagaagttttactttgttcgctccaagattgatgcaagcattacagctgagaagaagaagaagaactttgaccagaaaaagacactggattccatccgcaaggactgtataaatggtctgagaaagatcggtatagaagatcccattgtattcctgatctcaggctgggagctcagcaagtatgatttaaatctgctgcaggatagaatggagaaagagcttccacagcataagagacgtgtactgatgttggctttgccaaatatcacactggagattaatgagaagaagaagaaagctcttgaggaaaacatcagaaaagttgcctttctgtctgcttgtgttgctcttttcccccttcctggtctttccatcagtgcagatatagccatcatagcagaagagctgagaaagtactacagtgcatttggtctggatgatccatccctgcagaaactctgtgaacgatcagggaaaaccgtagaggaactgaagagtctgatgaagtctcctctgcatcacgggatcaacccaagttcaatattaaccttgctgggcgctgcctctgttcttatatcagaagatgctgttgagttacttgtgagcttcatacccattattggctctgtggtggcaggaggactttcttatttgaccgtctcaggaatgctgaagaaagctctgaatgaaatagcagaagatgctaggaatgttttaatggcttcactagagactgaagtataa

>irgf2 1162 bp

gtggatgctttagaacatttgtatgaaataaaagtagaagataagctcaaggagatcaaagaaatcttatacacccaagatctgccaacagcatttggcacaatcagcaattattttaaggagacttctcttgtacttaacattggtgtgacaggagaatctggttctggaaagtccacatttgtcaatgcattcaggggtttaggagatgaagacgagggctctgctaaaaccagttcagtggtgaccactgcagagccggaagtttattttcatccaaaatatgaaaatgtgaaattgtgggatcttcctggcattggaacaccaaatttcaaagccgataagtatctcgagttggttgagtttgagcgctatgattttttcatcatcatcgcttcagatcgattcagagaatgccacactcagctggccaaagggatcatgaggatggggaagaagttttactttgttcgctccaaaattgatgcaagcattacagctgagaagaagaagaagaactttgaccagaaaaagacactggattccatccgagaagactgcgaaaatggtctgagaaagattgggatagaatatcctgttgtgttcctgatttctggatgggaccttggaaagtatgatttaaatctgctgcaggagatgatggagaaagagattctcaagtgtaagagaattctgctcaagtcagctttgctaaatgtcaaacaggaggttattgagcaaaggaaggatactcttaagagaaacattgaaagagtaacagagcagtctgtcgctatcactgatgttcacctcccgggtctttcaatttctgtgaatgtagacatcatagcagaagagttgacaaagtactacagtgagtttggtctggatgatcagagtctacagaaactctgtgaacgatctgggaaaaccatagaggaactgaagagtctgatgaagtctcccctgtgttatggaataaacacatctttaataataaacttgctggaggctgaagttcctaaaattgaaaatgagtattttttgagcttcatgccatttattggcactgaaataaaaaaaataaagtccagtgtggcagtctcatcaatgctgaagacagctctgaatgtcatagcagaagacatcagaaatgtgataa

>irgf3 1197 bp

atggatatattagaagactacgacataataactcagaatgacttggaggaaattaaggaatccatatctactgaggatctcccaaccgcagttagcaggatcagagaatatctcagaaaacaagatcttgtggagctaaatgttggtgtgacgggagagtctggttctggaaagtccacgtttgttaatgcattcaggggtttaggagatgaagacgagggctctgctgaaactggggttgtagaaaccaccatggagcctaaagcttataatcaccccaaaatacaacatgtgaaagtgtgggatcttcctggcattggaacaccaaacttcaaagctgatgagtaccttcagcaggttgagtttgaacgctttgattttttcatcatcattgcttcagatcggttcaaggaatgccacactcatctggccaaagagatcatgaggatggggaagaagttttactttgttcgttccaagattgacgcaagcattactgctgagaagaggaagaagaactttgacctaaaaaagacacttgatgtcattcgagaggactgtgtaaatggtctgagaaagattgggattgaagatcctgttgtgtttttaatctctaattttgagctcggcaagtacgatttaaatctgctggaggagaagatggaggaagagcttccacagcataagagacgtgtgctgttattggctttgccaaatatcacacaagagattaatgagaagaagaaggaagctctagggcaaaatattggaaaagtagccatactgtctgcttgtgtggctgctgttcccattcctggtctttcagttgctgtagatttagtaattgttaaaagggagatagaaatttactacagtacctttggtctagatgatccatccctgcagatgctgtgtgaaagatctgggaaaaccattgaggaattcaaaagtcttatgaagtcgccgctgagaggtgggataaacccagcttcattgttatccttggtcggtgcggtatctgtagttggagctgaaagtacagttgagtatattttgagcctcgtacccattcttggcactgtggtggcaggaggattgtcttatctgactgtctcaacaatgcttagaagagctctgaatgatatagcagaagatgccagaaatgtgctaaatgcttcactggagactgaagtctag

>irgf4 atgagtaatattagccaaaaagttgtgttattgtttgcagaacaagaggaactggtcgatcttagaaaagccatatctactcaagatctccctacagcaataaacaccattaaagaatgccttcgtaagcaagacctggttgagctgaacattggtgtgacaggagagtctggttctggaaagtccacatttgtcaatgcattcaggggtttaggaaatgaagaaaagggctctgcagaaaccggctttgaagaaaccactatggagcctaaagattatattcacccaaatttcaaaaacgtcagattgtgggatcttcctggtattggaacaccaaacttcaaagccaaggattaccttaaactggttaaatttgagcgttatgattttttcatcatcatttcatccgatcgatttaaggaacaccactctctgctggctgaggagattgtaagattgaggaaaaccttttactttgtccgttccaagattgaccagagcatcgattctgaaaagtacaaaaaaacctttgaccaggaaaagatgctggataacatccgagacaagtgtaaaagtgagctgagcaagattgtaaaggatcctgctgtgttcttgatctcctgcaatgagctcaacaaatatgatttccagctgctgcaggaacggatggagacggagcttccactgcataagagacgggtcctgatgttggctttgccgaatgtttcgctggatgtaattaagaaaaagaaggaagttctagagaaagacattgcaaaagttgccttcatatctgctactgtgtctgctgttcctattcctggtctttcagtcgctgtggatgtcatgatcataaaagaagagacggagaagtacttcaggggttttaatctggatgatgaatctctacagaggctctgtgatgtatcagggaagagcttagaggaaattaagagtttaatgaagtcgccactgaaagctgggattgggagttattcaatattagctttgctgagttctgcaaccttggttctgggtggaatgtcagttttagcagcagaaagtgcacttgagtattttctgagcaccatacctttgattggctctgtggctgcagcagcgatgtcttataaaacaatcacattaatgctgaagaaaactctgaatgatttagcaaaagatgctgaaactgttttcaaggcattgctggagactgaagtctag

**NUCLEOTIDE SEQUENCES FOR FUGU IRG GENES**

Sequences highlighted in red are treated as an intron in the main paper because of colinearity with the other p47 GTPases. But because both “exons” are in the same frame the real proteins might contain the “intron”.

>irgf5 1374 bp

atggtcaatgtgtgtgtctgctatataactgttggattatctgttgggatgatttcacgtttgtctgacttttacattgtaactgtcggctttgctttgtgtgtacaggtcatcatggctgattccctggacactacagaaatcaaagaagctctgcaaaataacaatcaagctttagctgttgataaaatcaaaaagcttctggagaagagagcaaacactcctcttaatatcggcattacaggagagtctggctctggaaaatcctcctttgtcaatgcctttagaggcgtggaccaccgggacaatcaagctgctcctacaggtgttgtagaaaccaccacagaagttagagcataccctcatccaagctatcccaatgttaccctctgggatcttcctggtattggtaccaccaggtttccagctgatcagtacctgaagcatgttgggtttgaaaggtttgatttcttcatcattatctcagctactcgctttagagaaaatgatgtgaagcttgcaaaagagatccagaagatggggaaaaagttctacttcgtgcgctccaaggttgacaacgatctgcagaatgcacaaaggagtcagagaaactttgatgcagagcagactctcgcacttattcgtgaaaactgcaaagaaggtacaaaagtcctgaatcctgtacatatgtgtgtcttcatgtggagtggcaatgatgatgatgtcatttcctgggacacaggtcttctgaaggaaggtgtgcaggctcctcaggtcttcctgctgtccaactttgagcttcggcgccatgacttccatcgcctccatgcgaccctggagagagaacttccagaacacaagcgggatgctctgctggtttctttggccaacatgagcctggagatcatcaagaagaagaaagaggccttcaagtcaaaaatccctcactatgcttttgtgtctgcagcttgtgcagcggtaccacttcctgggctttctgctgctgttgatgctgatctcatagctggtgttgtacagcagtacaaaactggttttgggctgggccggccttcactgcagcgactcgttgccattactggtgtaccactggtagatcttactatcatcagttcaccgttaactttagacaacataaatactgatttagtattaaatctaatgagccaatcatcagctatctccagtctaactgaaacaagggaaagctatagtttcattccattattcggaatcccggtagcaagaaaactttcttatgaaatcactgaaagagctctccacaatttcctagacatgcttactgaggatgctcaggatgtgtataacagggttattaatcacattaattcctaa

>irgf6 1407 bp

atggtcaatgtgtgtgtctgctatataactgttggattatctgttgggatgatttcacgtttgtctgacttttacattgtaactgtcggctttgctttgtgtgtacaggtcatcatggctgattccctggacactacagaaatcaaagaagctctgcaaaataacaatcaagctttagctgttgataaaatcaaaaagcttctggagagggcagcaaacactcctcttaatatcggcattacaggagagtctggctctggaaaatcctcctttgtcaatgcctttagaggcgtggaccaccaggacaatcaagctgctcctacaggtgttgtagaaaccaccacagaagttagagcataccctcatccaagctatcccaatgttaccctctgggatcttcctggtattggtaccaccaggtttccagctgatcagtacctgaagcatgttgggtttgaaaggtttgatttcttcatcattatctcagctactcgctttagagaaaatgatgtgaagcttgcaaaagagatccagaagatggggaaaaagttctacttcgtgcgctccaaggttgacaacgatctgcagaatgcacaaaggagtcagagaaactttgatgcagagcagactctcgcacttattcgtgaaaactgcaaagaaggtacaaaagtcctgaatcctgtacatatgtgtgtcttcatgtggagtggcaatgatgatgatgtcatttcctgggacacaggtcttctgaaggaaggtgtgcaggctcctcaggtcttcctgctgtccaactttgagcttcggcgccatgacttccatcgcctccatgcgaccctggagagagaacttccagaacacaagcgggatgctcttctgtttgccatgcccaacatgagcctggagatcatcgaaaagaagaaagaggcctttaagtcaaaaatccctcactatgcttttgtgtctgctgcgtgtgcagcggtaccagttcctgggctttctgttgctgttgatggtgctctgatagctggtgttgtacagcagtacaaaactggttttggtctggacggaccctcactgcagcggcttgctgacagcacaggtgtaccgctggaagatctgacctcggttgtccgctcaccactcagtttaaacaccattgataaggcatttatcttaaaacttctacttcagtcagcagctgttgctggcttaatgctagcagaggaaggcctgaaattcattccattatttggaaccttggtagcatcgactctctcttataaagtcactgaaaaagctctgctggatttcctccacatgctggctgaagatgctcagaacgtgtttaaaagggccctctgttgcatgaactcctcagtgtga

**NUCLEOTIDE SEQUENCES FOR TETRAODON IRG GENES**

>irgf7 1188 bp

atggctgattcttcagacattgtagaaataaaagaagctctgcgaaataacaatcaagctttagctgctgccaaaatcaaagagcttctggacaacccatcaaatgccactcttaatattggcatcaccggagagtcaggctctggaaaatcatcctttgtgaatgccttcagaggtgtcgaccacaaagatgagaaggaagctgctcctgtaggtgttgtagaaaccactgtggatgttaaagaatatccccatccagactatccaaatgttagcctctgggatcttcctggtattggtaccaccaagtttccagctgatgagtacctgaagcttgttggatttgagaagtttgatttcttcatcataatctctgatacttgctttagaaaaaatgatgtaaagcttgcaaaagagatccagaagatgggaaaaaagttctacttcgtacgctccaaagttgacaacgacctgctgaatgcacaaaggagtcagagagactttgatccagagaagactctttcacgtattcgtgataactgcaaaaaagggcttctcaacgcaggtgtgcaggctcaggtttttgtgctgtccaactttgagcttcagcgctacgacttccatctcctacatgagaccctggagagagaacttccagagcataaaaggaatgtcctgttggttgccatgcctaacatcagcctggagatcattgagaagaagaaagaggccttcaaatcaaaaatacctctctgggcttttgtgtcagcagcaggtgcagtggtaccagttcctggactctctgttgctgttgatcttagtctgatagttggtcttgtacaacagtacaaaactagttttggtctggacacaccttcactgcagcgacttgctgacaccacaggtgttcaactgactgatctaacctcggtcatccgatcaccactgagtttagacaatataaatgctcaacttattacgcaaactctaaatcaaacagcaagtgttgctggtttaatggcagcagaggaaggtctgagattttttccaatatttggaacaatgatagcagggagtctttcttgtgcagttatttataaagctctgtcagatttccttgagatgctaactgacgatgctcagtatgtgtttgaaaaggctctccgctgcatgaactcctcagtgtga

>irgf8 1206 bp

atggctgattcttcagactttgcagaaataaaagaagctctgcaaaataacaatcaagctttagctgctgccaaaatcaaagagcttctggacaacacatcaaataccactcttaatattggcatcaccggagaggcaggctctggaaaatcatcctttgtgaatgccttcagaggtgtcgacgacagagatgagaaagctgctcctgtaggtgttgtagaaaccactgcagaagttaaagaatatccccatccaaactatccaaatgttagcctctgggatcttcctggtattggtaccaccaagtttccagctgatgagtacctgaagcttgttggatttgagaagtttgatttcttcatcataatctcagagactcgctttagagaaaatgatgtaaagcttgcaaaagagatccagaagatgggaaaaaagttctacttcgtacgctccaaggttgacaacgacctgcagagtgaacaaagatatcagagagactttgatccagagaagactctttcacttattcgtgaaaactgcaaacgagggcttctcaacgcaggtttgcaggctcaggtttttctgctgtccagctttgagcttcagcgctacgacttccatctcctatatgagaccctggagagagaatttccagagcatcagagggatgttctgttggttgccatgtctaacatcagcctggagatcaatgggaagaagaaagaggccttcaaatcaaaaataccttactgggctttagtgtcttcagtaggtgcattggtaccagttcctggactctctgttgctgttgatcttagtctgatagctggtcttgtacaacagtacaaaactggttttggtctgggcagaccttcactgcagcgacttgctgacaccacaggtgttcaactgactgatctaacctcggtcatccgatcaccactgggtttaaacataatagatgctgaacttattgtgaaagctctaagtgaattagcaagtgttgctggtttaatggcagcagaggaaggtctgagatttattccaatatttggaacaatgatagcagggactctttcttatgcagctacttataacgctctgtcagatttcctcaagatgctaactgaagatgctcagaatgtgtttgaaaaggctctccgctgcatgaactcctcagtgtga
