# Supplementary material for: The interferon-inducible p47 (IRG) GTPases in vertebrates: loss of the cell autonomous resistance mechanism in the human lineage
Source: Genome Biol. 2005 Oct 31;6(11):R92. doi: 10.1186/gb-2005-6-11-r92 (PMC1297648; doi:10.1186/gb-2005-6-11-r92)
Supplement: Additional data file 10 — Protein sequences of all IRG family members [file gb-2005-6-11-r92-S10.doc]

**PROTEIN SEQUENCES FOR HUMAN IRG GTPASES**

>IRGC

MATSKLPVVPGEEENTILMAKERLEALRTAFESGDLPQAASHLQELLASTESIRLEVGVTGESGAGKSSLINALRGLEAEDPGAALTGVMETTMQPSPYPHPQFPDVTLWDLPGAGSPGCPADKYLKQVDFSRYDFFLLVSPRRCGAVETRLAAEILCQGKKFYFVRTKVDEDLAATRTQRPSGFREAAVLQEIRDHCAERLREAGVADPRIFLVSNLSPARYDFPTLVSTWEHDLPSHRRHAGLLSLPDISLEALQKKKAMLQEQVLKTALVLGVIQALPVPGLAAAYDDALLIHSLRGYHRSFGLDDDSLAKLAEQVGKQAGDLRSVIRSPLANEVSPETVLRLYSQSSDGAMRVARAFERGIPVFGTLVAGGISFGAVYTMLQGCLNEMAEDAQRVRIKALEDDEPQPEVSLEVASDNGVEKGGSGEGGGEEAPLSTCRKLGLLLKYILDSWKKHDSEEK*

>IRGM(a)

MEAMNVEKASADGNLPEVISNIKETLKIVSRTPVNITMAGDSGNGMSTFISALRNTGHEGKASPPTELVKATQRCASYFSSHFSNVVLWDLPGTGSATTTLENYLMEMQFNRYDFIMVASAQFSMNHVMLAKTAEDMGKKFYIVWTKLDMDLSTGALPEVQLLQIRENVLENLQKERVCEY*

>IRGM(b)

MEAMNVEKASADGNLPEVISNIKETLKIVSRTPVNITMAGDSGNGMSTFISALRNTGHEGKASPPTELVKATQRCASYFSSHFSNVVLWDLPGTGSATTTLENYLMEMQFNRYDFIMVASAQFSMNHVMLAKTAEDMGKKFYIVWTKLDMDLSTGALPEVQLLQIRENVLENLQKERLACHEKYLKSTPENSTRPRNIPSRRKLYVNLLRIFNS*

>IRGM(c)

MEAMNVEKASADGNLPEVISNIKETLKIVSRTPVNITMAGDSGNGMSTFISALRNTGHEGKASPPTELVKATQRCASYFSSHFSNVVLWDLPGTGSATTTLENYLMEMQFNRYDFIMVASAQFSMNHVMLAKTAEDMGKKFYIVWTKLDMDLSTGALPEVQLLQIRENVLENLQKERSSRNQQVYPNHSHVPLAC*

>IRGM(d)

MEAMNVEKASADGNLPEVISNIKETLKIVSRTPVNITMAGDSGNGMSTFISALRNTGHEGKASPPTELVKATQRCASYFSSHFSNVVLWDLPGTGSATTTLENYLMEMQFNRYDFIMVASAQFSMNHVMLAKTAEDMGKKFYIVWTKLDMDLSTGALPEVQLLQIRENVLENLQKERLACHEKYLKSTPENSTRPRNINLCS*

>IRGM(e)

MEAMNVEKASADGNLPEVISNIKETLKIVSRTPVNITMAGDSGNGMSTFISALRNTGHEGKASPPTELVKATQRCASYFSSHFSNVVLWDLPGTGSATTTLENYLMEMQFNRYDFIMVASAQFSMNHVMLAKTAEDMGKKFYIVWTKLDMDLSTGALPEVQLLQIRENVLENLQKERSSRNQQVYPNHSHVPLAC*

**>**IRGQ

RLLPPAQDGFEVLGAAELEAVREAFETGGLEAALSWVRSGLERLGSARLDLAVAGKADVGLVVDMLLGLDPGDPGAAPASVPTAPTPFPAPERPNVVLWTVPLGHTGTATTAAAASHPTHYDALILVTPGAPTEKDWAQVQALLLPDAPLVCVRTDGEGEDPECLGEGKMENPKGESLKNAGGGGLENALSKGREKCSAGSQKAGSGEGPGKAGSEGLQQVVGMKKSGGGDSERAAALSPEDETWEVLEEAPPPVFPLRPGGLPGLCEWLRRALPPAQAGALLLALPPASPSAARTKAAALRAGAWRPALLASLAAAAAPLPGLGWACDVALLRGQLAEWRRGLGLEPTALARRERALGLASGELAARAHFPGPVTRAEVEARLGAWAGEGTAGGAALGALSFLWPAGGAAATGGLGYRAAHGVLLQALDEMRADAEAVLAPPEPAQ*

**PROTEIN SEQUENCES FOR DOG IRG GTPASES**

>IRGC

MATSKLRAVPGEEETTILMAKEELEALRSAFESGDIPQAASRLRELLASSQSIRLEVGVTGESGAGKSSLINALRGVGAEDPGAALTGVVETTMQPSPYPHPQFPDVTLWDLPGAGSPGCPADKYLKQVDFGRYDFFLLVSPRRCGAVETRLASEILRQGKKFYFVRTKVDEDLAATRTQRPSGFSEAAVLQEIRDHCAERLRVAGMTDPRIFLVSNLSPARYDFPLLMSTWEHDLPAHRRHAGLLSLPDISLEALQKKKDMLQEQVLKTALVSGVIQALPVPGLAAAYDDALLIRSLRGYHRSFGLDDDSLAKLAEQVGKQAGDLRSVIRSPLANEVSPETVLRLYSQSSDGAMRVARAFEKGIPVFGTLVAGGISFGTVYTMLQGCLNEMAEDAQRVRIKALEEDEPQSEVSLEAAGDNGVEKRGSGEGGCEEAPLSARRKLGLLLKYILDSWKKRDLSEEK*

>IRGB11

MGQSPPSTPSNRNGGDLASSFDKFFKEFKLDSKIISQETISTIQSHLEKGDLQSAFSAINDALRDIDNAPLNIAVTGESGTGKSSFINALRGMGHDEEGAAPTGPVETTFLRKAYKHPKFPNVTFWDLPGIGTTSFQPQDYLEKMVFREYDFFIIICATRFKINDVQLATAIKKMKKNFYFVRSKVDSDLYNLKRIKPREFNKDEILQKIRNDCVKHLMEANMSDAQVFLVSSFELSDYDFQSLETTLLRELPSHKRHIFMQYLPIVTEATIDRKRDCLRQKVWLEAIKAGASASIPLVGYISDNDVETLKDTLTLYRSYFGLDDISLKTIAKDLNVSVEKLKANLMFPHLLSVEKYDEPLGEKLLKYVEKFCSVSGGPIAAGIYFRKIYYLKNYFLDTVVSDAKVLLKKEEIFKDPVDSEQTYLHTNVGNENGKSDTSSS*

>IRGB12

MGQSSSTPSHKTGGDLASSFGKFFKDFKLESKILSQEAITSIEKSLKEGNLQKAVSDINKALKDIDNAPLSIAVTGESGTGKSSFINALRGVGHDEEGAAPIGAVETTFDRTEYKHRKFPNVTLWDLPGVGTTTFHPQEYLEKMKFREYDFFIIISSTRFTINDAQLATAIRKMKKNFYFVRSKVDSDLYNLKRTKPSDFNKDEILLKIRNDCITQLQNVKVCDPQVFLVSNLDLSSYDFQSLETTLLKELPAHKRHIFMQYLPNITESAIDRKRDSLRQKVWLEAVKAGASATIPFMGLINDNEVEKLEETLHLYRSYFGLDDASLETIAKDLNVSVEKLKANLTSPHLLSVEKEDESLGEKLLRYVEKFCSVSGGLIATGVYFRKIFYLQNYFLEAVVSDAKVLLNKEEIFKETVGSGQAYLLQDVGIENRKSDATSS*

>IRGD

MDKFMCDFLVGKNFQQLAINFIPHYTTLVNKAGGIIASENLDRIQAALKEAKLKDVADIIEESLVAAENAPLDVAVIGESGTGKSSFINALRGLSYEEEGSASVGVVETTMKKTPYQHPKYPKVTFWDLPGTGTPNFHPHEYLEMVEFATYDFFIIISSSRFSLNDALLAQNIKEIGKKFYFVRTKVDNDLYNEEKSKPMSFKRERVLQQIRDNCLANLSNIGVPEPCIFLVSNFDLDDFDFPRLEETLLKELPVHKRHIFALLLPNLSYTSIEMKRAFFKEKIWLDALKSSALSFIPFMACFNGFDFPQQEKCLNLYQSHFGLDEKSVKGIAEKLDMSVEEIKSFTKSLDFWLLVKDDSIAEKAMKCVECYCSVNGGLPSTIFQFFKIYFLHLKFINTVADDAKILLHKTLEILSHRR*

>IRGM4

MAQPTQSLHTPSPTSFTSTVPYHKGGSILSESGAMNIEKALGEGKLLDMVSVVRETLETASSVPVSIAVTGDSGNGMSTFINALRKIGHNEEDSAPTGVVRTTQIPTCYSFSDIPNVELWDLPGTGAATQNLETYLEEMQFSKYDLFIIIASEQFSMNLVKLVKSIQGQGKRFYIVWTKLDRDLSTCVLSEEQLLRNIRENIRETLHKEGVCEPIIFLVSSFNPFLHDFPELRKSLHRDISNIGYRGHLENLTHTCEKVINGKVTTLQGQIGSKSFQDILGIQNANDLGEFLNAYHRLFGVDDDSLQEVAQSMGKPKEEYKAIMKSQDLHTALAWDWALSWMNCNAASYLYSVLSYIPILGTTGIHYLKWWSQGHLLEIVAEDTKTILKKILEDAII*

>IRGM5

MTQPNHSLHIPLSTSFTSIVPYNMGWTVLPKATATNIEKALGDGKLLEVVSMIRETLETVSSAPVSIAVTGDSGNGMSSFINALREIGHDEKDSAPTGVVRTTQVPTCYSSSHFPYMELWDLPGTGTGTQSLENYLEKIHFSQYDLFIIIASEQFSMNLVKLVKAIQRQGKRFYIVWTKLDRDLSTRVLPEEQVLQNIWENIQETLQKVGVCEPIIFLVSSFEPLLHDFPELRDALNRDISDIRYCGPLENLSDTCEKIINDKVTSFQEQIGSKTFQDILGIQDEDDLGQCLIAYHLFFGVDDKSLQQMAQSMGKPMEEYRAIMKSQDVHTVLTGDWALSCMNCKTASYLYSILSYIPFLGDTVINYLRVWKHRHFLEIVAKDTRSIVKKILTDSII*

>IRGM6

LHCFFPLLQVTPLLSDVTQPTHSLHTPLLTSSNYDMPYNMGWSSLSKETAINIEKALGGRKLLEVVPMVRETLERASSVPLRIAVTGDSGNGMSSFINALRGIGHDEEDSAPTGVVKTTQIPTCYSYPHFPNVELWDLPGTGAGTQSLENYLEEMKFSWYDLFIIIASEQFSMNLVKLAKAIQVLGKRFYIVWTKLDRDLSTSALLKERLLQNIQENIQENLQKERVFEPIIFLVSSFEPLLHDFPELRNTLNRDISDIRYCGPLKNLSHTYEKVISDKVTMFRGKIASKSFDTLGIWNADDLGECLIAYHLFFGVDDESLQQIAQSMGKPMEEYRAIMKSRDLHTIIRGDWAVSCMNCNTSSCLYTILRYIPLLGDFIINFLRKWKHRRLLEIVAEDTRTILKKILKDSII*

**PROTEIN SEQUENCES OF MOUSE IRG FAMILY MEMBERS**

>Irga1

MGQLFSLLKNKCQFLVSSVAEYFKKFKKIVIIILQEVTTSIELDMKKENFQEANSAICDALKEIDSSLVNVAVTGETGSGKSSFINTLRGIGHEEEGAAKTGVVEATMERHPYKHPNMPNVVFWDLPGIGSTKFPPKTYLEKMKFYEYDFFIIISATCFKKNDIDLAKAISMMKKEFYFVRTKVDTDLRNEEDFKPQTFDKEKVLQDIRLNCVNTFKENGIAEPPIFLISNENVCHYDFPVLMDKLISDLPDYKRHNFMLSLPNITDSVIETKRQSLKQRHWLQGFAGVLLSYLH*

>Irga2

MGQLFSSRRSEDQDLSSSFIEYLKECEKGINIIPHEIITSIEINMKKGNIQEVNSTVRDMLREIDNTPLNVALTGETGSGKSSFINTLRGIGHEEGGAAHTGVTDKTKERHPYEHPKMPNVVFWDLPGTGSEDFQPKTYLEKMKFYEYDFFIIISATRFKKNDIDLAKAIGIMKKEFYFVRTQVDSDLRNEEDFKPQTFDREKVLQDIRLNCVNTFRENGIAEPPIFLISNKNVCHYDFPVLMDKLISDLPVFKRQNFMFSLPNITDSVIEKKRNFLRWKTWLEGFADGLLSFFLESDLETLEKSMKFYRTVFGVDDASLQRLARAWEIDQVDQVRAMIKSPAVFTPTDEETIQERLSRYNQEFCLANGYLLPKNHCREILYLKLYFLDMVTEDAKTLLKEICLRN*

>Irga3

MGQLFSHIPKDEDKGNLESSFTEYFRNYKQETKIISEETTRSIELCLKRGDFQRANSVISDALKNIDNTPINIAVTGESGAGKSSLINALREVKAEEESAAEVGVTETTMKVSSYKHPKVKNLTLWDLPGIGTMKFQPKDYLEKVEFKKYDFFIIVSSSRFTKLELDLAKATRIMKKNYYFVRSKVDCDLDNEKKSKPRNFNRENTLNQVRNSYLDTFRESKIDEPQVFLISNHDLSDYDFPVLMDTLLKDLPAEKRQNFLLSLPNITEAAIQKKYNSTKQIIWLQATKDGLLATVPVVGILKDLDKERLKKRLDYYRDLFGVDDESLMFMAKDAQVPVELLIKNLKSPNLLKCKEETLEELLLNCVEKFASANGGLLAAGLYFRKTYYLQFHFLDTVAEDAKVLLKAAQTHFAHSF*

>Irga4

MGQLLSDTSKTEDNEDLVSSFNEYFKNIKTEKIISQETIDLIKLYLNKGNIHGANSLISDALRNIDNAPINIAVTGESGAGKSSLINALIGIGPEEEGAAEVGVIETTMKRTSYKHPKIETLTLWDLPGIGTQKFPPKTYLEEVKFKEYDFFIIVSATRFTKLELDLAKAITNMKKNYYFVRTKVDIDVENERKSKPRTFEREKALKQIQSYSVKIFNDNNMAVPPIFLISNYDLSDYDFPFLVDTLIKELHVQKRHNFMLSLPNFTDQAIDRKYKATQQFIWLEAFKIGVVAIFPVLGNLRNKDMKKIKNTLNYYQKIFGVDDESLELVAKDFQVPVEQVKKTMKTPHLLKKYREETFRNDFKKLVSTFGRLLAVGLYFPAIYYLQLHILDTVTEDAKVLLRWKYSKPRSNSTYP*

>Irga5(Edited)

MGQLFSGTSKSEALCSSFTEYFQKFKVENKIISQEISTLIELYLTLGDVQQANNAITYALRXLARTPQNVALIGESGRGKYSFINVFRGLDMKRKMATVGVVETTMNRTPYRNPNIPNVIIWDLPGIGTTNFPPKHYLKKMQFYVMYDFFIIVSATCFRKNDIDLSKAVVMIKKKDFLLRTKEDIDIENEN*

>Irga6

MGQLFSSPKSDENNDLPSSFTGYFKKFNTGRKIISQEILNLIELRMRKGNIQLTNSAISDALKEIDSSVLNVAVTGETGSGKSSFINTLRGIGNEEEGAAKTGVVEVTMERHPYKHPNIPNVVFWDLPGIGSTNFPPNTYLEKMKFYEYDFFIIISATRFKKNDIDIAKAISMMKKEFYFVRTKVDSDITNEADGKPQTFDKEKVLQDIRLNCVNTFRENGIAEPPIFLLSNKNVCHYDFPVLMDKLISDLPIYKRHNFMVSLPNITDSVIEKKRQFLKQRIWLEGFAADLVNIIPSLTFLLDSDLETLKKSMKFYRTVFGVDETSLQRLARDWEIEVDQVEAMIKSPAVFKPTDEETIQERLSRYIQEFCLANGYLLPKNSFLKEIFYLKYYFLDMVTEDAKTLLKEICLRN*

>Irga7

MDQLLSDTSKNEDNDDLVSSFNAYFKNIKTENKIISQETIDLIELHLNKGNIHGANSLIREALKNIDNAPINIAVTGESGVGKSSFINALIGTGPEEEGAAEVGVIETTMKRNFYKHPKIETLTLWDLPGIGTQKFPPKTYLEEVKFKEYDFFIIVSSTRFTKHELDLAKAIGIMKKNYYFVRTKVDIDLENERKSKPRTFDREKTLKQIQSYAMNTFSDNNMAIPPIFMVSNYDLSKYDFPVMMDTLIKDLHAEKRHNFMLSLPGITEAAIDRKHKATQQIVWLEAFNVGLLANFPVTGILGDNDVKKLEKSLNYYRKIFGVDDESLELVAKDFQVPVEQVKEIMKSPHLLKTNGKETLGEKLLKYLEKFETATGGLLAVGLYFRKTYYLQLHFLDTVTEDAKVLLRWKYSKPRSNSTYP*

>Irga8

MGQLFSNMPKDEDKGNLESSFTEYFRNYKQETKIISEETTRSIELCLKKGDIQRANSIISDALKNIDNAPINIAVTGESGAGKSSLINALREIKAEEESAAEVGVTETTMKVYSYKHPKVKNLTLWDLPGIGTKKFPPKTYLETVEFKKYDFFIIVSAIRFTNHEIELAKAIRIMKKNYYFVRSKVDFDLYNEEKSKPRNFNRENTLNQVRNYYLDTFRESKIDEPQVFLISNHDLSDYDFPVLMDTLLKDLPAEKRHNFLLSLPNITEAAIQKKYNSPKQYIWLQAMEDGLLATVPAVGILKDLDKERLKRSLDYYRDLFGVDDESLMFMAKDAQVPFELLKIKLKSPYLLELEEETLGGLILNCVEKFASANGGLLATGLYFRKTYYLQFHFLDTVAEDAKVLLKEAY*

>Irgb1

QHPPLNTATCQTSTGRTSQITAQLLEFNFKNFFKNFKKESKILSEETITLIESHLENKNLKEALTVISHALRNIDKAPLNIAVTGETGTGKSSFINALRGISSEEKDAAPTGVIETTMKRTPYPHPKLPNVTIWDLPGIGSTNFPPQNYLTEMKFGEYDFFIIISATRFKEIDAHLAKAIAKMNIKFYFVRTKIDQDISNEQRSKPKSFNRDSVLKKIKDECLGLLQKVLSSQPPIFLVSNFDVSDFDFPKLETTLLKELPAHKRHLFMMSLHSVTETTIARKRDFLRQKIWLEALKAGLWATIPLGGLVRDKMQKLEETLTLYRSYFGLDEASLENIAKDFNVSVNEIKAHLRSLQLLTKNNDMSFKEKLLKYIEYISCVTGGPLASGLYFSKTYYWQSLFIDTVASDAKSLLNKEEFLSEKPGSCLSDLPEYWETGMEL*

>Irgb2

MGQTSSSTSPPKEDPPLTFQVKTKVLSQELIASIESSLEDGNLQETVSAISSALGDIEKVPLNIAVMGETGAGKSSLINALQGVGDDEEGAAASTGVVHTTTERTPYTYTKFPSVTLWDLPSIGSTAFQPHDYLKKIEFEEYDFFIIVSAIRIKQSDIELAKAIVQMNRGLYFVRTKTDSDLENEKLCNPMRFNRENILKSIRICLSSNLKER

FQQEPPVFLVSNFDVSDFDFPKLESTLLSQLPAYKHQIFMSTLQVVINAIVDRKRDMLKQKIWKESIMPRAWATIPSRGLTQKDMEMLQQTLNDYRSSFGLNEASLENIAEDLNVTLEELKANIKSPHLFSDEPDTSLTEKLLKYIGNPYFSKVFHLQNYFIDTVASDAKIILSKEELFTEQVSSFNSKASPYREESVGKVFPVSPGSTFLFHFFEMFQSDSDKLCHVHVLLLLTSWGLSGETVT*

>Irgb3

MAQLLVFSFENFFKNFKKESKILSEETITLIESHLEDKNLQGALSEISHALSNIDKAPLNIAVTGETGTGKSSFINALRGVRDEEEGAAPTGVVETTMKRTPYPHPKLPNVTIWDLPGIGSTTFPPQNYLTEMKFGEYDFFIIISATRFKEIDAHLAKTIEKMNTKFYFVRTKIDQDVSNEQRSKPRSFNRDSVLKKIRDDCSGHLQKALSSQPPVFLVSNFDVSDFDFPKLETTLLRELPSHKRHLFMMSLHSVTETAIARKRDFLRQKIWLEALKAGLWATIPLGGLVRNKMQKLEETLTLYRSYFGLDEASLENIAKDFNVSVNEIKAHLRFLQLFTKNNDMSFKEKLLKYIEYISCVTGGPLASGLYFRKTYYWQSLFIDTVASDAKSLLNKEEFLSEKPGSCLSDLPEYWETGMEL*

>Irgb4

QHPPLHTATCQPSSSRPSRLTLQLLVFSFENFFKNFKKESKILSEETITLIESHLEDKNLQGALTEISHALSNIDKAPLNIAVTGETGTGKSSFINALRGVRDEEEGAAPTGVVETTMKRTPYPHPKLPNVTIWDLPGIGSTTFPPQNYLTEMKFGEYDFFIIISATRFKEIDAHLAKTIEKMNTKFYFVRTKIDQDVSNEQRSKPRSFNRDSVLKKIRDDCSGHLQKALSSQPPVFLVSNFDVSDFDFPKLETTLLRELPSHKRHLFMMSLHSVTETAIARKRDFLRQKIWLEALKAGLWATIPLGGLVRNKMQKLEETLTLYRSYFGLDEASLENIAKDFNVSVNEIKAHLRSLQLFTKNNDMSFKEKLLKYIEYISCVTGGPLASGLYFRKTYYWQSLFIDTVASDAKSLLNKEEFLSEKPGSCLSDLPEYWETGMEL*

>Irgb5

MGQTSSSTPPPKEDPDLTSSFGTNLQNFKMKT

KILSQELIAFIESSLEDGNLQETVSAISSALGGIEKAPLNIAVMGETGAGKSSLINALQGVGDDEEGAAASTGVVHTTTERTPYTYTKFPSVTLWDLPGIGSTAFQPHDYLKKIEFEEYDFFIIVSSGRFKHNDAELAKAIVQMNRSFYFVRTHTDLDLMVVKRSNPRRFNRENTLKQIRHTISSMLKEVTHQEPPVFLVSNFDVSDFDFPKLESTLLSQLPAYKHHMFMLTLPIVTDSTIDRKRDMLKQKVWKESTMPRAWATIPSLGLTQKDMEMLQQTLNDYRSSFGLDEASLENIAEDLNVTLEELKANIKSPHLLSDEPDTSLTEKLLKYIGNPYFSKVFHLQNYFIDTVASDVKIILSKEELFTEQVSSFNSKASLYREESVGKVFPVGPGSTFLFHFIEMFQSDSDELCHVHVLLLLTSGGLSSETVT*

>Irgb6

MAWASSFDAFFKNFKRESKIISEYDITLIMTYIEENKLQKAVSVIEKVLRDIESAPLHIAVTGETGAGKSTFINTLRGVGHEEKGAAPTGAIETTMKRTPYPHPKLPNVTIWDLPGIGTTNFTPQNYLTEMKFGEYDFFIIISATRFKENDAQLAKAIAQMGMNFYFVRTKIDSDLDNEQKFKPKSFNKEEVLKNIKDYCSNHLQESLDSEPPVFLVSNVDISKYDFPKLETKLLQDLPAHKRHVFSLSLQSLTEATINYKRDSLKQKVFLEAMKAGALATIPLGGMISDILENLDETFNLYRSYFGLDDASLENIAQDLNMSVDDFKVHLRFPHLFAEHNDESLEDKLFKYIKHISSVTGGPVAAVTYYRMAYYLQNLFLDTAANDAIALLNSKALFEKKVGPYISEPPEYWEA*

>Irgb7

PFWFVPPLGTIDICQDWVKLPLLHPLQRRILLLTFQMKTKILSQELITFIELYLEDGNLXETVSAISSALGDIEKVPLNIAVMGETGAGKSSLINALQGTGADEDGVTAPVGVVYTTIEKKSYPYAKFPSAILWELPAIGFHHFQPHDYLKKIKFEEYDFIIVSAGRIKHSDVELAKAIVQMNRGLYFNRTKTDIDLKNEKLYNPMRFNRENTLKSLQICISSNLKECFHQEPPVFLVSNFDVSDFDFPKLESTLLSQLPAYKHQIFMRTLQVVINAIVDWKRDMLKQKVWKESTTPRAWATIPSLGLTQKDMEMLQQTLNDYRSSFGLDEASLKNIAEDLNVTLEELKANIKSPHLLSDEPDTSLTEKLLKYIGNPYFSKVFHLQNYFIDTVASDVKIILSKEELFTEQVSSFNSKASPYREESVGEVFPVGPGSTFLFHFFEMFQSDSDKLCHVHVLLLLTSWGLSGETVT*

>Irgb8

MAQLLVISFENFFKNFKKESKILSEETITLIESHLEDKNLQGALSEISHALSNIDKAPLNIAVTGETGTGKSSFINALRGVRGEEEGAAPTGVVETTMKRTPYPHPKLPNVTIWDLPGIGSTNFQPQNYLTEMKFGEYDFFIIISATRFKEIDAHLAKAIAKMNTKFYFVRTKIDQDVSNEQRSKPKSFNRDSVLKKIRDDCSGHLQKVLSSQPPVFLVSNFDVSDFDFPKLENTLLRELPAHKRHLFMMSLHSVTETAIDRKRDFLRQRIWLEALKAGVWTTIPLGGLVRDKMQKLEETLTLYRSYFGLDEASLENIAKDFNVSVNEIKAHLRSLQLLTKNNDMSFKEKLLKYIEYISCVTGGPLASGLYFRKTYYWQSLFIDTVASDAKSLLNKEEFLSEKPGSCLSDLPEYWETGMEL*

>Irgb9

MGQTSSSTLPPKDDPDFIASFGTNLQNFKMKTKILSQELIAFIESSLEDGNLRETVSAISSALGGIEKAPLNIAVMGETGAGKSSLINALQGVGDDEEGAAASTGVVHTTTERTPYTYTKFPSVTLWDLPGIGSTAFQPHDYLKKIEFEEYDFFIIVSSGRFKHNDAELAKAIVQMNRSFYFVRTHTDLDLMVVKLSDPRKFNKENILEQIRNSISNILKEVTHQEPPVFLVSNFDVSDFDFPNLESTLLSQLPAYKHHMFMLTLPIVTDSTIDRKRDMLKQKIWKESIMPRAWATIPSRGLTQKDMEMLQQTLNDYRSSFGLDEASLENIAEDLNVTLEELKANIKSPHLLSDEPDTSLTEKLLKYIGNPYFSKVFHLQNYFIDTVASDVKIILSKEELFTEQVSSFNSKASPYWEESVGKVFPVGPGSTFLFHFFEMFQSDSDKLCHVHVLLLLTSWGLSGETVT*

>Irgb10

MGQSSSKPDAKAHNMASSLTEFFKNFKMESKIISKETIDSIQSCIQEGDIQKVISIINAALTDIEKAPLNIAVTGETGAGKSTFINALRGIGHEESESAESGAVETTKDRKKYTHPKFPNVTIWDLPGVGTTNFKPEEYLKKMKFQEYDFFLIISSARFRDNEAQLAEAIKKMKKKFYFVRTKIDSDLWNEKKAKPSSYNREKILEVIRSDCVKNLQNANAASTRGFLSLKL*

>Irgc

MATSRLPAVPEETTILMAKEELEALRTAFESGDIPQAASRLRELLANSETTRLEVGVTGESGAGKSSLINALRGLGAEDPGAALTGVVETTMQPSPYPHPQFPDVTLWDLPGAGSPGCSADKYLKQVDFGRYDFFLLVSPRRCGAVESRLASEILRQGKKFYFVRTKVDEDLAATRSQRPSGFSEAAVLQEIRDHCTERLRVAGVNDPRIFLVSNLSPTRYDFPMLVTTWEHDLPAHRRHAGLLSLPDISLEALQKKKDMLQEQVLKTALVSGVIQALPVPGLAAAYDDALLIRSLRGYHRSFGLDDDSLAKLAEQVGKQAGDLRSVIRSPLANEVSPETVLRLYSQSSDGAMRVARAFERGIPVFGTLVAGGISFGTVYTMLQGCLNEMAEDAQRVRIKALEEDEPQGGEVSLEAAGDNLVEKRSTGEGTSEEAPLSTRRKLGLLLKYILDSWKRRDLSEDK*

>Irgd

MDQFISAFLKGASENSFQQLAKEFLPQYSALISKAGGMLSPETLTGIHKALQEGNLSDVMIQIQKAISAAENAILEVAVIGQSGTGKSSFINALRGLGHEADESADVGTVETTMCKTPYQHPKYPKVIFWDLPGTGTPNFHADAYLDQVGFANYDFFIIISSSRFSLNDALLAQKIKDAGKKFYFVRTKVDSDLYNEQKAKPIAFKKEKVLQQIRDYCVTNLIKTGVTEPCIFLISNLDLGAFDFPKLEETLLKELPGHKRHMFALLLPNISDASIELKKHFLREKIWLEALKSAAVSFIPFMTFFKGFDLPEQEQCLKDYRSYFGLDDQSIKEIAEKLGAPLADIKGELKCLDFWSLVKDNSIIAQATSAAEAFCAVKGGPESSAFQALKVYYRRTQFLNIVVDDAKHLLRKIETVNVA*

>Irgm1

MKPSHSSCEAAPLLPNMAETHYAPLSSAFPFVTSYQTGSSRLPEVSRSTERALREGKLLELVYGIKETVATLSQIPVSIFVTGDSGNGMSSFINALRVIGHDEDASAPTGVVRTTKTRTEYSSSHFPNVVLWDLPGLGATAQTVEDYVEEMKFSTCDLFIIIASEQFSSNHVKLSKIIQSMGKRFYIVWTKLDRDLSTSVLSEVRLLQNIQENIRENLQKEKVKYPPVFLVSSLDPLLYDFPKLRDTLHKDLSNIRCCEPLKTLYGTYEKIVGDKVAVWKQRIANESLKNSLGVRDDDNMGECLKVYRLIFGVDDESVQQVAQSMGTVVMEYKDNMKSQNFYTLRREDWKLRLMTCAIVNAFFRLLRFLPCVCCCLRRLRHKRMLFLVAQDTKNILEKILRDSIFPPQI*

>Irgm2

MPTSRVAPLLDNMEEAVESPEVKEFEYFSDAVFIPKDGNTLSVGVIKRIETAVKEGEVVKVVSIVKEIIQNVSRNKIKIAVTGDSGNGMSSFINALRLIGHEEKDSAPTGVVRTTQKPTCYFSSHFPYVELWDLPGLGATAQSVESYLEEMQISIYDLIIIVASEQFSLNHVKLAITMQRMRKRFYVVWTKLDRDLSTSTFPEPQLLQSIQRNIRDSLQKEKVKEHPMFLVSVFKPESHDFPKLRETLQKDLPVIKYHGLVETLYQVCEKTVNERVESIKKSIDEDNLHTEFGISDPGNAIEIRKAFQKTFGLDDISLHLVALEMKNKHFNTSMESQETQRYQQDDWVLARLYRTGTRVGSIGFDYMKCCFTSHHSRCKQQKDILDETAAKAKEVLLKILRLSIPHP*

>Irgm3

MDLVTKLPQNIWKTFTLFINMANYLKRLISPWSKSMTAGESLYSSQNSSSPEVIEDIGKAVTEGNLQKVIGIVKDEIQSKSRYRVKIAVTGDSGNGMSSFINALRFIGHEEEDSAPTGVVRTTKKPACYSSDSHFPYVELWDLPGLGATAQSVESYLEEMQISTFDLIIIVASEQFSSNHVKLAITMQRMRKRFYVVWTKLDRDLSTSTFPEPQLLQSIQRNIRENLQQAQVRDPPLFLISCFSPSFHDFPELRNTLQKDIFSIRYRDPLEIISQVCDKCISNKAFSLKEDQMLMKDLEAAVSSEDDTANLERGLQTYQKLFGVDDGSLQQVARSTGRLEMGSRALQFQDLIKMDRRLELMMCFAVNKFLRLLESSWWYGLWNVVTRYFRHQRHKLVIEIVAENTKTSLRKALKDSVLPPEIH*

>Irgq

RLLPPAQDGFEVLGAAELEAVREAFETGGLEAALSWVRAGLERLGSARLDLAVAGTTNVGLVLDMLLGLDPGDPGAAPASAPTGPTPYPAPERPNVVLWTVPLGPTATSPAVTPHPTHYDALILVTPGAPTEENWAQVRSLVSPDAPLVGVRTDGQGEDPPEVLEEEKAQNASDGNSGDARSEGKKAGIGDSGCTAARSPEDELWEVLEEAPPPVFPMRPGGLPGLGTWLQHALPTAQAGALLLALPPASPRAARRKAAALRAGAWRPALLASLAAAAAPVPGLGWACDVALLRGQLAEWRRALGLEPAAVARRERALGLAPGVLATRTRFPGPVTRAEVEARLGSWAGEGTAGGAALSALSFLWPTGGAAATGGLGYRAAHGVLLQALDEMLADAEAVLGPPEPNQ*

**PROTEIN SEQUENCES OF ZEBRAFISH IRG FAMILY MEMBERS**

>irge1

MPEKEEDKNENLYIISSEFLDIMSNATDDPDSISEDMKEVIDAKPKEKTRKLKDKLTELENVTLNMAITGMTGAGKSSFVNALRGLRDDDEGAASTGTTETTMKPNMYEHPFMPNVKIWDLPGIGSPKFRAKKYLKDVNFHMYDFFLIVTSERFRENDIELAKAINKSNKLFYFIRTKIDNDVRAESNKRNFDERVLLDKIREDCKVNLLKLNISKIFLISSFHLERYDFQKLVNTLEEELPKNKRFALIQSLPVYSLETLTKKITYFKKLIWLNAVGAGVGAFPPIPGVSLAVDYGIMKKFFKQVFMAFGLSNQALQVLSERVNKPVEVLNAAKTSRFKDGVTDRILIDMMSNPVIAITKTLGTIMALLPGGALPAGGAAVASVHYLLNVGLKEMADDTRKVLVVSQLA*

>irge2

MKIQKQKQELSNSSKPDTHSHSTAKENVSLKSANTVQVEHIYEMPDVHLNSSAEYINEMECVIEQNKQLGNVTLHVAVTGSTGAGKSSFINAIRGLTSDDENAAPTGVTETTLVPTMYRHPTMPNIELWDLPGTGSPKFKAKKYLKDVKLETFDFFIIISSERFKENDIMLANAIKERKKLFYFLRSKIDNDIHAESHRKDFDEQKVLSHIRENCHRNLKDIDDPHAFLICSFELHKYDFQTFVDTLEKQLPDHKRDALILSLPIYSSKILEEKIEIFMKQTWSAAVASGSVAVVPVPGLSMACDAAILLGFFTKCYYAFGLDEKSIDKLSVRVNNLSLKAIRRSPLVVAIGQKKLTNKELSALTSKEAAVKFAWSMVPVVGSIKTAQMSYSTTLNLLRTGVQDLAETAS*

>irge3

METQDPAIAEAVQASGESTLEKATAKAKESFDQFMNVSLNIAVTGKTGSGKSSFINALRGLKDDDEGAAPTGVTETTMEPNMYEHPAMPNVKIWDLPGIGSPNFKADKYLKDVKLKNYDFFIILNSERFMQNDVMLAKEIRKQKKNFYFVRSKIDNDISAEQRKKTFDEQRVLCTIREDCLKNLKQLGDPKVFLISSFDLEKYDFEELQNTLAEELPVHKRNALLQAWPVCSAASLEMKIKMFEGVIWAASLASAGIAVVPLPGLSAACDTGMVALFLTRCYFAFGLDDGSLARLSEKINKPLVGHLAKSKIASAIQEKALTRLQVSGTLVVLFSAEYVASLVPGVGSVAAAGLSFGTTYYLLRSGLKELANVAREIRKEVLDSVR*

>irge4

MTDDSSADMNFSGALQRLGESDPNAAAVKAKEELDRLDSVTLNIAVTGEAGAGKSSFINALRDLSDEDENSAPTGLTETTKKATMYTHPTKPNVRLWDLPGIGTPNFKANQYLKDVKFETYDFFIIISSERFKENDVYLAKEIQKKQKRFYFVRNKIDNDICSVANGKINEQQLLCAIREDCYRNLKEVGNPKVFLISSFDLRKYDFNLVGTLESELSDQKGFALVQSVPVYSLAMLEKKKALLEKFIWLAALASSACTLVPNQFISLITDKAILIVYLIGCHYALGLNEKSLKQLSERTNKPVSLLKLAIKSPVSLAVLDRMRISPMAKPVKSLEDLLDSKNLAVNVQNTADAFRNSHTNLTRALNEMIKDMRQVLQVAGLDE*

>irge5

KEEEDENENLYIVSSEFINIMSNATDDPDSISVDMKEVIDAKPNEKTTKLKDKLTELENVTLNMAITGMTGVGKSSFVNALRGLRDDDKDAAFTGTTETTMKPNMYEHPFMPNVKIWDLPGIGSPKFRAKKYLKDVNFHMYDFFFIVTSERFRENDIELAKAIKKSNKLFYFIRTKIDNDVRAESYKRNFDEPMLLDKIREDCKVNLLKVRISKIFLISSFHLERYDFQKLVNTLEEELPKNKRFALIQSLPVYSLEALTKKITYFKKLIWLNAVGAGVGAIAPIPGVSLAVEYVIMKKFFKQVFMAFGLSNQALEVLSGRVNKPVKVLKAAKTSRFKDGITEHILMDMISNPVIAIAVTLGTIMALLPGGALPAGGTAVATVHYLLNVGLREMADDTRKVLAISQLA*

>irge6

MECVIEQNKQLGNVTLHVAVTGSTGAGKSSFINAVRGLTSDDENAAPTGVTETTLVPMMYKHPTMPNVELWDLPGTGSPKFKAKKYLKEVKLETFDFFIIISSERFKENDIMLANAIKERKKLFYFLRSKIDNDIHAESHRKDFDEQKVLSHIREDCHRNLKDMDDPHVFLICSFELHKYDFQTFVDTLEKQLPDHKRDALILSLPIYSSKILEEKIEIFMKQTWSAAVASGSVAVVPVPGLSMACDAAILLGFFIKCYYAFGLDEKSIDKLSVRVNNPSLKAIRRSPLVVAIGQKKLTNKELSALTSKEAAVKFAWSMVPVVGSKKTAQMSYSTTLKLLRTGVQDLAETAREVLKAAGVTGVY*

>irgg1

MFFSRLCMPAKVQEDHLGTIRDVFAGESPETIPHRLISLLEVFDRFKIDIAVTGDSGAGKSSLINAILGLKPDDKGAAQTGAIETTKQATMYQQSNLPHIRLWDLPGMGTPSFASKSYVKMMNFDLYDMFMVVISERVRENNMLLVDEIDKRKKPFYFIRTKIDNDVKSQRRKSKFSETQALEQMRQDCEKYLKEKKLDPHIFLVSTHDTHNYEFQKFISTFKDEVFKIRAEEFSGFLDKMLHGGWLKAR*

>irgq1

MLHGGWLKARYATQHVQQTEKLETEDITKLQNMYKSTGFGAAKVSAVLEALSHFQLDVAVLGETGSGVSTLVNALVGLENEESSGAGASISNPALSPVYPDVRFWDISGIEAVMDYSVFEMKQAMKCYDFYIIIVSDWEKVRHVKLAKEVEKLRKHYLLVQTKVDSCLQTQGDLCCEETEILDGLRAQFTQELQREKLSEQQMFLINSQDRSAFDFVSLESALSSDLNTIRTSAFAYYIARTVKENL*

>irgq2

MADVIKGLNLLETLKESIEKNNISDIRDALEDMLISRINIAIAGERNAEKATFINSLRGLSQEDEGAAQNPPSAAPEELAVFTNPKHPDFRLWDLPPISSDANFKPEDYIERFKATRYNAIILTSTDRPSANSVAVWKEVRSLQKETVYFVLLASVKDTEKSLEAKKAASLDVLKAEGVPLPKVFLVQPSALEKLDFLTFLEVMRGDLPEIRAHALLLALPTFSSSLVTQKKDAFKALVWAAASLSGGVSAIPVPLVSSMVDATVGVRILVKAQISLCLDDESLQRLARQRGLDPAKLKALRTCALSVEVSKSEVKRRLAEAEKDTSTATTRLVELAIPRQARSVSRSFTVMLQALNNAIDDMGADAEKVVAMVTGERQ*

>irgq3

MAIQCTHRICSYLTNSLFFRFVVSTALRSMKINQDDLDQISKLSQTRDFTDNPSKLQAILGALDHFRLDVGVLGETGCGSSSLINALLGLKNSNETAALTGVTETTKEAVEYALPDSHNIRFWDLPGLGKIGDLNSLSANAFSSSEGQQVASVLALCDGYIHILVSPLRVRLRTIQLLQQASSMGKECYLVISMVDLIEDKAVEEVRQWTEKVLSKLDIQQSLFLVSANYPETLDLAKLKGMLKAAIPSHKKVALARYVSKQLDEDVFWKRSDSCKFM*

>irgf1

MATFEDYCVITQEDLDDIKDSISTQDLPSAVNTIKEYLKQQDLVELNIGVTGESGSGKSTFVNAFRGLGDEDEGSAETGPVETTMEPEVYIHPKYHNVKVWDLPGIGTPNFKADEYLELVEFERYDFFIIIASDRFRECHTQLAKEIMRMGKKFYFVRSKIDASITAEKKKKNFDQKKTLDSIRKDCINGLRKIGIEDPIVFLISGWELSKYDLNLLQDRMEKELPQHKRRVLMLALPNITLEINEKKKKALEENIRKVAFLSACVALFPLPGLSISADIAIIAEELRKYYSAFGLDDPSLQKLCERSGKTVEELKSLMKSPLHHGINPSSILTLLGAASVLISEDAVELLVSFIPIIGSVVAGGLSYLTVSGMLKKALNEIAEDARNVLMASLETEV*

>irgf2

VDALEHLYEIKVEDKLKEIKEILYTQDLPTAFGTISNYFKETSLVLNIGVTGESGSGKSTFVNAFRGLGDEDEGSAKTSSVVTTAEPEVYFHPKYENVKLWDLPGIGTPNFKADKYLELVEFERYDFFIIIASDRFRECHTQLAKGIMRMGKKFYFVRSKIDASITAEKKKKNFDQKKTLDSIREDCENGLRKIGIEYPVVFLISGWDLGKYDLNLLQEMMEKEILKCKRILLKSALLNVKQEVIEQRKDTLKRNIERVTEQSVAITDVHLPGLSISVNVDIIAEELTKYYSEFGLDDQSLQKLCERSGKTIEELKSLMKSPLCYGINTSLIINLLEAEVPKIENEYFLSFMPFIGTEIKKIKSSVAVSSMLKTALNVIAEDIRNVI*

>irgf3

MDILEDYDIITQNDLEEIKESISTEDLPTAVSRIREYLRKQDLVELNVGVTGESGSGKSTFVNAFRGLGDEDEGSAETGVVETTMEPKAYNHPKIQHVKVWDLPGIGTPNFKADEYLQQVEFERFDFFIIIASDRFKECHTHLAKEIMRMGKKFYFVRSKIDASITAEKRKKNFDLKKTLDVIREDCVNGLRKIGIEDPVVFLISNFELGKYDLNLLEEKMEEELPQHKRRVLLLALPNITQEINEKKKEALGQNIGKVAILSACVAAVPIPGLSVAVDLVIVKREIEIYYSTFGLDDPSLQMLCERSGKTIEEFKSLMKSPLRGGINPASLLSLVGAVSVVGAESTVEYILSLVPILGTVVAGGLSYLTVSTMLRRALNDIAEDARNVLNASLETEV*

>irgf4

MSNISQKVVLLFAEQEELVDLRKAISTQDLPTAINTIKECLRKQDLVELNIGVTGESGSGKSTFVNAFRGLGNEEKGSAETGFEETTMEPKDYIHPNFKNVRLWDLPGIGTPNFKAKDYLKLVKFERYDFFIIISSDRFKEHHSLLAEEIVRLRKTFYFVRSKIDQSIDSEKYKKTFDQEKMLDNIRDKCKSELSKIVKDPAVFLISCNELNKYDFQLLQERMETELPLHKRRVLMLALPNVSLDVIKKKKEVLEKDIAKVAFISATVSAVPIPGLSVAVDVMIIKEETEKYFRGFNLDDESLQRLCDVSGKSLEEIKSLMKSPLKAGIGSYSILALLSSATLVLGGMSVLAAESALEYFLSTIPLIGSVAAAAMSYKTITLMLKKTLNDLAKDAETVFKALLETEV*

**PROTEIN SEQUENCES FOR FUGU IRG GENES**

Sequences highlighted in red are treated as an intron in the main paper because of colinearity with the other p47 GTPases. But because both “exons” are in the same frame the real proteins might contain the “intron”.

>irgf5

MVNVCVCYITVGLSVGMISRLSDFYIVTVGFALCVQVIMADSLDTTEIKEALQNNNQALAVDKIKKLLEKRANTPLNIGITGESGSGKSSFVNAFRGVDHRDNQAAPTGVVETTTEVRAYPHPSYPNVTLWDLPGIGTTRFPADQYLKHVGFERFDFFIIISATRFRENDVKLAKEIQKMGKKFYFVRSKVDNDLQNAQRSQRNFDAEQTLALIRENCKEGTKVLNPVHMCVFMWSGNDDDVISWDTGLLKEGVQAPQVFLLSNFELRRHDFHRLHATLERELPEHKRDALLVSLANMSLEIIKKKKEAFKSKIPHYAFVSAACAAVPLPGLSAAVDADLIAGVVQQYKTGFGLGRPSLQRLVAITGVPLVDLTIISSPLTLDNINTDLVLNLMSQSSAISSLTETRESYSFIPLFGIPVARKLSYEITERALHNFLDMLTEDAQDVYNRVINHINS*

>irgf6

MVNVCVCYITVGLSVGMISRLSDFYIVTVGFALCVQVIMADSLDTTEIKEALQNNNQALAVDKIKKLLERAANTPLNIGITGESGSGKSSFVNAFRGVDHQDNQAAPTGVVETTTEVRAYPHPSYPNVTLWDLPGIGTTRFPADQYLKHVGFERFDFFIIISATRFRENDVKLAKEIQKMGKKFYFVRSKVDNDLQNAQRSQRNFDAEQTLALIRENCKEGTKVLNPVHMCVFMWSGNDDDVISWDTGLLKEGVQAPQVFLLSNFELRRHDFHRLHATLERELPEHKRDALLFAMPNMSLEIIEKKKEAFKSKIPHYAFVSAACAAVPVPGLSVAVDGALIAGVVQQYKTGFGLDGPSLQRLADSTGVPLEDLTSVVRSPLSLNTIDKAFILKLLLQSAAVAGLMLAEEGLKFIPLFGTLVASTLSYKVTEKALLDFLHMLAEDAQNVFKRALCCMNSSV*

**PROTEIN SEQUENCES FOR TETRAODON IRG GENES**

>irgf7

MADSSDIVEIKEALRNNNQALAAAKIKELLDNPSNATLNIGITGESGSGKSSFVNAFRGVDHKDEKEAAPVGVVETTVDVKEYPHPDYPNVSLWDLPGIGTTKFPADEYLKLVGFEKFDFFIIISDTCFRKNDVKLAKEIQKMGKKFYFVRSKVDNDLLNAQRSQRDFDPEKTLSRIRDNCKKGLLNAGVQAQVFVLSNFELQRYDFHLLHETLERELPEHKRNVLLVAMPNISLEIIEKKKEAFKSKIPLWAFVSAAGAVVPVPGLSVAVDLSLIVGLVQQYKTSFGLDTPSLQRLADTTGVQLTDLTSVIRSPLSLDNINAQLITQTLNQTASVAGLMAAEEGLRFFPIFGTMIAGSLSCAVIYKALSDFLEMLTDDAQYVFEKALRCMNSSV*

>irgf8

MADSSDFAEIKEALQNNNQALAAAKIKELLDNTSNTTLNIGITGEAGSGKSSFVNAFRGVDDRDEKAAPVGVVETTAEVKEYPHPNYPNVSLWDLPGIGTTKFPADEYLKLVGFEKFDFFIIISETRFRENDVKLAKEIQKMGKKFYFVRSKVDNDLQSEQRYQRDFDPEKTLSLIRENCKRGLLNAGLQAQVFLLSSFELQRYDFHLLYETLEREFPEHQRDVLLVAMSNISLEINGKKKEAFKSKIPYWALVSSVGALVPVPGLSVAVDLSLIAGLVQQYKTGFGLGRPSLQRLADTTGVQLTDLTSVIRSPLGLNIIDAELIVKALSELASVAGLMAAEEGLRFIPIFGTMIAGTLSYAATYNALSDFLKMLTEDAQNVFEKALRCMNSSV*
